# Tetramethylpyrazine nitrone activates hypoxia-inducible factor and regulates iron homeostasis to improve renal anemia

**DOI:** 10.3389/fphar.2022.964234

**Published:** 2022-10-17

**Authors:** Yun Cen, Peile Wang, Fangfang Gao, Mei Jing, Zaijun Zhang, Peng Yi, Gaoxiao Zhang, Yewei Sun, Yuqiang Wang

**Affiliations:** ^1^ Department of Intensive Care Unit, The First Affiliated Hospital of Jinan University and Institute of New Drug Research, Jinan University College of Pharmacy, Guangzhou, China; ^2^ Institute of New Drug Research and Guangzhou Key Laboratory of Innovative Chemical Drug Research in Cardio-cerebrovascular Diseases, Jinan University College of Pharmacy, Guangzhou, China

**Keywords:** tetramethylpyrazine nitrone, renal anemia, hypoxia-inducible factor, AMPK/mTOR pathway, iron homeostasis

## Abstract

Renal anemia is one of the most common complications of chronic kidney disease and diabetic kidney disease. Despite the progress made in recent years, there is still an urgent unmet clinical need for renal anemia treatment. In this research, we investigated the efficacy and mechanism of action of the novel tetramethylpyrazine nitrone (TBN). Animal models of anemia including the streptozotocin (STZ)-induced spontaneously hypertensive rats (SHR) and the cisplatin (CDDP)-induced C57BL/6J mice are established to study the TBN’s effects on expression of hypoxia-inducible factor and erythropoietin. To explore the mechanism of TBN’s therapeutic effect on renal anemia, cobalt chloride (CoCl_2_) is used in Hep3B/HepG2 cells to simulate a hypoxic environment. TBN is found to increase the expression of hypoxia-inducible factor HIF-1α and HIF-2α under hypoxic conditions and reverse the reduction of HIFs expression caused by saccharate ferric oxide (SFO). TBN also positively regulates the AMPK pathway. TBN stimulates nuclear transcription and translation of erythropoietin by enhancing the stability of HIF-1α expression. TBN has a significant regulatory effect on several major biomarkers of iron homeostasis, including ferritin, ferroportin (FPN), and divalent metal transporter-1 (DMT1). In conclusion, TBN regulates the AMPK/mTOR/4E-BP1/HIFs pathway, and activates the hypoxia-inducible factor and regulates iron homeostasis to improve renal anemia.

## Introduction

In the United States, anemia is twice as prevalent in patients with chronic kidney disease (CKD) than in the general population (Stauffer and Fan, 2014). Anemia is a widespread and problematic complication in CKD (Kassebaum et al., 2014; St Peter et al., 2018). Hypertension is not only the main risk factor but also one of the most common co-morbidities of diabetic kidney disease (DKD) (Stanton, 2016). Every 10 mmHg increase in mean systolic blood pressure induces a 15% increase in developing microalbuminuria (Retnakaran et al., 2006). Hence, diabetic patients with concomitant hypertension have a much higher risk of developing DKD than diabetic patients without. DKD patients are 2–10 folds more likely to have anemia than non-diabetic nephropathy patients (Loutradis et al., 2016; Astor et al., 2002). The reduction of erythropoietin (EPO) production by renal tubular fibroblasts is the primary cause of CKD-related anemia (Olmos et al., 2018). When kidney function decreases, patients have inadequate serum iron, resulting in decreased transfers of iron into the bone marrow, further impairing erythropoiesis (Mikhail et al., 2017; Gafter-Gvili et al., 2019).

Anemia occurs at an early stage and can be observed even before any demonstrable changes in renal function take place (Thomas, 2007). The etiology and pathophysiology of anemia are multi-factorial, including iron deficiencies, chronic diseases, and bone marrow diseases (Crathorne et al., 2016). EPO analogues and iron replacement are the current treatments for anemia, but the Food and Drug Administration (FDA) has issued a warning for the usage of EPO analogues because their usage results in a greater risk of serious side effects (Besarab et al., 2015).

Although a relative deficiency of EPO production is the main cause of anemia in CKD, iron metabolism is closely regulated at various stages of the red blood cell (RBC) life cycle. Human serum iron levels are tightly controlled by iron efflux transporter FPN and the hormone hepcidin (Billesbølle et al., 2020). The process by which erythroblasts disintegrate into reticulocytes is iron-dependent (Yilmaz et al., 2011). Serum transferrin (Tf) binds to the iron in the gastrointestinal tract. Following this, iron is transported to the liver or spleen for storage or to the bone marrow for erythropoiesis (Wish, 2006). Divalent metal transporter-1 (DMT1) and duodenal cytochrome b reductase (DCYTB) are activated by HIF-2α during HIF signaling regulation of transferrin receptor (TfR) and iron transport gene Tf expression (Batchelor et al., 2020).

HIF-1α synthesis is regulated by mammalian target of rapamycin complex 1 (mTORC1) on a translational level through co-operative regulation of 4E-binding protein 1 (4E-BP1). Cobalt metal is genotoxic, creating oxidative DNA damage through reactive oxygen species and inhibits DNA repair (Simonsen et al., 2012). Cobalt (Co^2+^) stabilizes the transcriptional activator HIF and therefore imitates hypoxic conditions and urges EPO production (Simonsen et al., 2012).

TBN is a novel nitrone derivative of tetramethylpyrazine, a principal active ingredient of the herbal medicine Ligusticum Chuanxiong armed with a powerful free radical scavenger (Sun et al., 2008; Tian et al., 2010; Sun et al., 2012). The structure of TBN is shown in Supplementary Figure S1. Our previous studies have found that TBN was effective in improving renal functions in DKD at the early stage both in rats and monkeys (Jing et al., 2021), however, its effects on renal anemia have not been explored. As DKD and renal anemia are commonly concomitant, herein we investigated whether TBN is effective in alleviating renal anemia and its mechanism of action *in vitro* and in animal models of CKD and DKD.

## Materials and methods

### Animal model

All animal care and experimental protocols were approved by the Institutional Animal Care and Use Committee of the Guangzhou University of Chinese Medicine (Guangzhou, China). All animal experiments complied with the US National Institutes of Health guide for the care and use of laboratory animals (NIH Publications No. 8023, revised 1978).

The 11-week-old male SHR rats and normal Wistar Kyoto rats (WKY) were obtained from Beijing Vital River Laboratory Animal Technology Co, Ltd, China. The rats were housed 3–4 per cage on a 12 h light/dark cycle with standard rodent chow and water *ad libitum* in a temperature (20–25°C) and humidity (30%–50%) under the control of an animal facility. Animals (weighing 300 ± 10 g, 200 ± 10 g) were divided into the following four groups: 1) normal WKY group (WKY-Ctrl); 2) non-diabetic SHR group (SHR-Ctrl); 3) STZ-induced diabetic SHR group (SHR-STZ); 4) TBN-treated STZ-induced diabetic SHR group (60 mg/kg) (SHR-STZ-TBN 60). For diabetic rats, blood glucose levels were measured 3 days after the intraperitoneal injection of STZ (55 mg/kg, STZ, Sigma-Aldrich) and the rats with blood glucose concentrations lower than 16.7 mmol/L were excluded. Three weeks after STZ injection, rats were administered intragastric TBN (60 mg/kg) twice a day for 6 weeks.

The 8-week-old male C57BL/6J mice were obtained from Guangdong Medical Laboratory Animal Centre, China. The mice were housed 6 per cage on a 12 h light/dark cycle with standard rodent chow and water *ad libitum* in a temperature (20–25°C) and humidity (30%–50%) under the control of the animal facility. For the CDDP-induced C57BL/6J mice renal anemia model, male C57BL/6J mice were randomly divided into 6 groups: control group (Control), vehicle-treated CDDP group (Model), CDDP-TBN low-dose group (10 mg/kg, TBN 10), CDDP-TBN medium-dose group (30 mg/kg, TBN 30), CDDP-TBN high-dose group (60 mg/kg, TBN 60), and CDDP-Roxadustat (10 mg/kg) group (Roxadustat). The control group was injected with an equal volume of normal saline. The other groups were injected with CDDP (5 mg/kg, CDDP, Sigma-Aldrich) intraperitoneally once a week for a total of 4 weeks. From the fifth week of the CDDP injection to the end of the eighth week, the TBN and Roxadustat treatment groups were respectively given TBN and Roxadustat by intragastric administration, with TBN administered twice a day, and Roxadustat once every other day.

### Metabolic measurements

In the SHR, blood glucose and pressure levels were measured at the third and ninth weeks. Bodyweight was measured weekly. After 6 weeks of TBN treatment, the rats were sacrificed; blood and tissue samples were harvested and processed for the following research. Blood glucose, blood urea nitrogen (BUN), serum iron, and Tf were measured *via* an automatic biochemical analyser (Hitachi AutoAnalyzer 7100, Hitachi Co. Ltd., and Tokyo, Japan). The sera from SHR were assayed using Rat EPO ELISA Kits (BioLegend, Inc., San Diego, CA, United States) and Rat Hepcidin ELISA Kits (Biovision, Milpitas, CA, United States), after which blood samples were collected and centrifuged for 10 min at 3,000 g.

### Blood pressure measurement

Systolic and diastolic blood pressures were non-invasively measured *via* a calibrated-catheter tail-cuff system (CODA, Kent Scientific, and Torrington, CT). Rats were kept in compatible size holders on a comfortable and warm pad during the measurement. Blood pressure was monitored before and after TBN administration, respectively. Blood pressure measurements were conducted at the same time each day.

### Blood analysis

Mouse whole blood collected from the caudal vein was analyzed by an Automatic five-category blood analyser. Mouse blood was taken by an abdominal aortic method and serum was separated by centrifugation with 3,000 g/10 min. In the fourth and eighth weeks after the start of the experiment, when the animals were awake, blood was collected from the tail vein, and the whole blood haemoglobin (Hb), RBC, and haematocrit (HCT) were measured with the Automatic Five-category Blood Analyser. Blood serum EPO (BioLegend, Inc., San Diego, CA, United States), serum Hepcidin (Biovision, Milpitas, CA, United States), serum catalase (CAT), and serum Glutathione peroxidase (GPx) concentrations were quantified by mouse ELISA kit.

### H&E staining and PAS staining

The kidneys and livers were excised for histological analysis and stored at −80°C. A portion of the renal cortex and right lobe of the liver were reserved for biochemical analysis. The glomerular area was measured with H&E staining and the tubular injury was assessed by PAS staining. The malnutrition inflammation scores of the liver were quantified by H&E staining. After fixing in 4% paraformaldehyde for 24 h, kidneys and livers were embedded in paraffin and cut into 5 μm slices. The tissue sections were dewaxed and then stained with haematoxylin, after which they were washed with acid-alcohol and stained with eosin (Nanjing Jiancheng Bioengineering Institute, China). For PAS staining, slides were incubated with Alcian blue and Schiff’s reagent to stain the acidic mucins and neutral mucins respectively. The slides were examined using a Fluorescence Inversion Microscope System (Olympus Corporation, CKX41).

### Immunohistochemistry assay

The tissue sections were deparaffinized and rehydrated. For retrieving antigens, slides were heated in 10 mmol/L sodium citrate-hydrochloric acid buffer (Beyotime Biotechnology, China), and then incubated with blocking buffer (10% horse serum and 3% Triton X-100) at room temperature for 2 h. Afterward, the kidney sections were incubated with primary antibody HIF-2α (Abcam, Cambridge, MA, 1:300) at 4°C overnight, then incubated with secondary antibody for 2 h at room temperature. The DAB kit was used to visualize the antibody binding in the kidney sections. The nucleus was stained with haematoxylin as a counter stain. Photomicrographs were taken by Fluorescence Inversion Microscope System (Olympus Corporation, CKX41).

### Cell culture and transfection

Hep3B and HepG2 cells were cultured in DMEM medium augmented with 10% heat-inactivated fetal bovine serum and 1% penicillin/streptomycin solution at 37°C with 5% CO_2_ and 95% air. In a hypoxic environment, the cells were incubated accompanied by 50 μM CoCl_2_ for 24 h. After CoCl_2_-induction, Hep3B and HepG2 cells were hatched with TBN (30, 100, and 300 μM) under hypoxic conditions for 24 h. In order to assess protein stability, cells were incubated in the existence of CoCl_2_ with or without TBN for 24 h and then treated with 2.5 μg/ml cycloheximide (CHx) for indicated times. In SFO stimulation experiments, Hep3B cells were incubated with 200 μg/ml SFO in presence of TBN (300 μM) under hypoxia for 24 h.

### Western blot analysis

For preparing total protein, the kidney and liver tissues were lysed in RIPA lysis buffer (Beyotime Biotechnology, China) containing 1% PMSF (Beyotime Biotechnology, China) and 1% phosphatase inhibitors (Cell Signaling Technology, Danvers, MA, 1:100). The concentration of protein was measured by BCA assay and total protein was separated by 10% SDS-PAGE and then transferred to PVDF membranes (0.45 μm, Immobilon-P, United States). After blocking with 5% skim milk for 2 h at room temperature, membranes were incubated with primary antibody HIF-1α (Cell Signaling Technology, Danvers, MA, 1:2000), HIF-2α (Abcam, Cambridge, MA, 1:1000), NRF2 (Abcam, Cambridge, MA, 1:1000), HO-1 (Novus, Littleton, Colorado, United States, 1:1000), ferritin (Cell Signaling Technology, Danvers, MA, 1:1000), FPN (Cell Signaling Technology, Danvers, MA, 1:1000), Prolyl hydroxylase inhibitor (PHD3) (Cell Signaling Technology, Danvers, MA, 1:1000), factor-inhibiting HIF (FIH) (Cell Signaling Technology, Danvers, MA, 1:1000), DMT1 (Cell Signaling Technology, Danvers, MA, 1:1000), p-mTOR (Cell Signaling Technology, Danvers, MA, 1:1000), mTOR (Cell Signaling Technology, Danvers, MA, 1:1000), p-AMPK (Cell Signaling Technology, Danvers, MA, 1:1000), AMPK (Cell Signaling Technology, Danvers, MA, 1:1000), p-ULK1 (Cell Signaling Technology, Danvers, MA, 1:1000), ULK1 (Cell Signaling Technology, Danvers, MA, 1:1000), LC3 II (Cell Signaling Technology, Danvers, MA, 1:1000), GADPH (Cell Signaling Technology, Danvers, MA, 1:1000) and β-actin (Cell Signaling Technology, Danvers, MA, 1:1000) overnight at 4°C. Then, the PVDF membranes were incubated with anti-rabbit IgG antibody (Cell Signaling Technology, Danvers, MA, 1:2000) and anti-mouse IgG antibody (Cell Signaling Technology, Danvers, MA, 1:2000) respectively for 2 h at room temperature. Immunoreactive bands were visualized by the ECL detection system (Amersham Imager 600, GE, United States).

### Immunofluorescence microscopy

Before being fixed in paraformaldehyde for 15 min at room temperature, cells were cultured and incubated on confocal small dishes. After washing three times with PBS, cells were treated with 0.3% v/v Triton X-100 in PBS for 10 min. After washing three times with PBS, cells were blocked with 10% v/v FBS in PBS for 1–2 h. Cells were then incubated with primary antibodies HIF-1α (1:300, diluted in Immune Primary Antibody Diluent) and HIF-2α (1:300, diluted in Immune Primary Antibody Diluent) overnight. Next day, the cells were washed three times before being incubated with fluorochrome-conjugated secondary anti-rabbit antibody or anti-mouse antibody, and DAPI for 1–2 h, and were washed three times with PBS and dropped anti-fluorescence quencher. Cells were examined and scanned on a fluorescence microscope.

### Intracellular reactive oxygen species detection

Intracellular reactive oxidative species (ROS) were determined using 2′, 7′-dichlorofluorescin diacetate (DCFH-DA; Sigma-Aldrich, St Louis, MO, United States). Hep3B cells were incubated with SFO and TBN for 24 h and then 10 mΜ DCFH-DA was added at 37°C for 30–40 min. After washing, the ROS production was measured using quantitative measurements by fluorescence microscopy (FilterMax F3 Multi-Mode Microplate Readers, Molecular Devices, and Sunnyvale, CA, United States).

### QPCR analysis

Total RNA was extracted from HepG2 cells using Trizol according to the manufacturer’s protocol. Complementary DNA was synthesized using the All-in-One TM First-Strand cDNA Synthesis kit. Quantitative reverse transcription-polymerase chain reaction (qRT-PCR) was carried out in an ABI 7900 real-time PCR system (Illumina, San Diego, CA, United States) for 40 cycles using the All-in-One TM qPCR SYBR Green Mix. The results were expressed as the means of fold changes in target gene expression relative to GADPH, *n* = 3.

### Statistical analysis

GraphPad Prism 8.0 software (San Diego, CA, United States) was used to analyse the data. The data were expressed as the mean ± SEM. Comparisons among multiple groups were analysed using one-way ANOVA or two-way ANOVA by Dunnett’s test, as appropriate. *p* < 0.05 was considered statistically significant.

## Results

### Tetramethylpyrazine nitrone alleviated streptozotocin-induced kidney damage and ameliorated anemia in the spontaneously hypertensive rats

**FIGURE 1:**
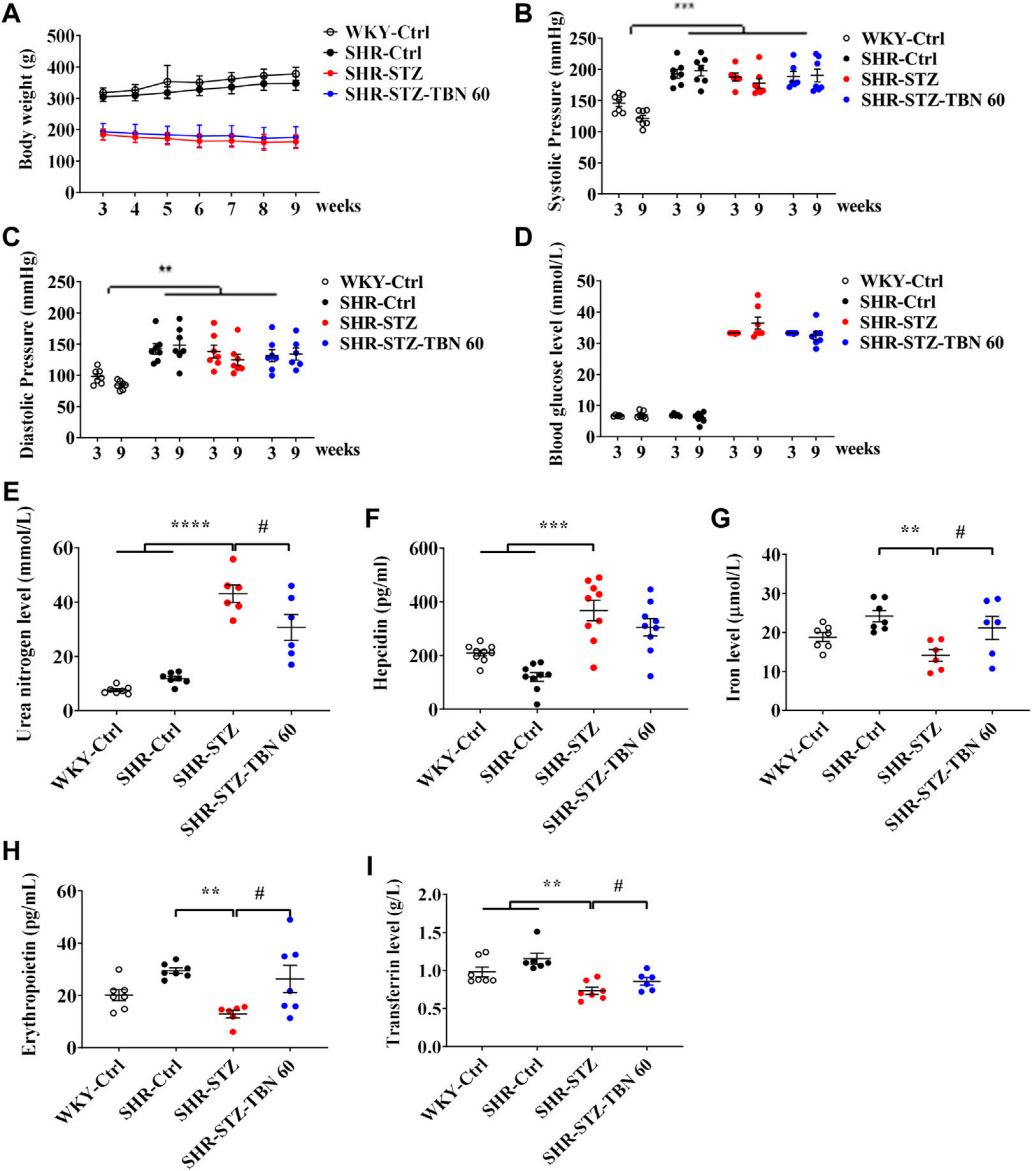
Effects of TBN on STZ-induced diabetic SHR rats. 11-week-old SHR rats are injected with a single dose of STZ (55 mg/kg) to induce diabetes. Shown are the effects of STZ and TBN on **(A)** body weight; **(B,C)** systolic pressure and diastolic pressure; **(D)** blood glucose level; **(E)** urea nitrogen level in diabetic-SHR rats treated with TBN. The changes of **(F)** hepcidin, **(G)** iron level, **(H)** EPO, and **(I)** Tf level in the serum of SHR rats after TBN treatment (*n* = 6–9 for each group). Data are presented as mean ± SEM; ** *p* < 0.01; *** *p* < 0.001; **** *p* < 0.0001 vs. WKY-control, SHR-control; ^#^ *p* < 0.05 vs. SHR-STZ. WKY, Wistar-Kyoto; STZ, streptozotocin; SHR, spontaneously hypertensive rat; EPO, Erythropoietin; Tf, transferrin.

After injection of STZ, the body weight of rats was significantly reduced about 39.4%, which indicated that STZ induced a stable and reliable diabetic mellitus condition in SHR rats (Figure 1A). The systolic and diastolic pressures of SHR rats were significantly higher than in the WKY rats and TBN treatment did not affect blood pressure (Figures 1B,C). In SHR-STZ rats, the blood glucose level was approximately four times higher than that of the SHR-Ctrl rats. There was a non-significant trend of decrease in the blood glucose level in the TBN-treated than untreated SHR-STZ group at 9-weeks (Figure 1D). The urea nitrogen level was significantly elevated in the STZ-induced rats compared to that of the SHR rats (Figure 1E). TBN treatment significantly reversed the increase in circulating urea nitrogen levels (Figure 1E).

We found that the levels of EPO and iron in SHR rats were increased compared with those of WKY rats before STZ treatment although the difference did not reach statistical significance. After STZ treatment, the hepcidin level was suppressed in SHR rats. STZ treatment promoted hepcidin expression and TBN alleviated the effects of STZ (Figure 1F). Furthermore, EPO and iron levels in the serum of SHR-STZ group were significantly lowered than those that in the SHR-Ctrl group. However, TBN treatment increased the levels of both serum iron level and EPO (Figures 1G,H). Tf is a biomarker for diagnosing anemia and monitoring treatment. In the DKD-SHR rats, expression of Tf was much lower than that in SHR rats; TBN elevated Tf levels by a statistically significant amount (Figure 1I). These results demonstrated that TBN ameliorated anemia in the diabetic SHR.

### Tetramethylpyrazine nitrone alleviated cisplatin-induced anemia in the C57BL/6J mice

**FIGURE 2:**
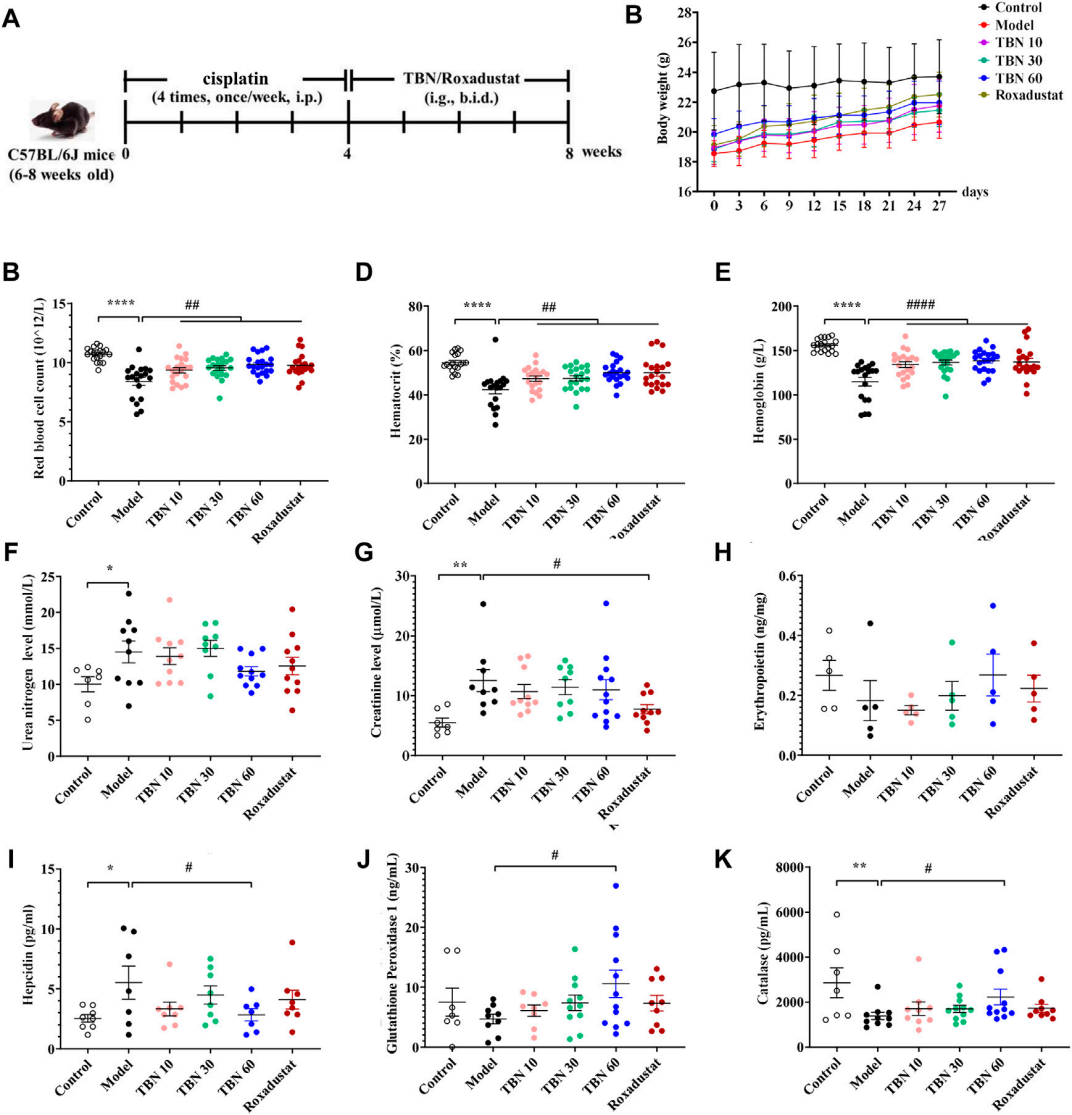
TBN ameliorates renal anemia in CDDP-induced mice. **(A)** Experimental flow chart, **(B)** Body weight. Blood measurements including **(C)** RBC count, **(D)** HCT and **(E)** Hb in C57BL/6J mice [*n* = 19–21 for each group in Figure **(A–E)**]. Levels of **(F)** Urea nitrogen level, **(G)** Creatinine after TBN treatment in C57BL/6J mice. Levels of **(H)** EPO, **(I)** hepcidin, subsequent to TBN treatment in C57Bl/6J mice. The effect of TBN in **(J)** GPx, **(K)** CAT (*n* = 7–8 for control group in Figure **(F–G)**, **(I–K)**; *n* = 7–12 for the other group in Figure **(F–G)**, **(I–K)**; *n* = 5 for each group in Figure **(H)**. Data are presented as mean ± SEM; * *p* < 0.05; ** *p* < 0.01; **** *p* < 0.0001; vs. Control; ^#^ *p* < 0.05; ^##^ *p* < 0.01; ^####^ *p* < 0.0001 vs. vehicle-treated CDDP group. CDDP, cisplatin; RBC count, red blood cell count; HCT, hematocrit; Hb, hemoglobin; EPO, Erythropoietin; CAT, catalase; GPx, glutathione peroxidase. Model: vehicle-treated CDDP group.

In order to explore whether TBN ameliorates anemia in the CKD anemia model, CDDP was administered to produce the CKD anemia model (Wu et al., 2018). The flow chart of the experiment was shown in Figure 2A. Four weeks after CDDP administration, the body weight of mice was significantly decreased (approximately 25%) as shown in Figure 2B. In contrast to normal mice, the CDDP-induced mice exhibit reduced RBC count, HCT and Hb. The TBN treated CDDP-induced mice exhibit higher RBC counts, HCT, and Hb than those in the CDDP-induced (Model) TBN-untreated group. Hb levels in the CDDP-administered anemic mice (120 g/L) were up-regulated by TBN to close to the normal levels (160 g/L), performing on par with the positive drug Roxadustat, and treatment with TBN stopped the development of CDDP-induced anemia (Figures 2C–E). The decreased RBC count and HCT level were reversed with TBN treatment dose-dependently, and the effects of a high dose of TBN (60 mg/kg) were similar to that of the positive control Roxadustat. There was no statistically significant difference in urea nitrogen and creatinine between the cisplatin-induced renal anemia group and the TBN-treated groups (Figures 2F,G). Roxadustat similarly reduced the creatinine level (Figure 2G).

There was a non-significant trend of decrease in the EPO levels after 8 weeks of CDDP administration in the model group than the control group. TBN treatment caused a dose-dependent increase in EPO levels. However, there were no statistically significant differences in serum EPO levels between the vehicle-treated CDDP group and the TBN- or Roxadustat-treated groups (Figure 2H). Hepcidin, which plays an inhibitory role in the regulation of iron balance in the body, leading to iron deficiency and anemia due to the premature overexpression of hepcidin, is a biomarker for the diagnosis and treatment monitoring of anemia. TBN diminished CDDP’s effects, and CDDP treatment increased hepcidin expression (Figure 2I). These data showed that TBN reduces the severity of anemia in C57BL/6J mice.

We had found that TBN had significant free radical scavenging activity against some of the most damaging radicals, including superoxide, hydroxyl and peroxynitrite (Zhang et al., 2018). To investigate whether TBN influenced peroxide levels, the amount of GPx, an important peroxide decomposing enzyme widely present in the body, was measured in the sera of C57BL/6J mice. TBN (60 mg/kg) significantly increased the level of GPx in comparison to Roxadustat. Similar to the increase in GPx, TBN also increased the level of CAT at high doses (Figures 2J,K).

### Tetramethylpyrazine nitrone improved anemia through activating hypoxia-inducible factor in streptozotocin-induced SHR rats and cisplatin-induced C57BL/6J mice

**FIGURE 3:**
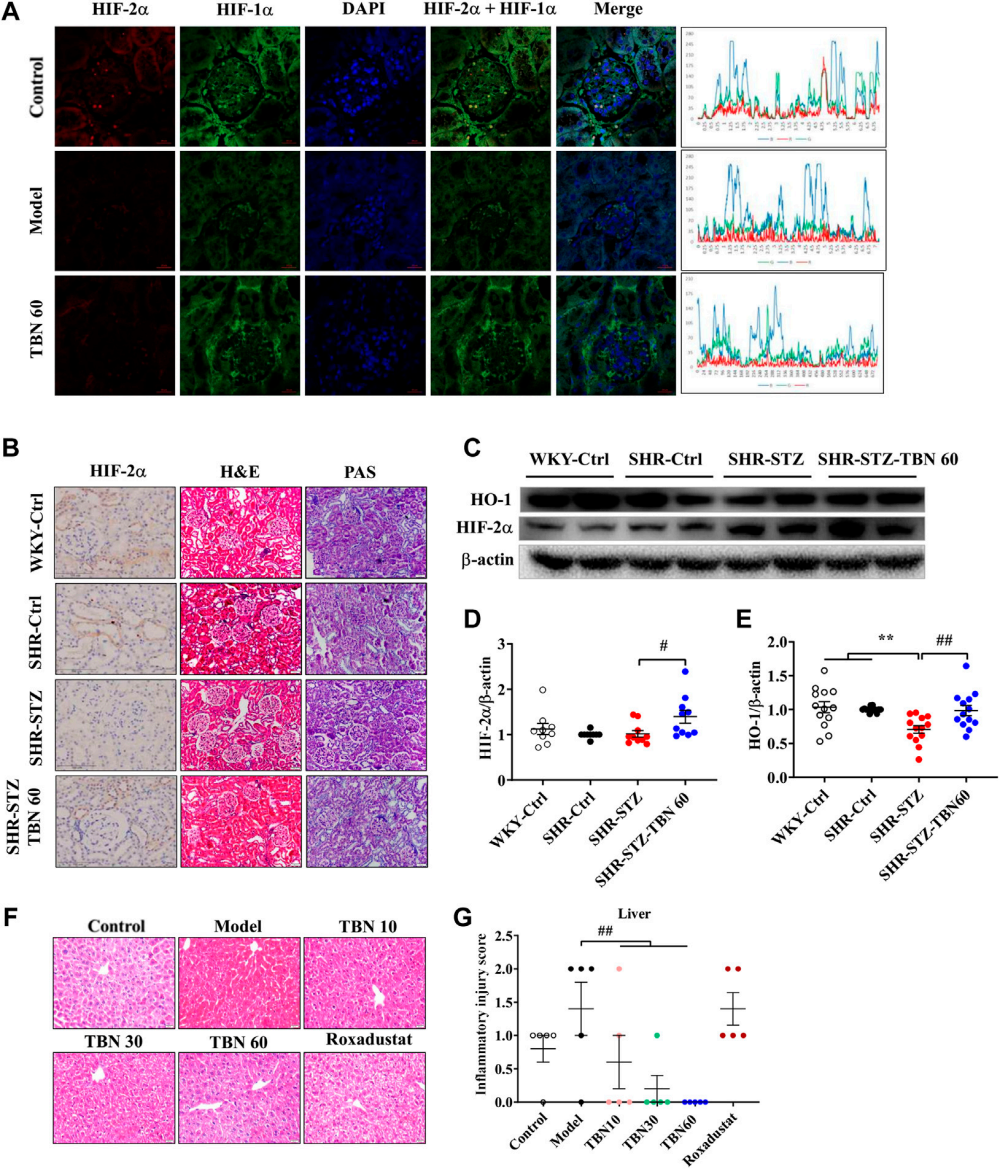
TBN increases the expression of HIF-1α and HIF-2α in CDDP-induced anemia C57BL/6J mice and SHR rats. **(A)** Representative images of the expression of HIF-1α and HIF-2α by immunofluorescence co-staining experiment, (original magnification: ×630). Scale bar, 20 μm. The Y-axis is the average value of fluorescence intensity obtained by immunofluorescence analysis through Image J software. The peaks as shown along with the immunofluorescence images represent the highest expression of the whole images. The X-axis is the length of the images. The formula of fluorescence intensity in a specific area is as follows: Mean fluorescence intensity (Mean) = Total fluorescence intensity (IntDen)/Area of the region (Area). Mean: Mean gray value, IntDen: Integrated Density. **(B)** Representative images of H&E staining and PAS staining (original magnification: ×200) of kidney tissue. Scale bar, 50 μm. Representative Immunohistochemistry staining of HIF-2α (original magnification: ×400). Scale bar: 100 μm. **(C–E)** Representative western blot of HIF-2α and HO-1 (*n* = 8 for each group in Figure **(D)**; *n* = 13 for each group in Figure **(E)**. **(F,G)** Representative images of HE staining (×200) of liver tissue and inflammatory injury score in CDDP-induced C57BL/6J mice (*n* = 5 for each group in Figure **(G)**. ** *p* < 0.01 vs. SHR-control; ^#^ *p* < 0.05; ^##^ *p* < 0.01 vs. SHR-STZ. ^**##**^ *p* < 0.01 vs. vehicle-treated CDDP group. HIF, hypoxia-inducible factor; CDDP, cisplatin.

In the kidney tissues of C57BL/6J anemic mice, after immunofluorescence co-staining, areas where significant differences were found that TBN increased the synthesis of not only HIF-2α but also HIF-1α, and TBN affected HIF-1α more than HIF-2α (Figure 3A). To further explore the protective effect of TBN in STZ-induced renal injury, we investigated the pathological changes in rat kidneys through imaging. Hypertension seemed to have little effect on renal tissue when compared to what was seen in WKY rats. Remarkably, STZ induced an abnormal glomerular hypertrophy, but TBN treatment ameliorated the effect of STZ (Figure 3B). The tubular injury shown with PAS staining demonstrated that TBN treatment partially reversed the STZ damage (Figure 3B). The H&E staining and PAS staining results indicated that TBN significantly reduced STZ-induced damage in SHR rats with DKD. We evaluated the effects of TBN on HIF-2α and HO-1 in STZ-induced DKD anemia rats and the effects of TBN on HIF-1α and HIF-2α in CDDP-induced C57BL/6J anemic mice. TBN treatment increased the HIF-2α level (Figures 3C,D). HO-1 showed a similar change to that of HIF-2α (Figures 3C,E). These results suggested that TBN can prevent renal anemia by activating HIFs in STZ-induced SHR rats and CDDP-induced mice.

Next, the liver injury was analysed through PAS staining. Microscopically, the mice treated with CDDP exhibited inflammation in the hepatic lobules as well as a small amount of hepatocyte necrosis, nucleus lysis, and increased cytoplasmic eosinophilia, with no obvious inflammation in the portal area (Figures 3F,G). Remarkably, these pathological injuries were reduced after TBN treatment. The mice treated with Roxadustat exhibited little improvement (Figures 3F,G). These results indicated that TBN can improve renal function in rats and liver function in mice.

### Tetramethylpyrazine nitrone increased synthesis and nuclear expression of hypoxia-inducible factor under hypoxic conditions

**FIGURE 4:**
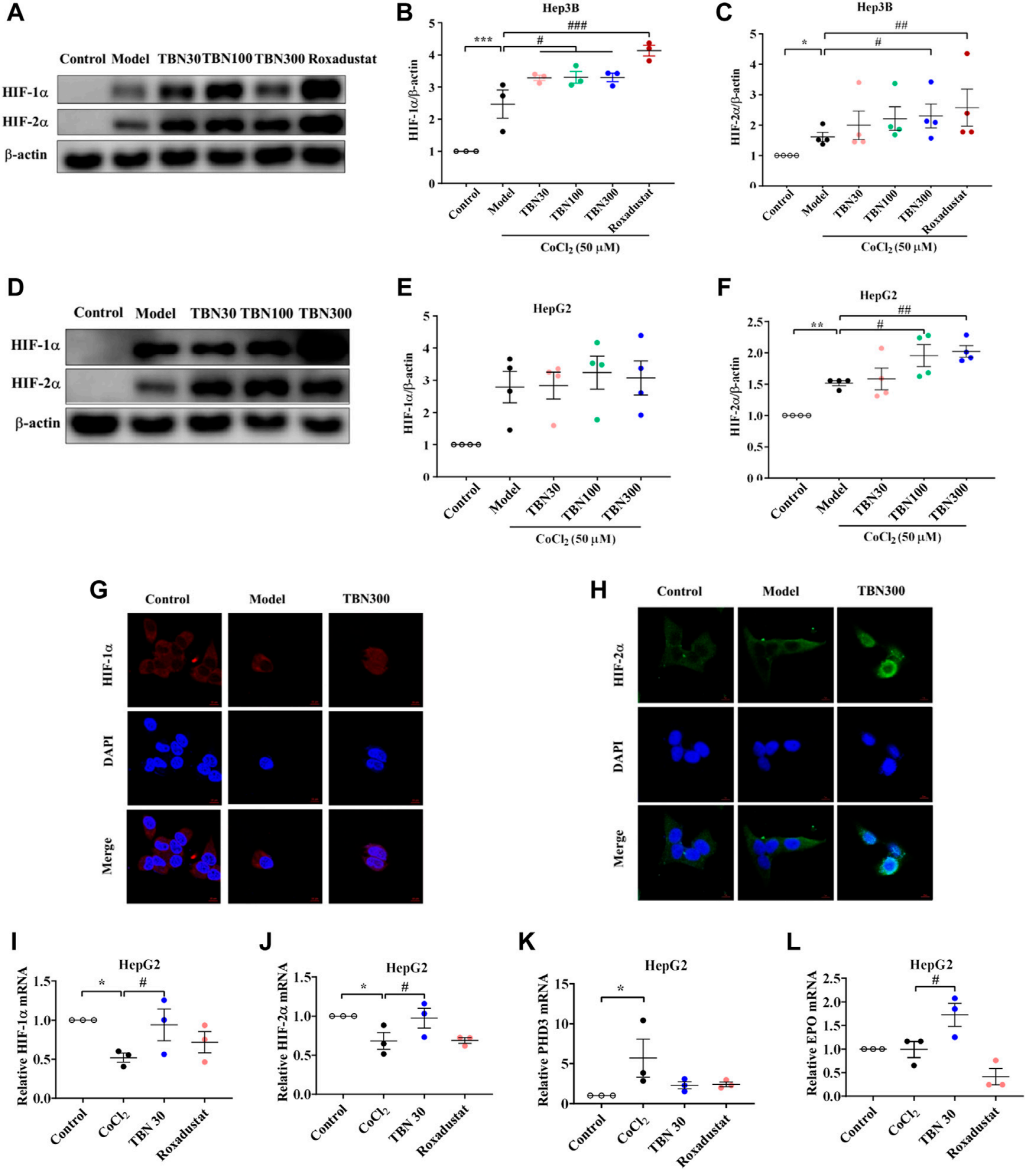
TBN increases the expression of HIF-1α and HIF-2α in CoCl_2_-induced Hep3B and HepG2 cells. **(A–C)** Effects of TBN on expression of HIF-1α and HIF-2α in CoCl_2_-induced Hep3B cells. **(D–F)** Representative western blots of HIF-1α and HIF-2α in HepG2 cells. **(G,H)** HIF-1α and HIF-2α nuclear expression in Hep3B cells, (original magnification: ×630). Scale bar, 10 μm. **(I–L)** HIF-1α, HIF-2α, PHD3 and EPO mRNA expression in HepG2 cells. [*n* = 3–4 for each group in Figure **(A–L)**]. * *p* < 0.05; ** *p* < 0.01; *** *p* < 0.001; vs. Control; ^#^ *p* < 0.05; ^##^ *p* < 0.01; ^###^ *p* < 0.001 vs. CoCl_2_-induced Model. HIF, hypoxia-inducible factor; CoCl_2_, cobalt chloride. Model: CoCl_2_-induced group.

To further confirm whether TBN increased HIFs synthesis, CoCl_2_ was used to produce hypoxic conditions. As seen through Western blot, HIF-2α expression increased significantly after TBN treatment under hypoxic conditions both in Hep3B and HepG2 cells, while HIF-1α expression increased only in Hep3B cell after TBN treatment (Figures 4A–F). Hypoxia leads to increased expression of HIF, which then binds to the EPO gene enhancer, resulting in transcriptional activation (Semenza and Wang, 1992). We found that TBN can promote both HIF-1α and HIF-2α nuclear expression in Hep3B cells (Figures 4G,H), suggesting that TBN increased EPO through activating HIF-1α synthesis and nuclear expression. Then we examined the expression of HIF-1α, HIF-2α, PHD3 and HIF-1α target genes as well as EPO, by qRT-PCR. As shown in Figures 4I–L, TBN promoted the expression of HIFs and EPO and decreased PHD3 in the hypoxic environment. These results demonstrated that TBN effectively induced HIFs synthesis and nuclear expression to regulate EPO.

### Tetramethylpyrazine nitrone regulated HIF-1α synthesis in a translation-dependent fashion

**FIGURE 5:**
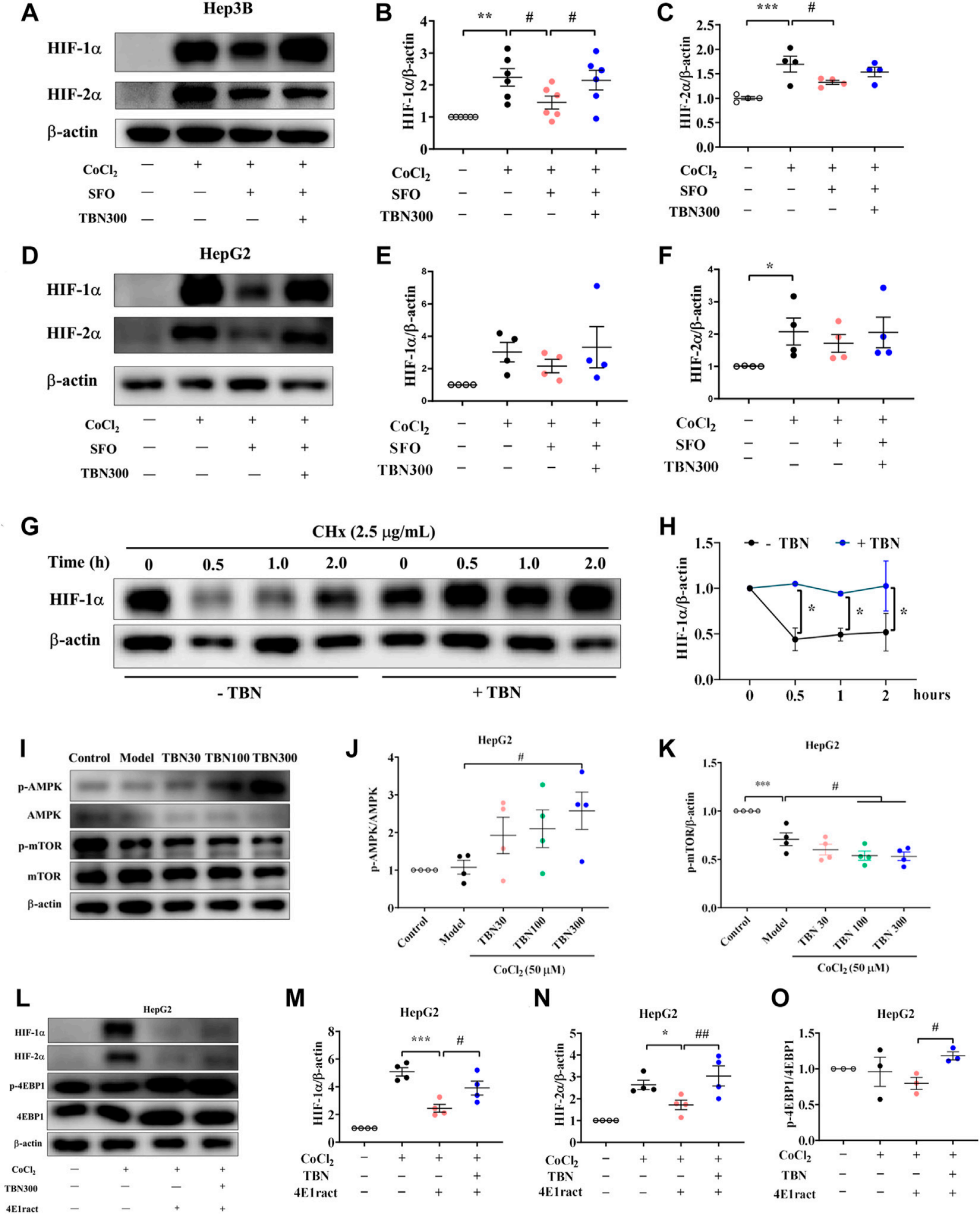
**(A–F)** TBN reverses the suppressive action of SFO on CoCl_2_-induced increments of HIF-1α and HIF-2α protein expression in Hep3B and HepG2 cells. **(G,H)** TBN effects on HIF-1α and HIF-2α protein levels in Hep3B cells by western blot analysis. **(I–K)** Effects of TBN on expression of AMPK/mTOR in CoCl_2_-induced HepG2 cells. **(L–O)** HIF-1α, HIF-2α and p-4EBP1 expression with or without 4E1rcat in HepG2 cells. [*n* = 3–6 for each group in Figure **(A–O)**]. * *p* < 0.05; ** *p* < 0.01; *** *p* < 0.001 vs. Control; ^#^ *p* < 0.05 vs. CoCl_2_-induced Model, CoCl_2_-SFO-induced group. * *p* < 0.05; *** *p* < 0.001; vs. 4E1rcat-induced group; ^#^ *p* < 0.05; ^##^ *p* < 0.01 vs. TBN group. SFO: saccharate ferric oxide; CoCl_2_: cobalt chloride; HIF: hypoxia-inducible factor.

Superfluous iron produces hydroxyl radicals through the Fenton reaction that can cause oxidative stress (Kruszewski, 2003). In earlier research, SFO administration is found to reduce EPO mRNA expression; HIF-1α and HIF-2α mRNA and protein levels are also reduced by SFO treatment in HepG2 cells (Oshima et al., 2017). The research suggests that SFO inhibited the expression and translation of HIFs and its target gene EPO. To explore whether TBN increased the number of HIFs at the translation step, we thus used SFO (200 μg/ml) with and without TBN in Hep3B and HepG2 cells to examine the change in HIF-1α and HIF-2α levels. As our results shown (Figures 5A–F), TBN reversed the down-regulation of HIFs seen with SFO, which confirms our hypothesis.

TBN regulated HIF-1α at both the mRNA and protein levels. To explore the effect of TBN treatment in the stage of HIFs’ protein synthesis, CHx, a protein translational inhibitor, is used in HepG2 cells to investigate HIF-1α levels over time. In this study, as shown in Figures 5G,H, the half-life of HIF-1α was extended to 2 h with TBN treatment, while that without TBN was 0.5 h. These results indicated that TBN further stabilized HIF-1α in hypoxic conditions.

### Tetramethylpyrazine nitrone regulated hypoxia-inducible factor *via* AMPK/mTOR to influence translation and maintain iron homeostasis

**FIGURE 6:**
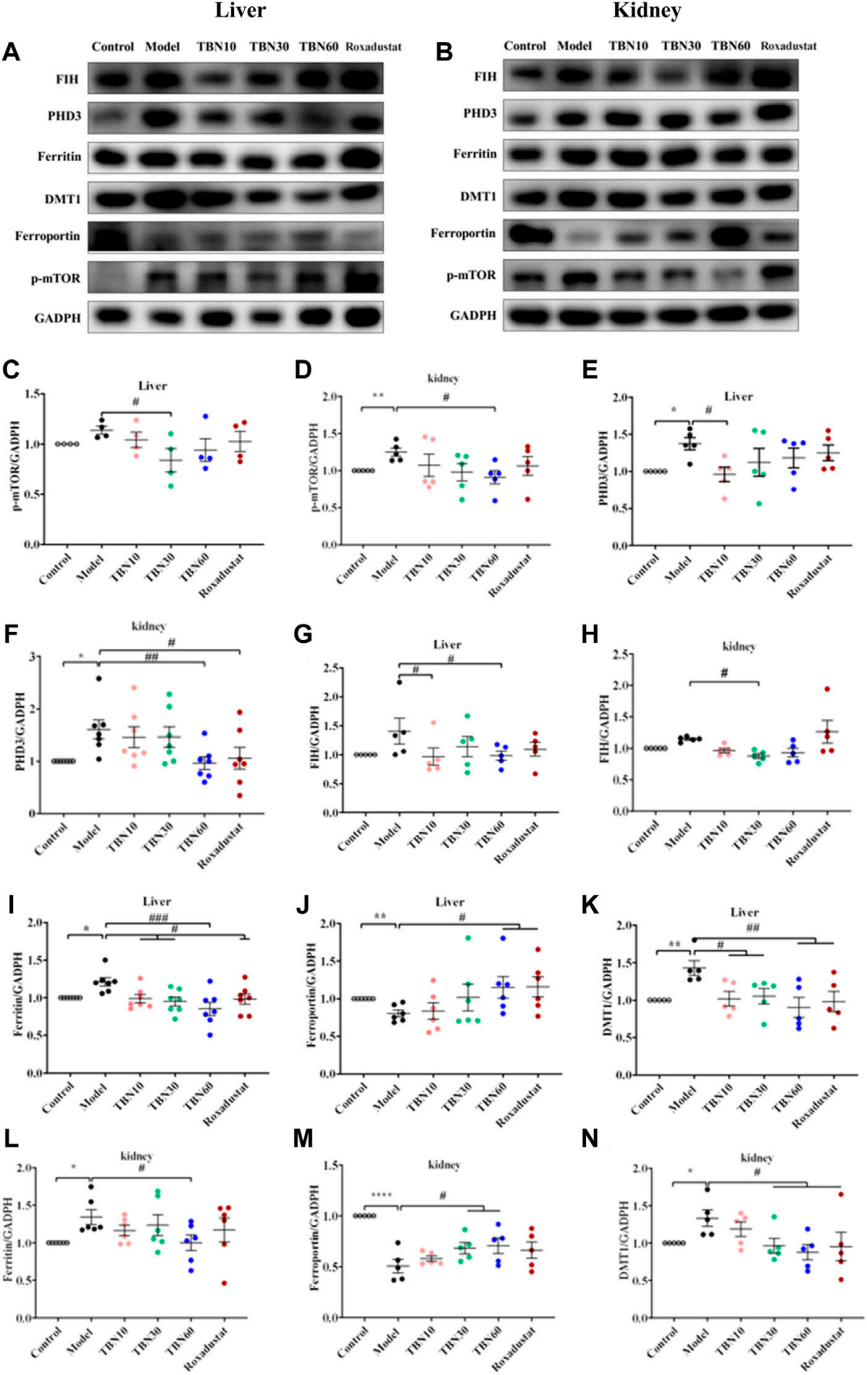
TBN effect in iron metabolism in CDDP-induced C57BL/6J mice. **(A–D)** Representative western blots of p-mTOR in liver and kidney tissue. **(A,B,E–H)** Representative western blots of PHD3, FIH of liver and kidney tissue. **(A,B,I–N)** Representative western blots of ferritin, FPN and DMT1 both in liver and kidney tissue. [*n* = 4–7 for each group in Figure **(A–N)**]. * *p* < 0.05; ** *p* < 0.01; **** *p* < 0.0001 vs. Control; ^#^ *p* < 0.05; ^##^ *p* <0.01; ^###^ *p* < 0.001 vs. vehicle-treated CDDP group. CDDP, cisplatin; FPN, ferroportin; DMT1, Divalent metal transporter-1; FIH, factor-inhibiting HIF. Model: vehicle-treated CDDP group.

It has been well demonstrated that the mammalian target of rapamycin (mTOR) pathway modulates HIF-1α expression *via* different external stimuli (Sudhagar et al., 2011). TBN can scavenge free radicals and increase AMPK expression and decrease mTOR expression (Supplementary Figure S2; Figures 5I–K). This had also been verified in HK-2 cells, shown in Supplementary Figure S3 and Supplementary Figure S4. In HepG2 cells, we used 4E1rcat (10 μM), which was the direct inhibitor of 4EBP1, to examine whether TBN can still cause changes in HIF-1α and HIF-2α levels. As the results shown (Figures 5L–O), 4E1rcat decreased HIFs and TBN reversed the down-regulation of HIFs, which demonstrated that the effects of TBN on HIFs is *via* the mTOR/4E-BP1 pathway. Hence, based on these results, we examined whether or not the phosphorylation of mTOR impacted the effects of TBN in CDDP-induced C57BL/6J mice. Compared to the normal mice, the expression of the phosphorylation status of mTOR (Ser2448) significantly increased (approximately 10%–20%) in CDDP-CKD mice in both liver and kidney, while TBN dose-dependently reversed the increase in p-mTOR in the kidney (Figures 6A–D).

TBN reduced the expressions of PHD3 and FIH (Figures 6A,B,E–H), which regulates two different degradation pathways separately. One degradation pathway is through prolyl hydroxylase, which hydroxylates of HIF-α leading to the recognition of HIF-α by Von Hippel−Lindau protein (pVHL) and subsequent degradation *via* the ubiquitin-proteasome system. Another degradation pathway is through inhibition of FIH, which prevents HIF-α from associating with the coactivator p300/CBP, resulting in a reduction of HIF-mediated transcription. The precise mechanism of action of how TBN regulates the expression levels of PHD3 and FIH is not understood. We speculated that TBN does not activate HIF by directly acting on PHD and FIH since previous studies had demonstrated that TBN acts by scavenging ROS (Wu et al., 2019; Jing et al., 2021). We demonstrated that TBN scavenged ROS in CoCl_2_-induced Hep3B and CoCl_2_-induced HepG2 cells (Supplementary Figure S2).

TBN inhibited hepcidin in CDDP-induced C57BL/6J mice and improved serum iron and Tf levels in STZ-induced SHR rats, which suggested that TBN may have effects on iron stores and metabolism. It was reported that stabilization of HIFs leads to regulation of EPO biosynthesis and iron homeostasis. Thus, we determined whether TBN treatment impacted iron absorption and transportation from kidney and liver to plasma *via* western blot; specifically, we examined the levels of ferritin, FPN, and DMT1 in the liver and kidney in CDDP-induced C57BL/6J mice. The results in Figures 6A,B,I–N showed that treatment with TBN significantly increased FPN levels in both organs, while DMT1 and ferritin protein levels decreased with TBN treatment when compared to the vehicle-treated CDDP group. These results demonstrated that TBN can impact iron homeostasis and regulate HIFs to improve anemia.

## Discussion

Approximate 40% of the people with diabetes ultimately develop DKD, of which may further progress into ESRD (Reddy et al., 2013). The clinical manifestations of DKD include progressive decline in the glomerular filtration rate, microalbuminuria, glomerular hypertrophy and thickening of the glomerular basement membrane, glomerular sclerosis and interstitial fibrosis. Eventually, these effects result in renal failure, requiring either dialysis or renal replacement therapy. To date, there are only a few promising options available for the treatment of DKD in clinical practice.

Many studies have found that hypertension is an important risk factor in the progression of chronic renal failure, as hypertension exacerbates diabetic kidney damage in diabetic patients (James et al., 2015). Thus, in this study, the DKD rat model induced by STZ in SHR rats was chosen. The coexistence of diabetes and hypertension exuberates renal function, increases albuminuria, and promotes the progression of DKD. The symptoms and pathological characteristics of DKD in SHR rats resemble that of DKD in patients (Elmarakby et al., 2012). Our results shown that TBN decreased the level of urea nitrogen and ameliorated kidney injury (Figure 1E), suggesting that TBN was a potential DKD treatment.

Anemia often appears earlier in DKD than in other types of CKD. EPO is important for RBC production. Thus, patients with diabetes are vulnerable to EPO deficiency and are twice as likely to have anemia as compared with those non-diabetic CKD patients (Thomas et al., 2006). Compared to non-diabetic patients, anemia in those with renal insufficiency occurs earlier and more seriously (Wang et al., 1995; Haase, 2010; Suzuki and Yamamoto, 2016). More recently, it has been shown that disordered iron homeostasis is another vital factor resulting in renal anemia (Panwar and Gutiérrez, 2016). As shown in our results, TBN was effective at promoting EPO synthesis in STZ-induced DKD in SHR rats. TBN also improved iron levels and enhances the function of iron by increasing Tf expression and suppressing hepcidin expression. These results demonstrated that TBN significantly improved renal anemia (Figures 1F–I). On the other hand, HIF-2α is sensitive to oxygen levels and cellular iron (Mastrogiannaki et al., 2009; Shah et al., 2009). HIF-2α plays an essential role in the absorption of iron during systemic iron deficiency (Anderson et al., 2011; Taylor et al., 2011; Ramakrishnan et al., 2015). Immunohistochemistry (IHC) and western blot results demonstrated that TBN increases expression of HIF-2α and HO-1. The effects of TBN to ameliorate renal anemia in DKD rats were due to their ability to scavenge free radicals, reduce oxidative stress, and activate the AMPK/mTOR/HIF pathway (Figure 3C–E, 5I–K; Supplementary Figures S2, S3).

Previous studies have shown that 15 mg/kg of CDDP caused CKD characterized by a sustained reduction in eGFR and reduced kidney mass. Two weeks after the second dose of CDDP, both of these features of CKD are typically present, indicating that the disease model has been established (Landau et al., 2019). In this CDDP-induced model, TBN effectively relieved the symptoms of anemia and increased the levels of the RBC count, HCT, Hb and EPO (Figures 2C–E,H). TBN also increased stabilization of HIF-1α and HIF-2α and the transcriptional activation of the EPO gene in hypoxic conditions (Figures 4A–F,I–L). The down-regulation of the expression of hepcidin in the serum treated with TBN was not dose-dependent. We thought that this result may be an artefact due to coincidental individual differences in the mice. These findings shown that the increased synthesis and stable expression of HIFs can effectively improve the expression of EPO and the decline of blood cell function caused by anemia. Furthermore, TBN treatment rapidly increased HIF-1α and HIF-2α synthesis both in C57BL/6J mice and hepatoma cells. TBN also affected the stability of HIF-1α and the transcription of its mRNA. TBN significantly blocked the degradation pathway of HIFs by inhibiting the expression of the PHD3 and FIH proteins (Figures 6A,B,E–H).

In renal anemia, iron is secluded in macrophages, intestinal iron absorption is diminished, and erythropoiesis is impaired by the low amounts of iron (Weiss and Goodnough, 2005; Goodnough et al., 2010; Ganz, 2011). Iron cannot be directly absorbed by the body; rather, a unique transportation pathway is required for iron to be absorbed into the blood, and it needs to be delivered to the bloodstream in a soluble form for use by cells and tissues. Thus, the regulation of the iron metabolism is important for the maintenance of a healthy physiological state of the human body. Our results found that TBN enhanced the FPN levels in the livers of mice with renal anemia. Through binding to FPN, hepcidin induces its internalization, therefore limiting iron export (Bârsan et al., 2015). Ferritin is an acute phase product and, as such, is frequently elevated in patients with CKD. TBN can reverse this increase in CKD (Figures 6A,B,I,J,L,M). High levels of dietary iron produce an increase in DMT1 expression in hepatocytes, promoting iron acquisition, whereas low levels decrease hepatic DMT1 expression, causing a reduction in iron accumulation (Oates et al., 2000; Trinder et al., 2000). Higher ROS levels are present in DMT1-mutant erythrocytes (Zidova et al., 2014). The loss of DMT1 is suggested to protect against oxidative damage to the pancreas and help to maintain insulin sensitivity despite iron overload (Jia et al., 2013). The anti-oxidative stress properties of TBN caused DMT1 inhibition, as DMT1 caused oxidative damage and ROS accumulation (Figures 6K,N).

**FIGURE 7:**
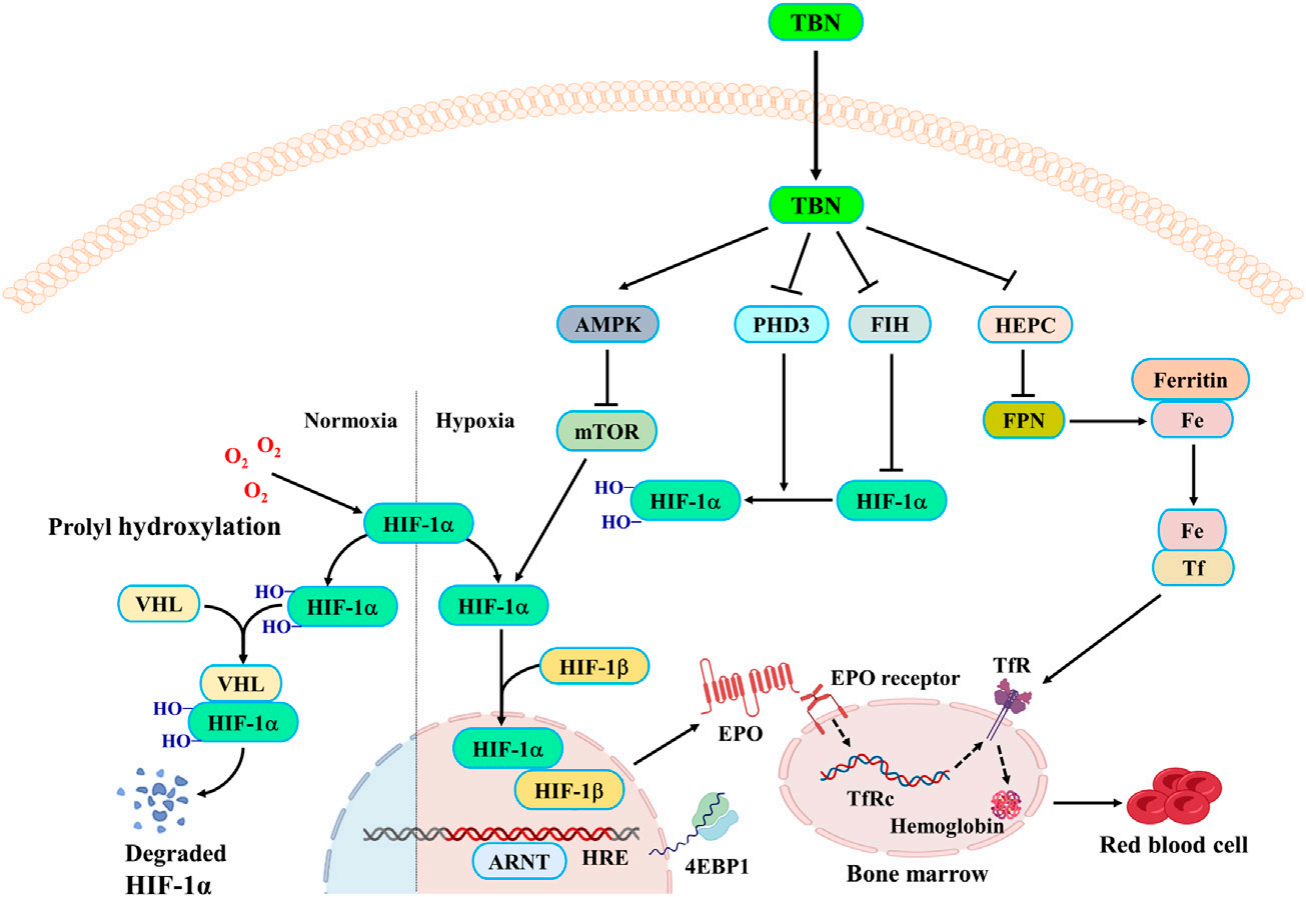
TBN mechanism of action in anemia.

Recently, researchers find that mTORC1 regulates HIF-1α protein accumulation *via* promoting the transcription of HIF-1α mRNA, which is blocked by either knockdown or inhibition of the STAT3 (Luo et al., 2021). Furthermore, studies find that STAT3 is directly phosphorylated by mTORC1 on Ser727 under hypoxic conditions, thus promoting HIF-1α mRNA transcription. mTORC1 regulates HIF-1α synthesis on a translational level through co-operative regulation of both ribosomal protein S6K1 and initiation factor 4E-BP1, while HIF-1α degradation is not affected (Jia et al., 2013). Under hypoxic conditions, TBN promoted the stable expression of HIF-1α and HIF-2α in the nucleus by promoting the expression of HIF-1α and HIF-2α (Figures 4G,H). The combination of HIF-1α and HIF-1α forms aggregate and further promotes the transcription and synthesis of EPO. TBN improved the synthesis rate of HIF-1α (Figures 5G,H). On the other hand, TBN can positively regulate the AMPK/mTOR/4EBP1 pathway. However, there is no corresponding experimental data to support whether there is a direct correlation between the effects of TBN on HIFs and mTOR. The molecular mechanism by which TBN regulates HIFs may be multi-faceted (Figure 7). It increases the expression of protein, the synthesis rate and reduces the degradation of HIFs. TBN was most likely responsible for promoting HIF transcription and translation by activating the AMPK pathway. Therefore, exploring the molecular mechanisms of TBN on HIFs in more detail is needed (Sudhagar et al., 2011).

## Data Availability

The original contributions presented in the study are included in the article/Supplementary Materials, further inquiries can be directed to the corresponding authors.
