# Supplementary material for: Tetramethylpyrazine nitrone activates hypoxia-inducible factor and regulates iron homeostasis to improve renal anemia
Source: Front Pharmacol. 2022 Oct 17;13:964234. doi: 10.3389/fphar.2022.964234 (PMC9618660; doi:10.3389/fphar.2022.964234)
Supplement: Supplementary file 1 [file DataSheet2.PDF]

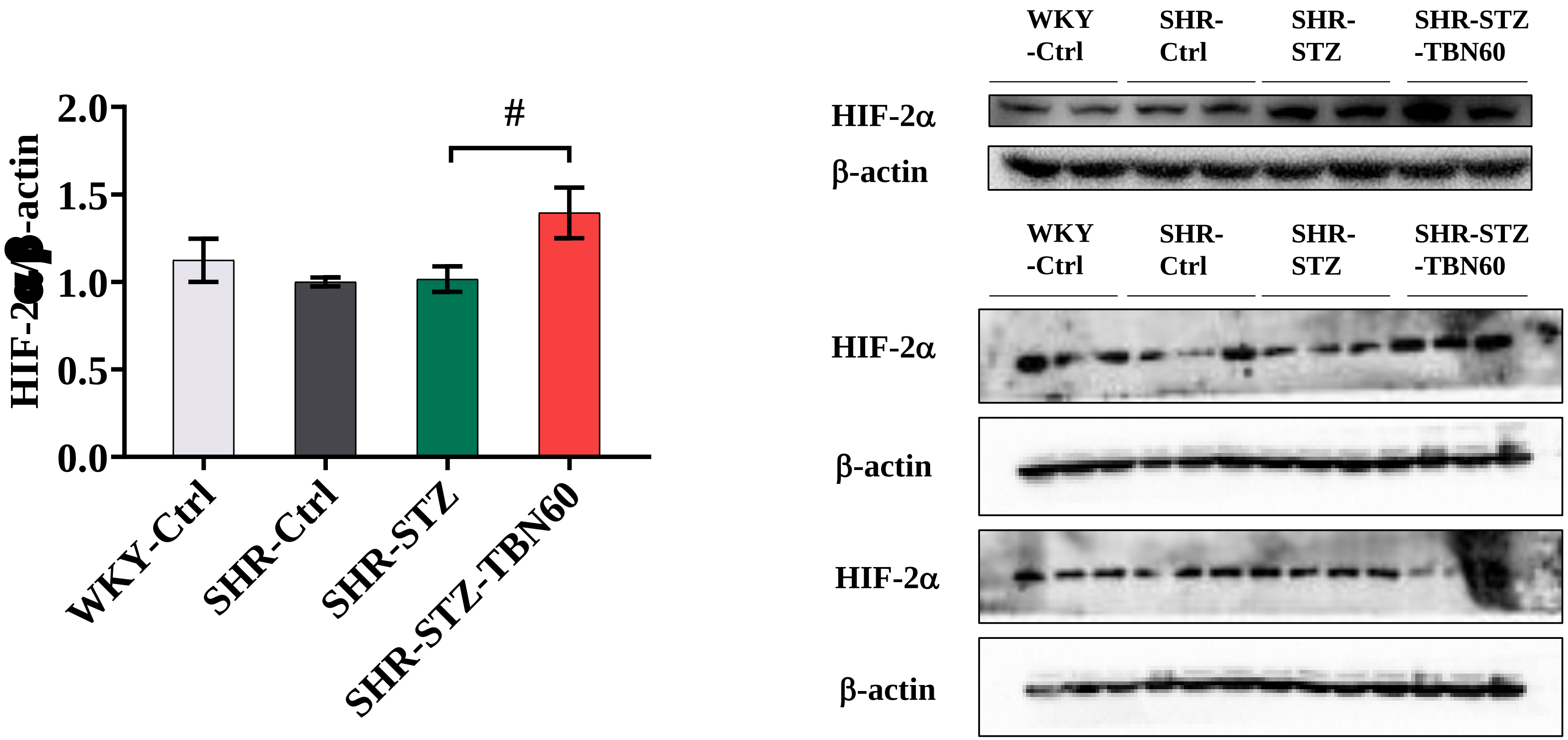
HIF-2α/β-actin
2.0
1.5
1.0
0.5
0.0
#
WKY-Ctrl
SHR-Ctrl
SHR-STZ
SHR-STZ-TBN60
WKY -Ctrl
SHR- Ctrl
SHR- STZ
SHR-STZ -TBN60
HIF-2α
β-actin
WKY -Ctrl
SHR- Ctrl
SHR- STZ
SHR-STZ -TBN60
HIF-2α
β-actin
HIF-2α
β-actin

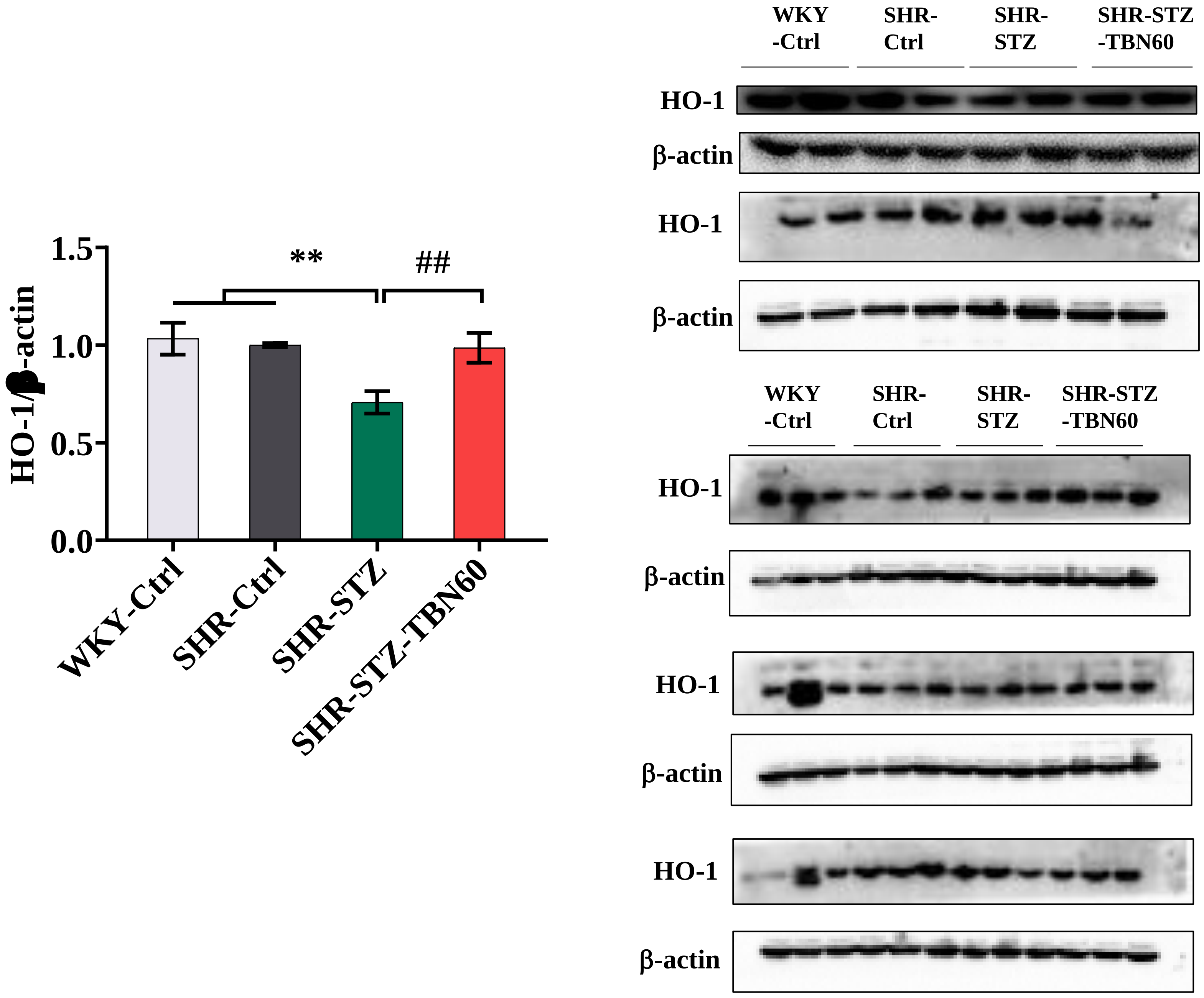

HO-1/β-actin
1.5
1.0
0.5
0.0
**
##
WKY-Ctrl
SHR-Ctrl
SHR-STZ
SHR-STZ-TBN60
WKY -Ctrl
SHR- Ctrl
SHR- STZ
SHR-STZ -TBN60
HO-1
β-actin
HO-1
β-actin
WKY -Ctrl
SHR- Ctrl
SHR- STZ
SHR-STZ -TBN60
HO-1
β-actin
HO-1
β-actin
HO-1
β-actin

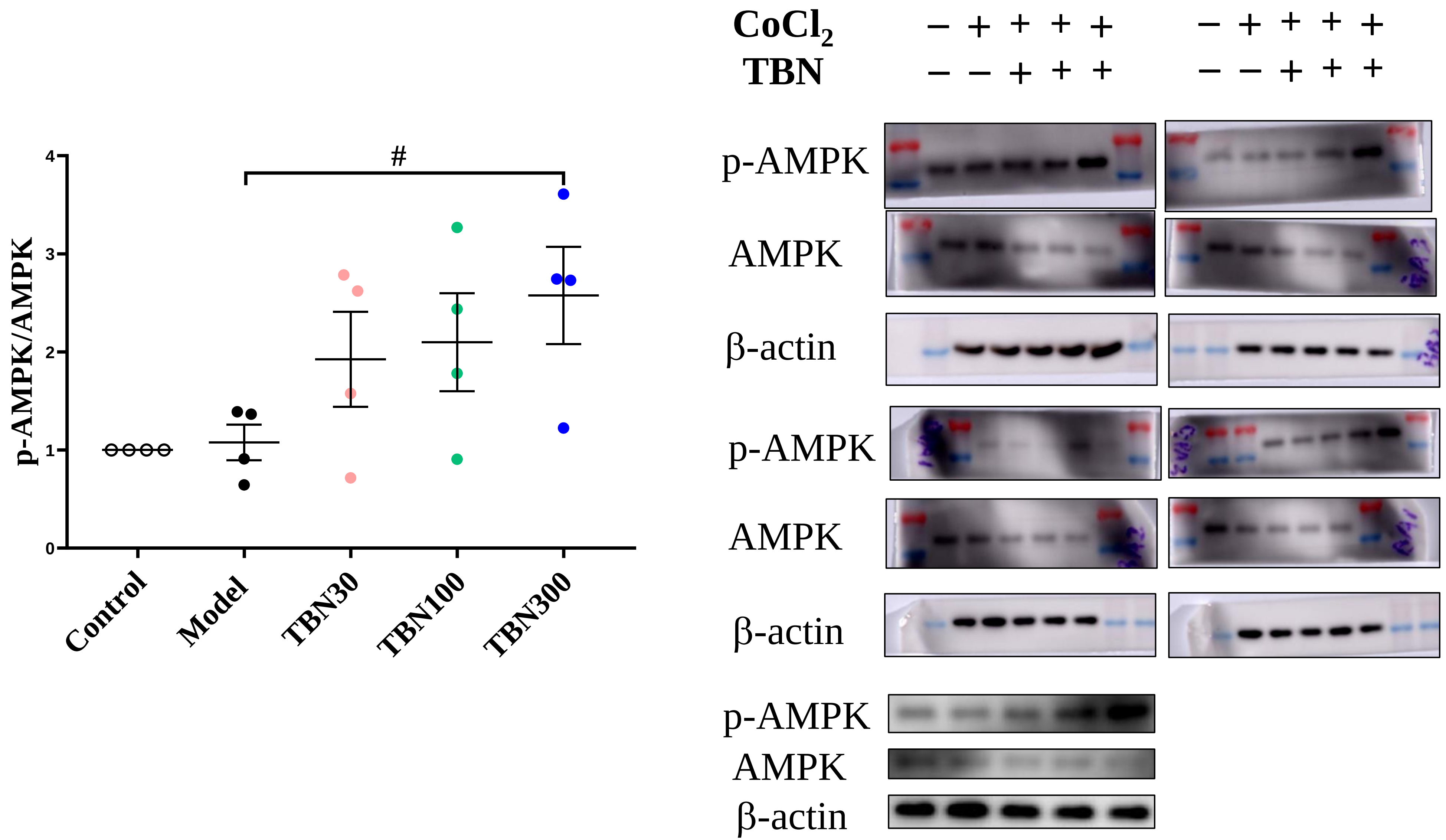

p-AMPK/AMPK
4
3
2
1
0
#
Control
Model
TBN30
TBN100
TBN300
CoCl2
TBN
p-AMPK
AMPK
β-actin
p-AMPK
AMPK
β-actin
p-AMPK
AMPK
β-actin

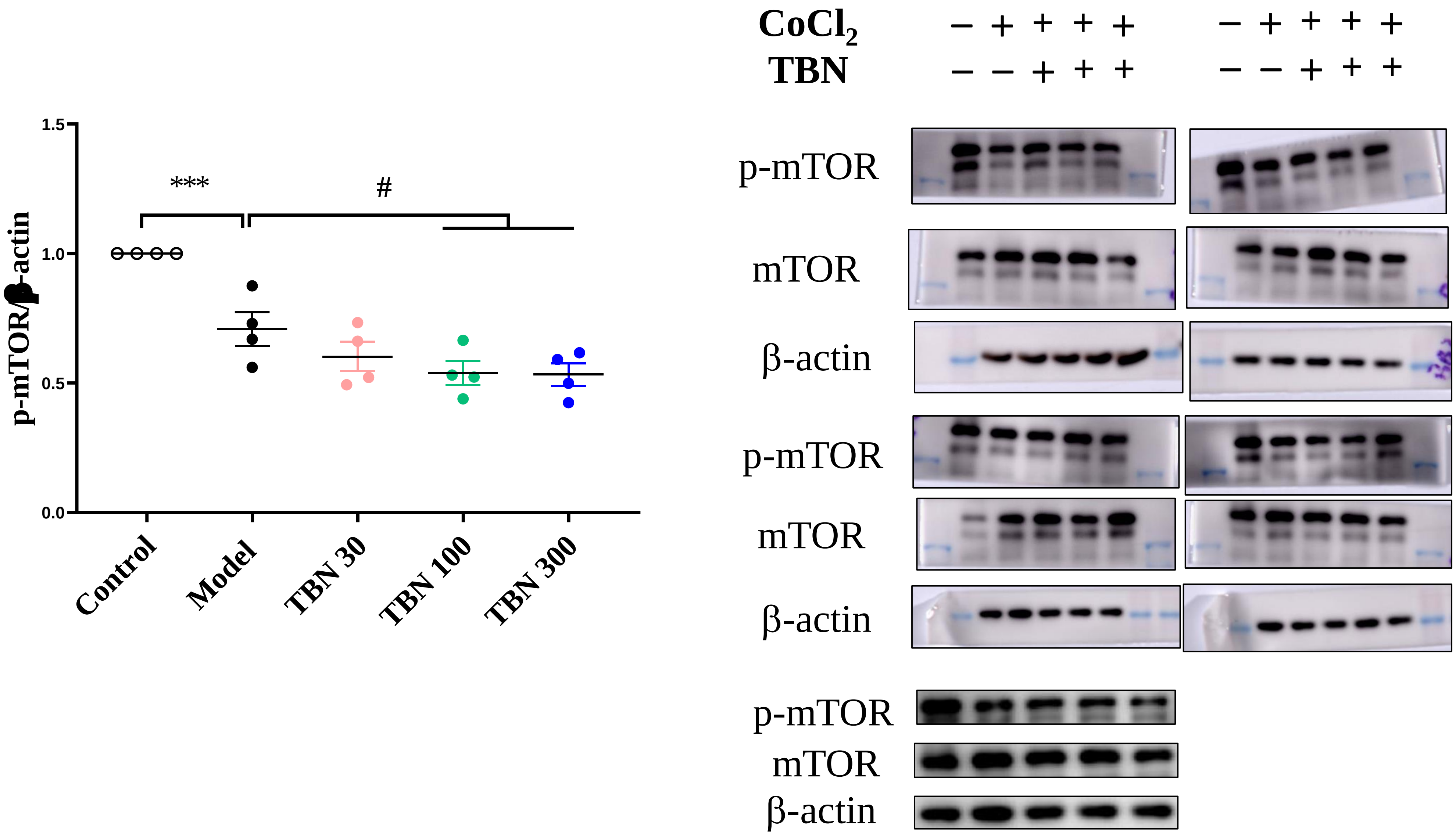

p-mTOR/β-actin
1.5
1.0
0.5
0.0
***
#
Control
Model
TBN 30
TBN 100
TBN 300
CoCl2
TBN
− + + + +
− − + + +
− + + + +
− − + + +
p-mTOR
mTOR
β-actin
p-mTOR
mTOR
β-actin
p-mTOR
mTOR
β-actin

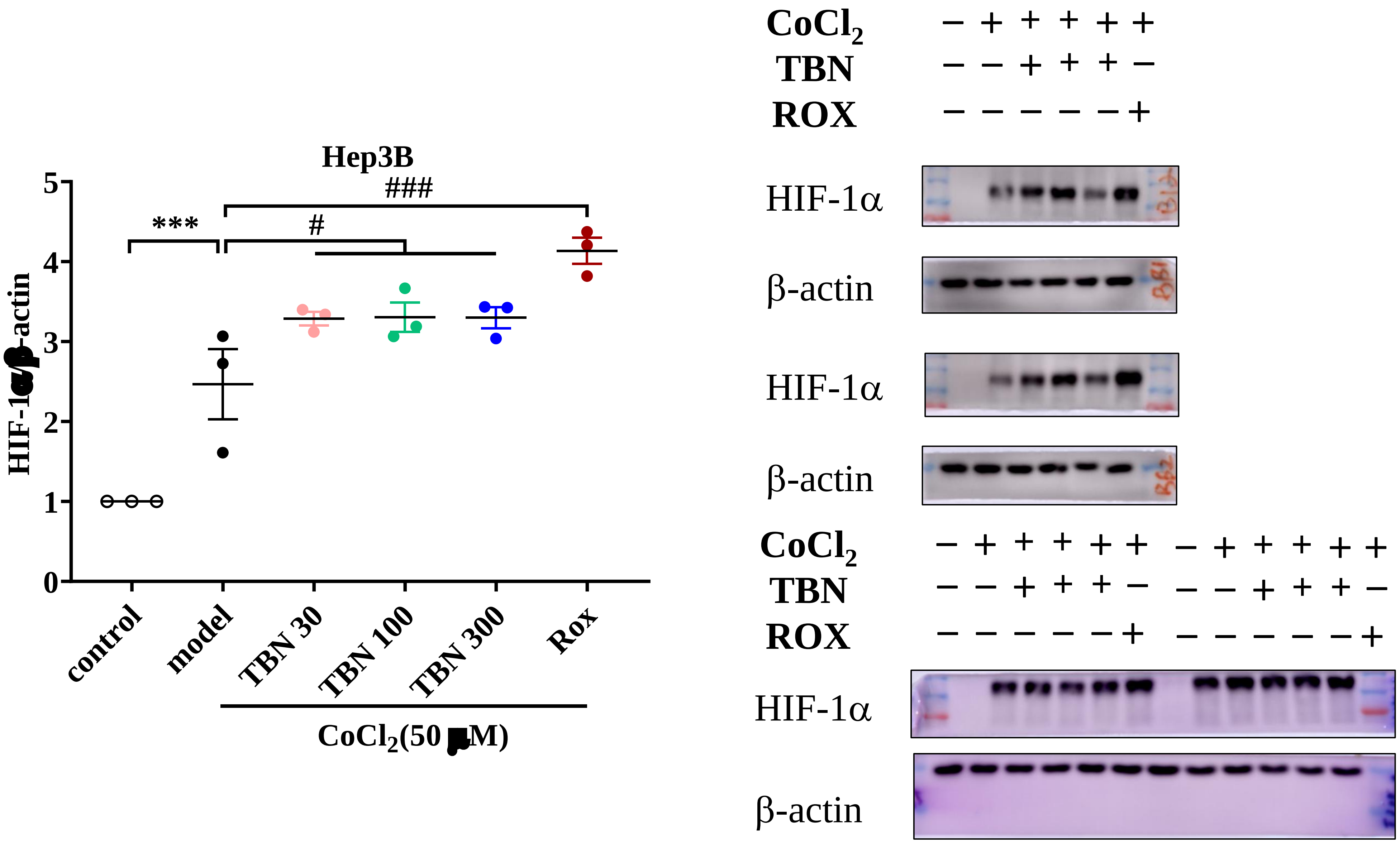

Hep3B
HIF-1α/β-actin
0
1
2
3
4
5
***
#
###
control
model
TBN 30
TBN 100
TBN 300
Rox
CoCl2(50 μM)
CoCl2 − + + + + +
TBN − − + + + −
ROX − − − − − +
HIF-1α
β-actin
HIF-1α
β-actin
CoCl2 − + + + + + − + + + + +
TBN − − + + + − − − + + + −
ROX − − − − − + − − − − − +
HIF-1α
β-actin

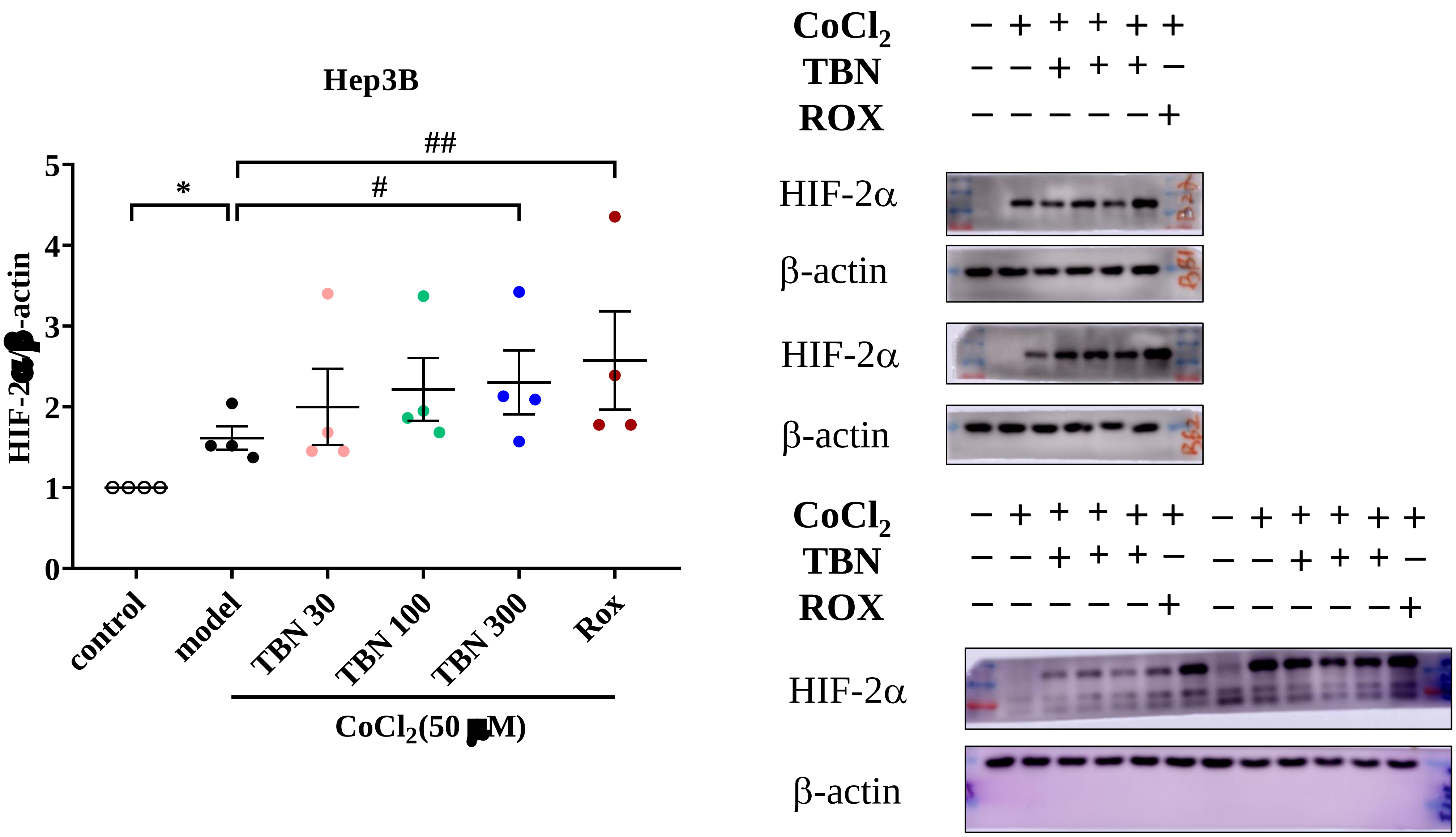

Hep3B
HIF-2α/β-actin
0
1
2
3
4
5
*
#
##
control
model
TBN 30
TBN 100
TBN 300
Rox
CoCl2(50 μM)
CoCl2
TBN
ROX
HIF-2α
β-actin
HIF-2α
β-actin
CoCl2
TBN
ROX
HIF-2α
β-actin

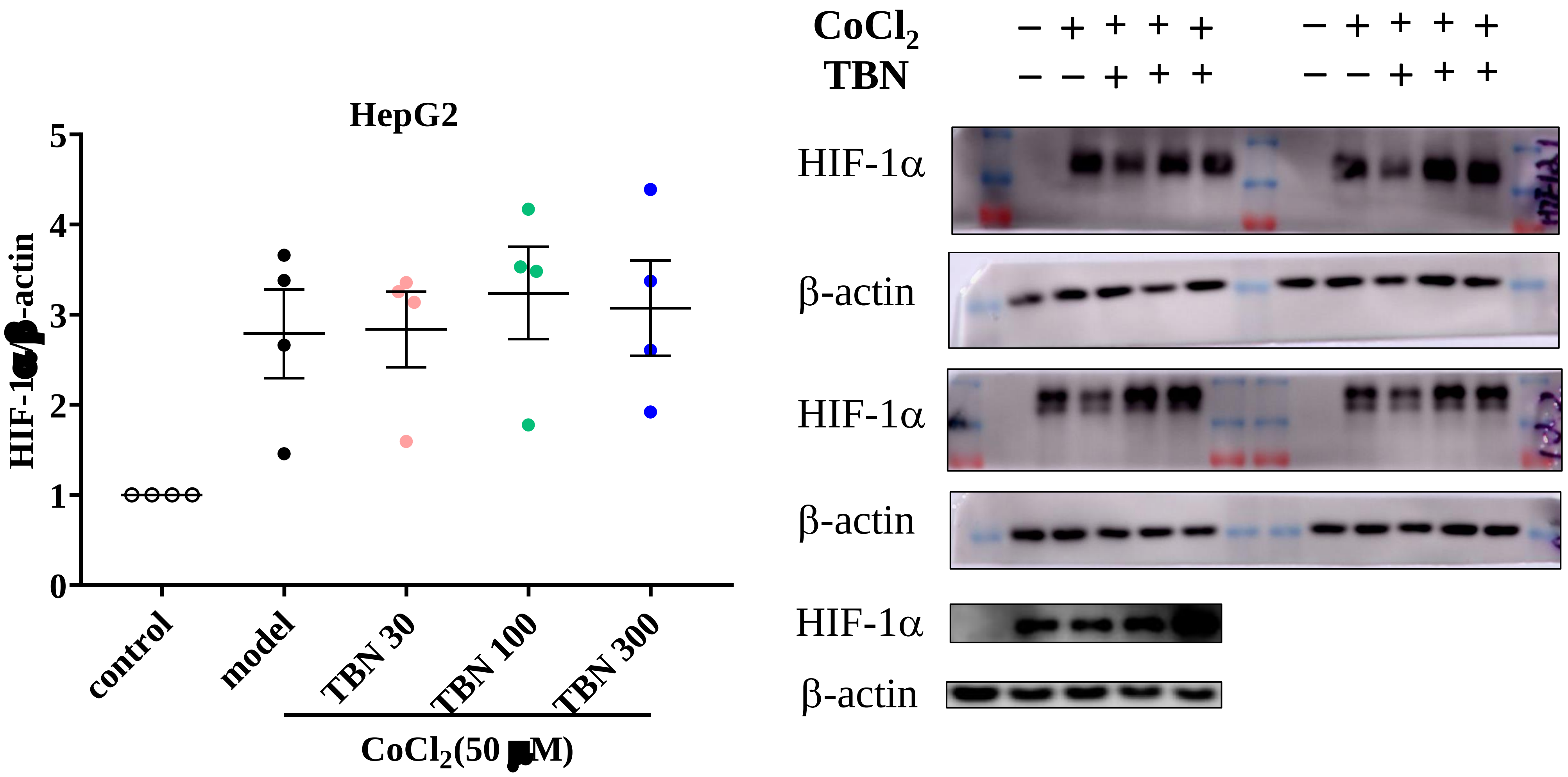

HepG2
HIF-1α/β-actin
5
4
3
2
1
0
control
model
TBN 30
TBN 100
TBN 300
CoCl2(50 μM)
CoCl2
TBN
− + + + + − + + + +
− − + + + − − + + +
HIF-1α
β-actin
HIF-1α
β-actin
HIF-1α
β-actin

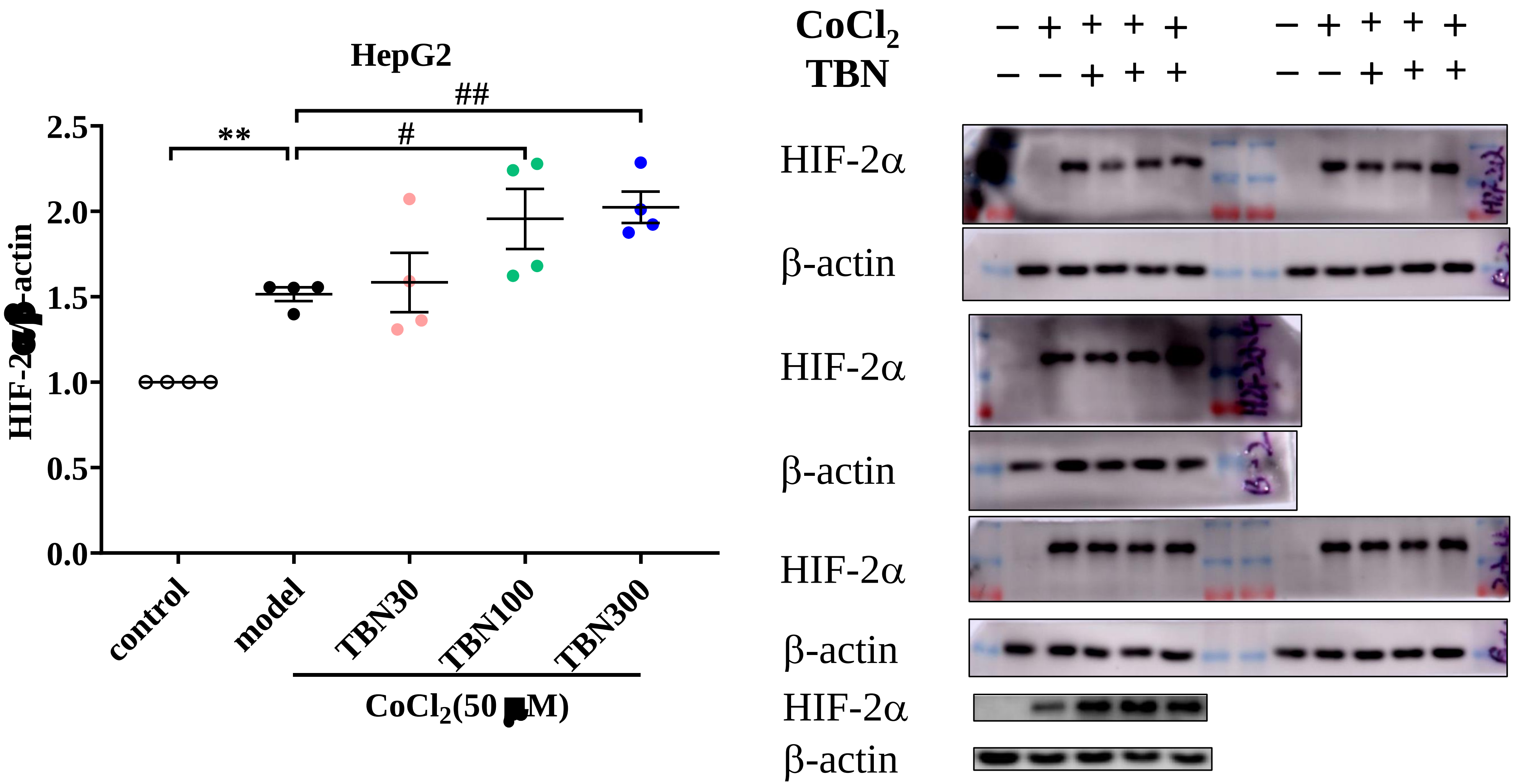
HepG2
HIF-2α/β-actin
2.5
2.0
1.5
1.0
0.5
0.0
**
#
##
control
model
TBN30
TBN100
TBN300
CoCl2(50 μM)
CoCl2
TBN
HIF-2α
β-actin
HIF-2α
β-actin
HIF-2α
β-actin
HIF-2α
β-actin

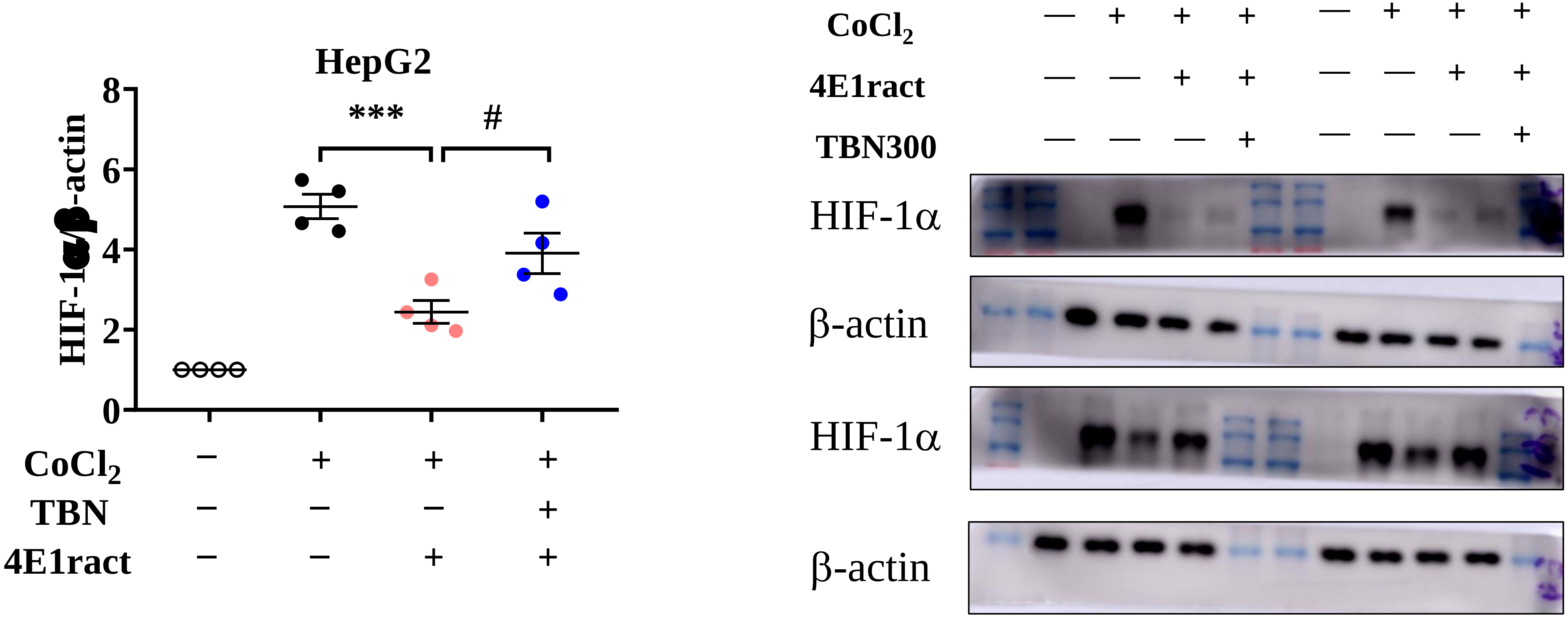
HepG2
HIF-1α/β-actin
8
6
4
2
0
***
#
CoCl2 − + + +
TBN − − − +
4E1ract − − + +
CoCl2 — + + + — + + +
4E1ract — — + + — — + +
TBN300 — — — + — — — +
HIF-1α
β-actin
HIF-1α
β-actin

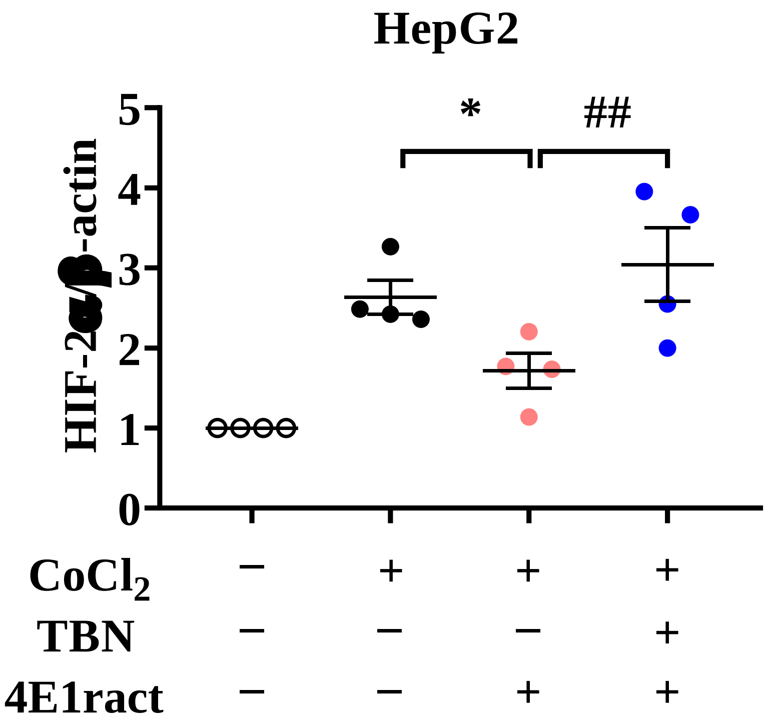
HepG2
HIF-2α/β-actin
5
4
3
2
1
0
*
##
CoCl2
TBN
4E1ract

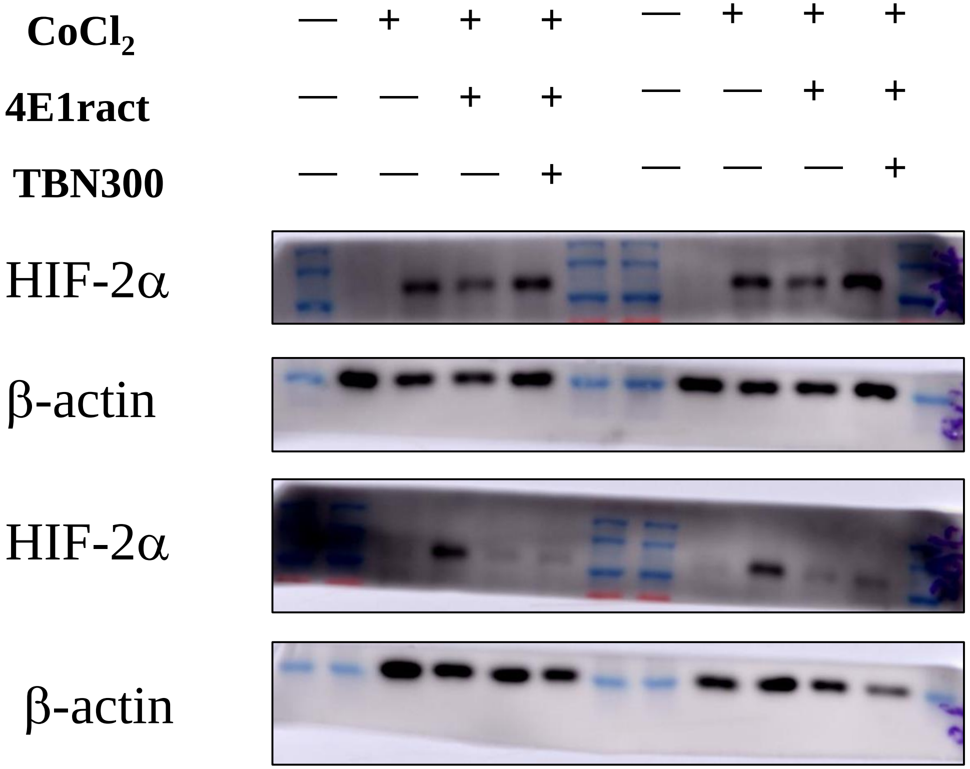
CoCl2
4E1ract
TBN300
HIF-2α
β-actin
HIF-2α
β-actin

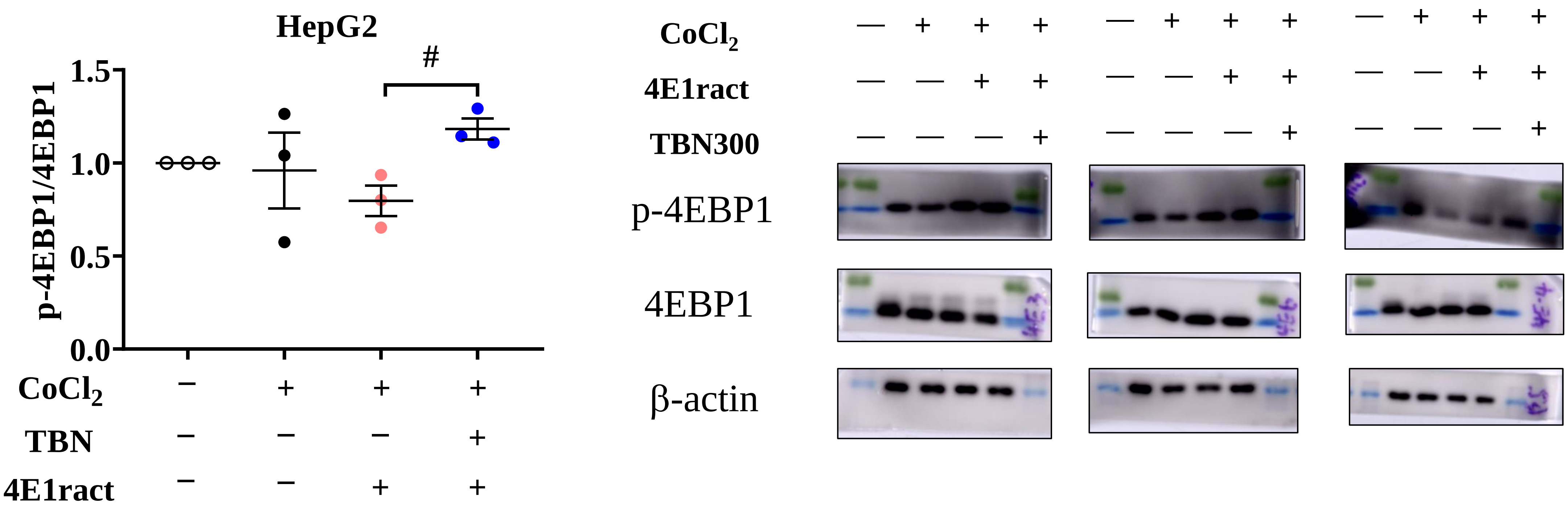

HepG2
#
p-4EBP1/4EBP1
1.5
1.0
0.5
0.0
CoCl2
TBN
4E1ract
CoCl2
4E1ract
TBN300
p-4EBP1
4EBP1
β-actin

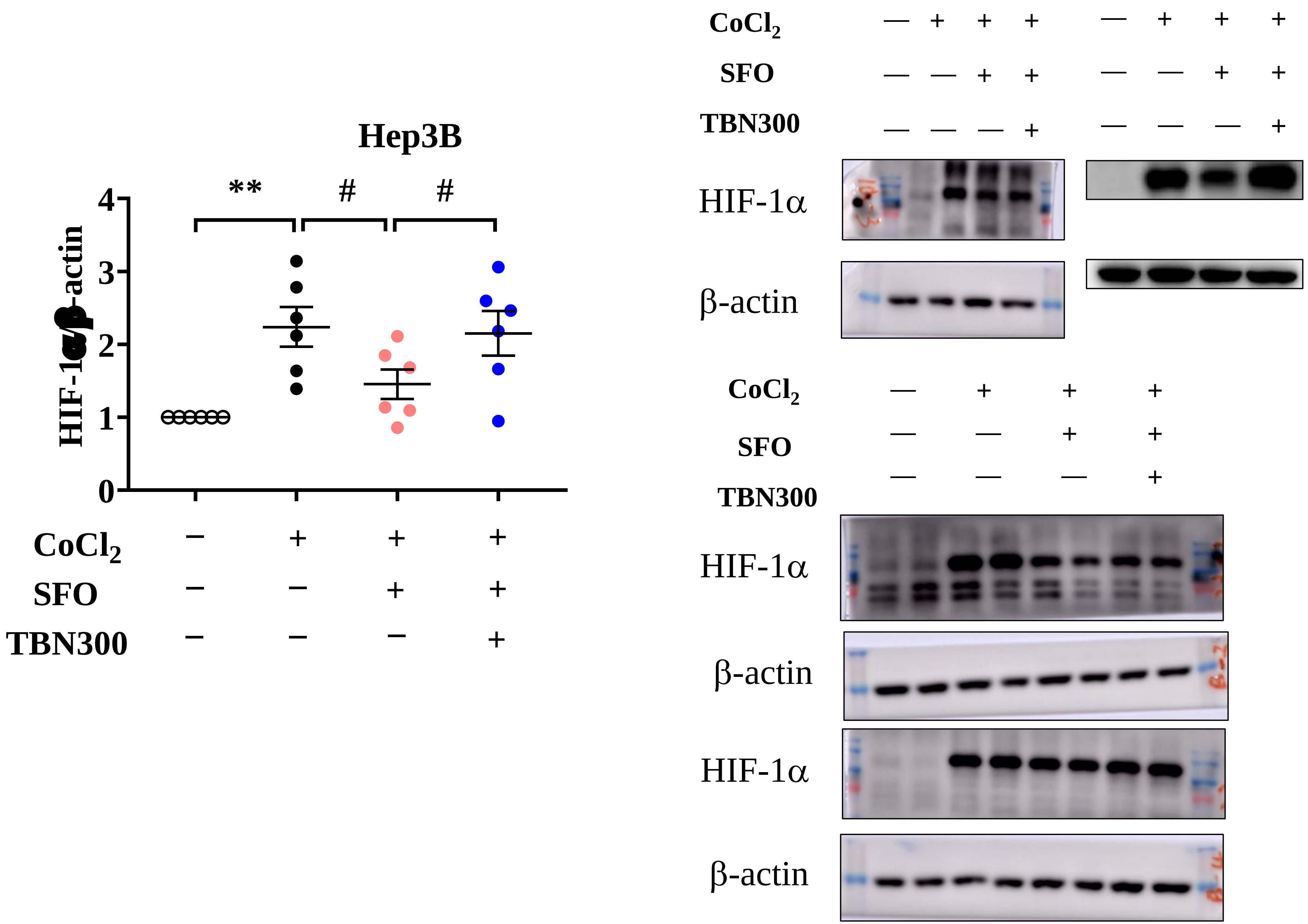

Hep3B
HIF-1α/β-actin
4
3
2
1
0
**
#
#
CoCl2
SFO
TBN300
HIF-1α
β-actin
HIF-1α
β-actin
HIF-1α
β-actin

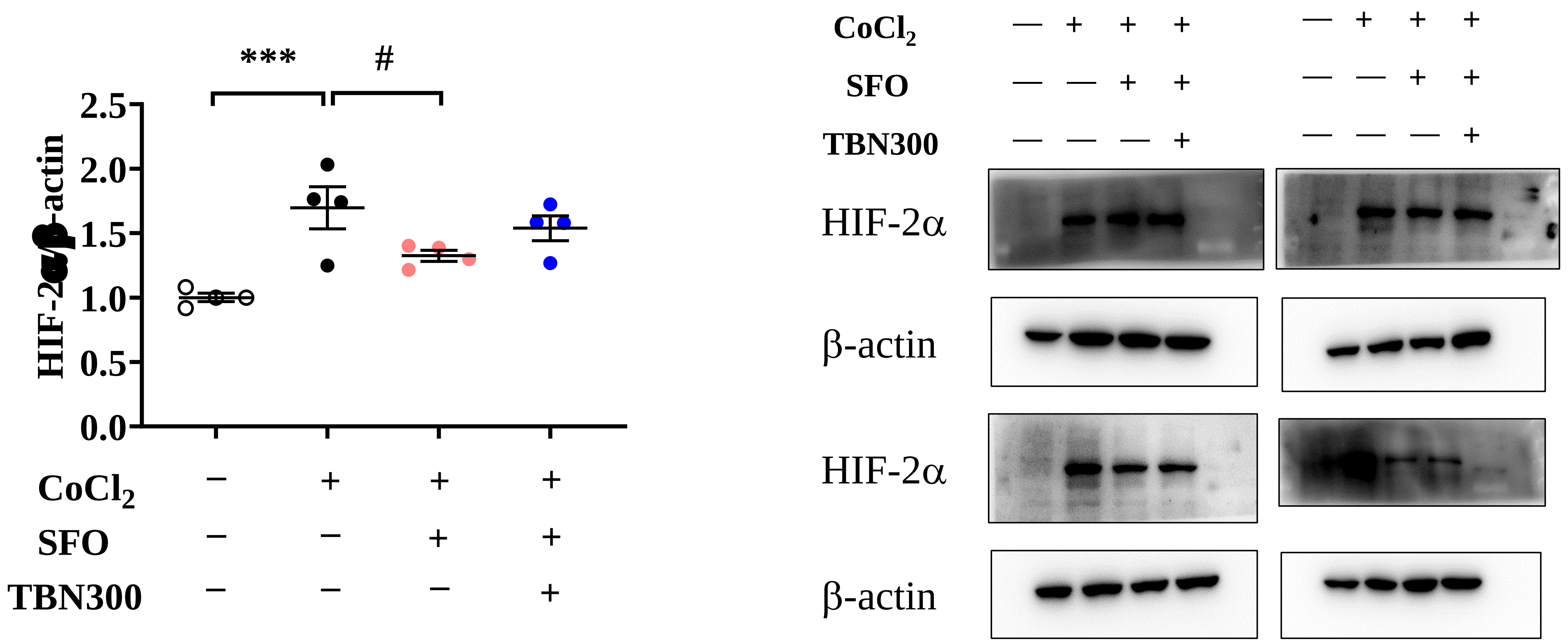
***
#
HIF-2α/β-actin
2.5
2.0
1.5
1.0
0.5
0.0
CoCl2
SFO
TBN300
HIF-2α
β-actin
HIF-2α
β-actin

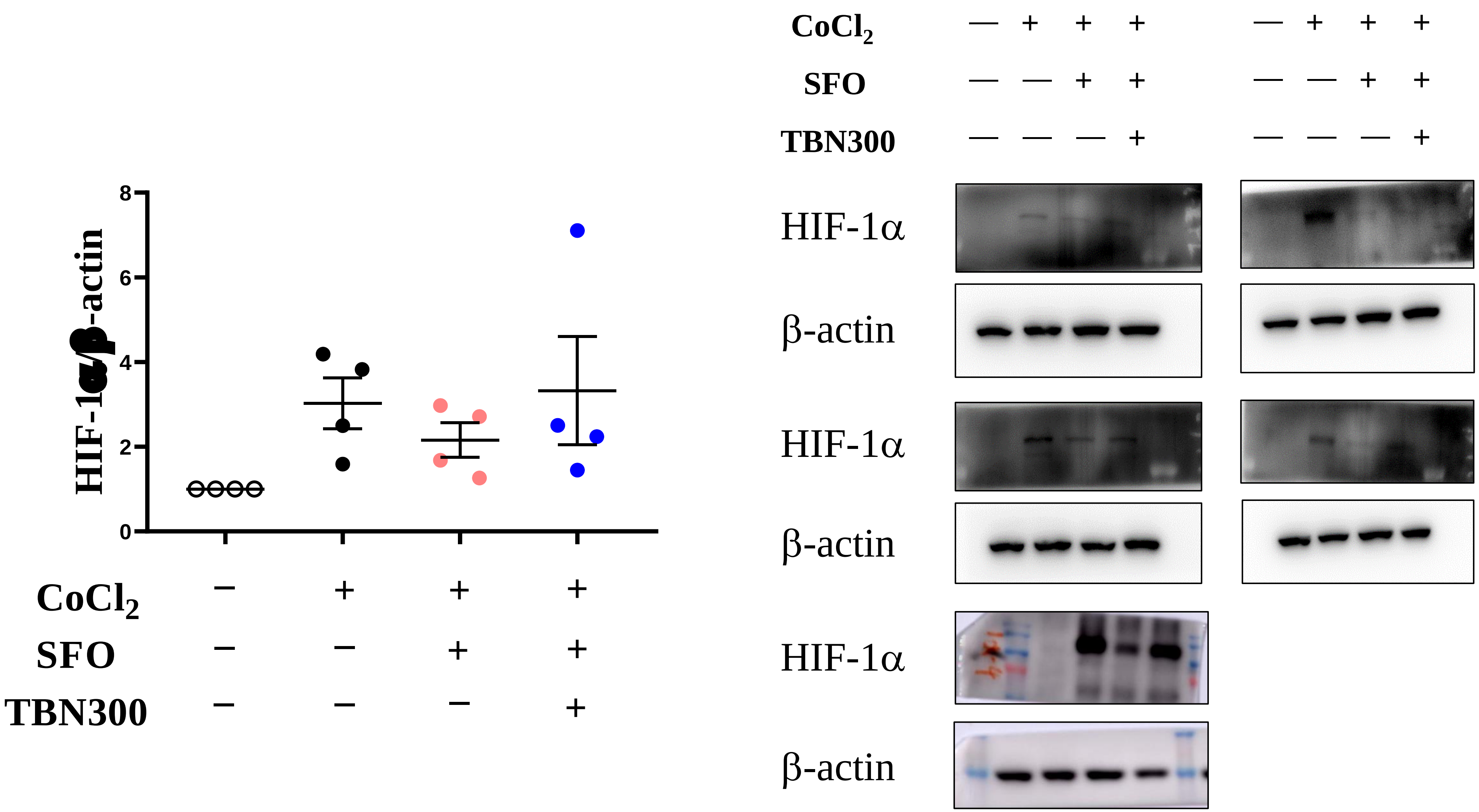
HIF-1α/β-actin
0
2
4
6
8
CoCl2
SFO
TBN300
HIF-1α
β-actin
HIF-1α
β-actin
HIF-1α
β-actin

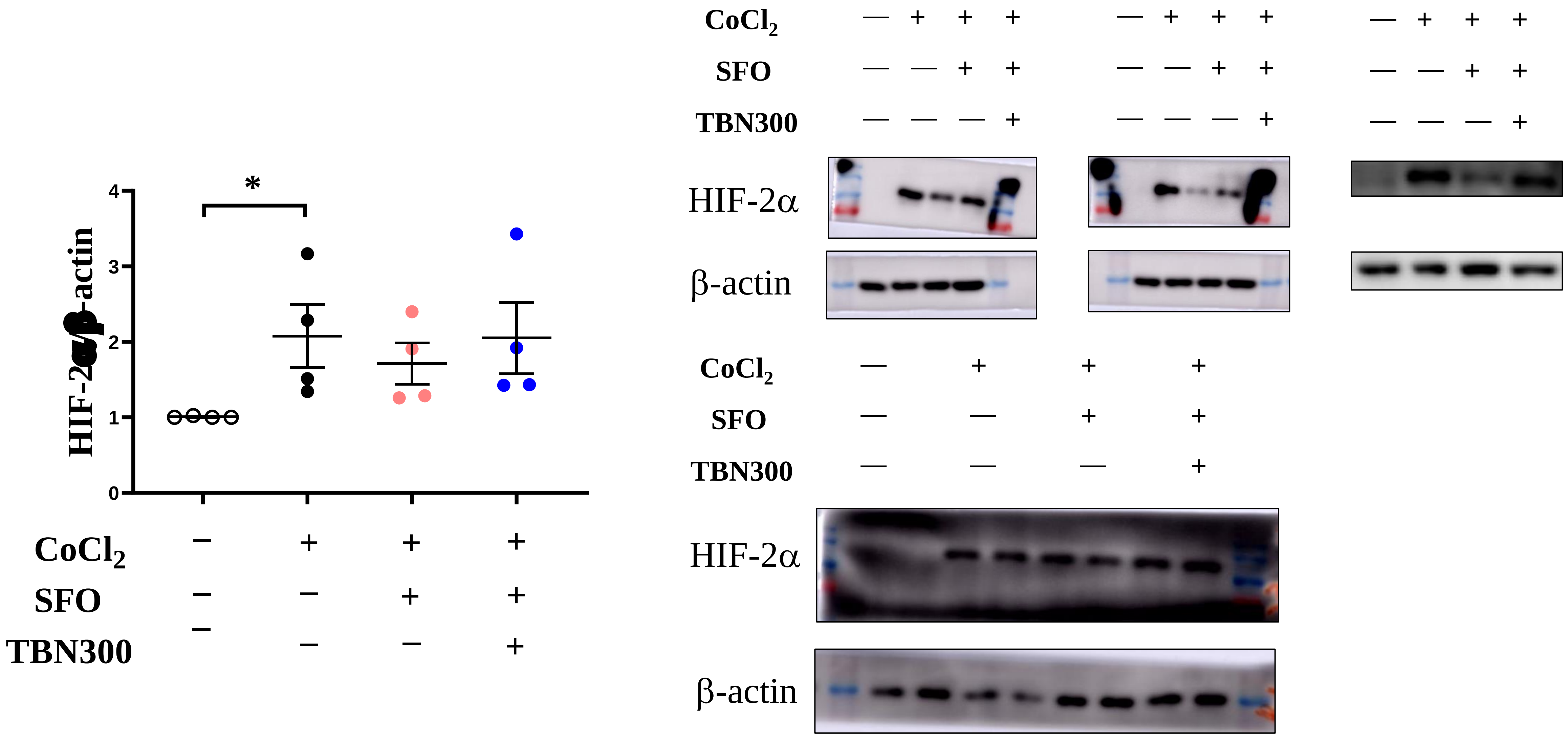
*
4
3
2
1
0
HIF-2α/β-actin
CoCl2
SFO
TBN300
HIF-2α
β-actin
CoCl2
SFO
TBN300
HIF-2α
β-actin

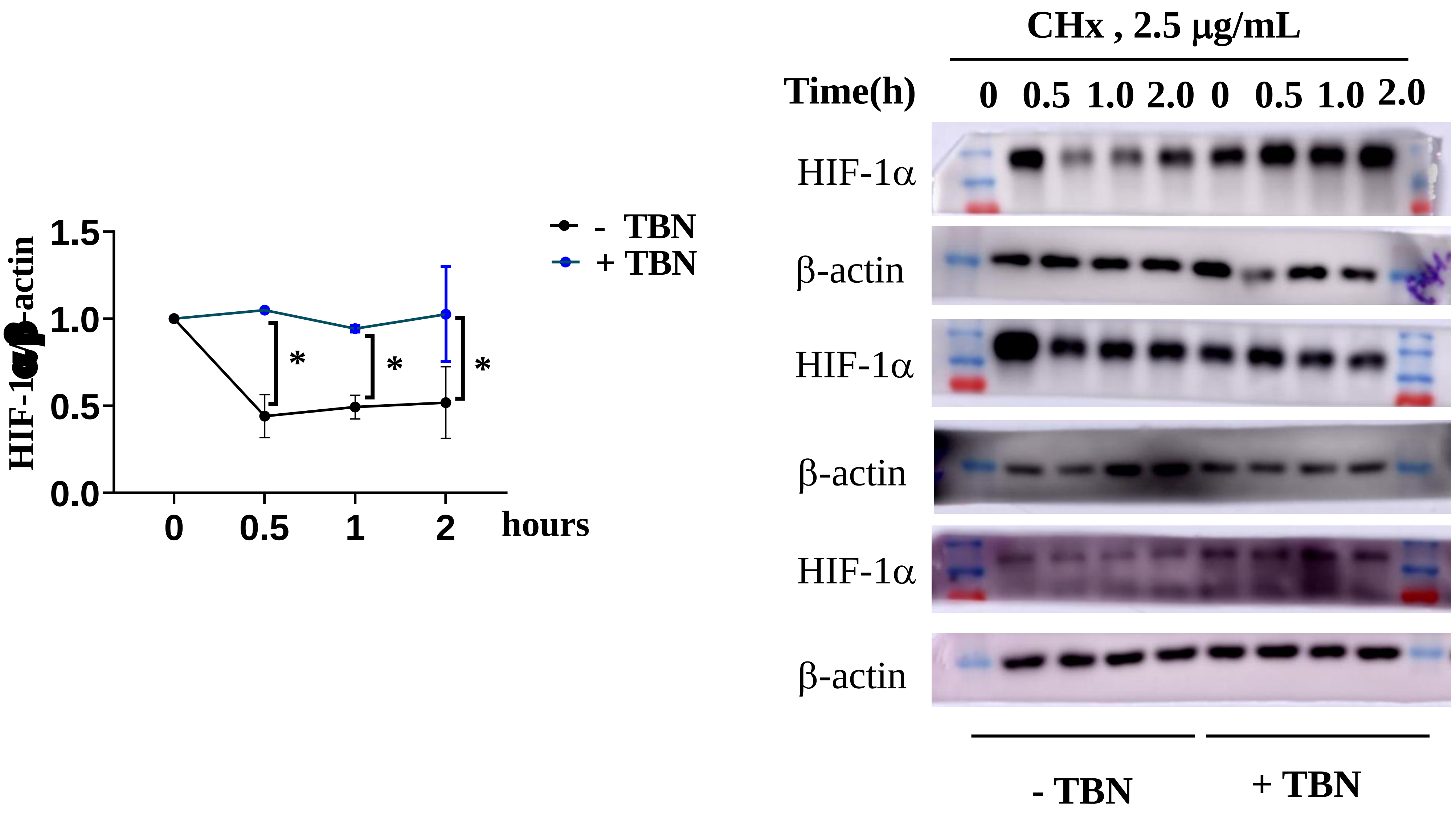

HIF-1α/β-actin
1.5
1.0
0.5
0.0
0
0.5
1
2
hours
*
*
*
- TBN
+ TBN
CHx , 2.5 μg/mL
Time(h)
0 0.5 1.0 2.0 0 0.5 1.0 2.0
HIF-1α
β-actin
HIF-1α
β-actin
HIF-1α
β-actin
- TBN
+ TBN

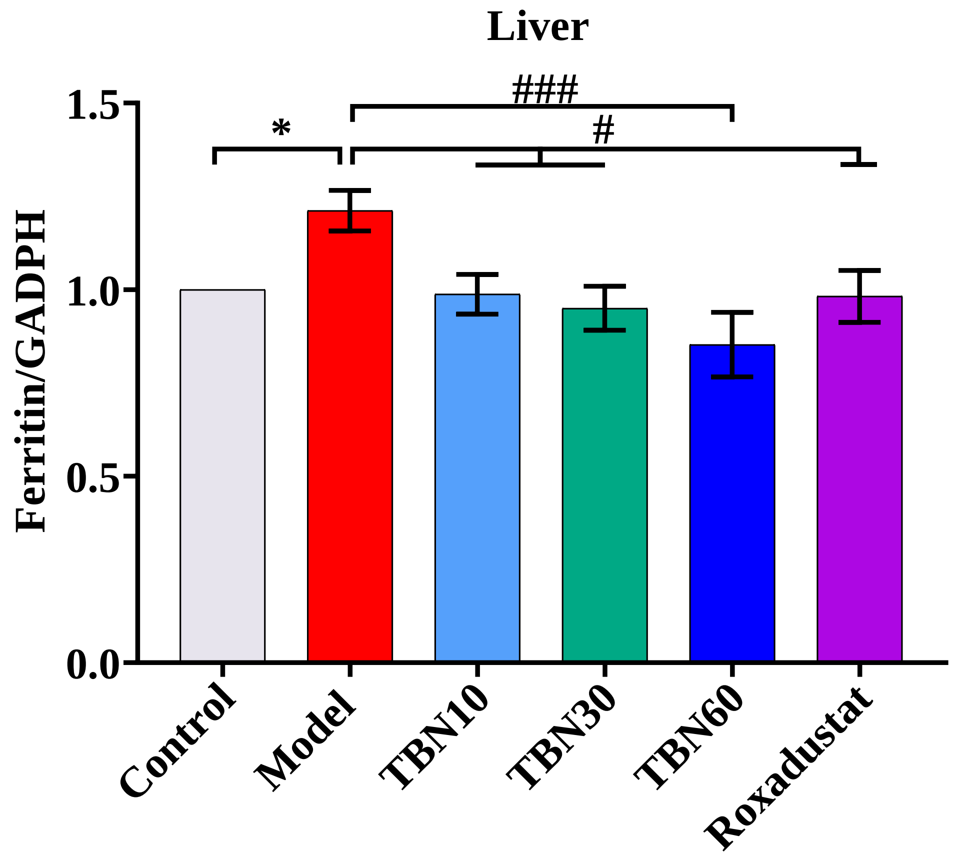
Liver
Ferritin/GADPH
1.5
1.0
0.5
0.0
*
###
#
Control
Model
TBN10
TBN30
TBN60
Roxadustat

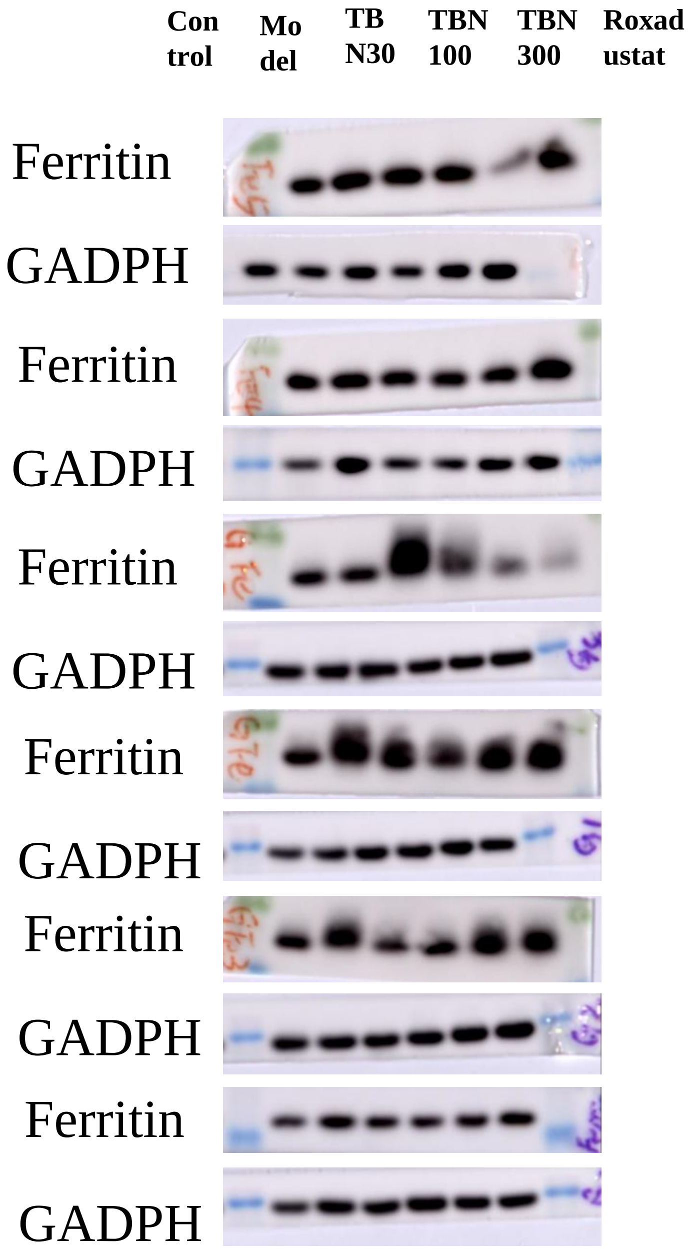
Con trol
Mo del
TB N30
TBN 100
TBN 300
Roxad ustat
Ferritin
GADPH
Ferritin
GADPH
Ferritin
GADPH
Ferritin
GADPH
Ferritin
GADPH
Ferritin
GADPH

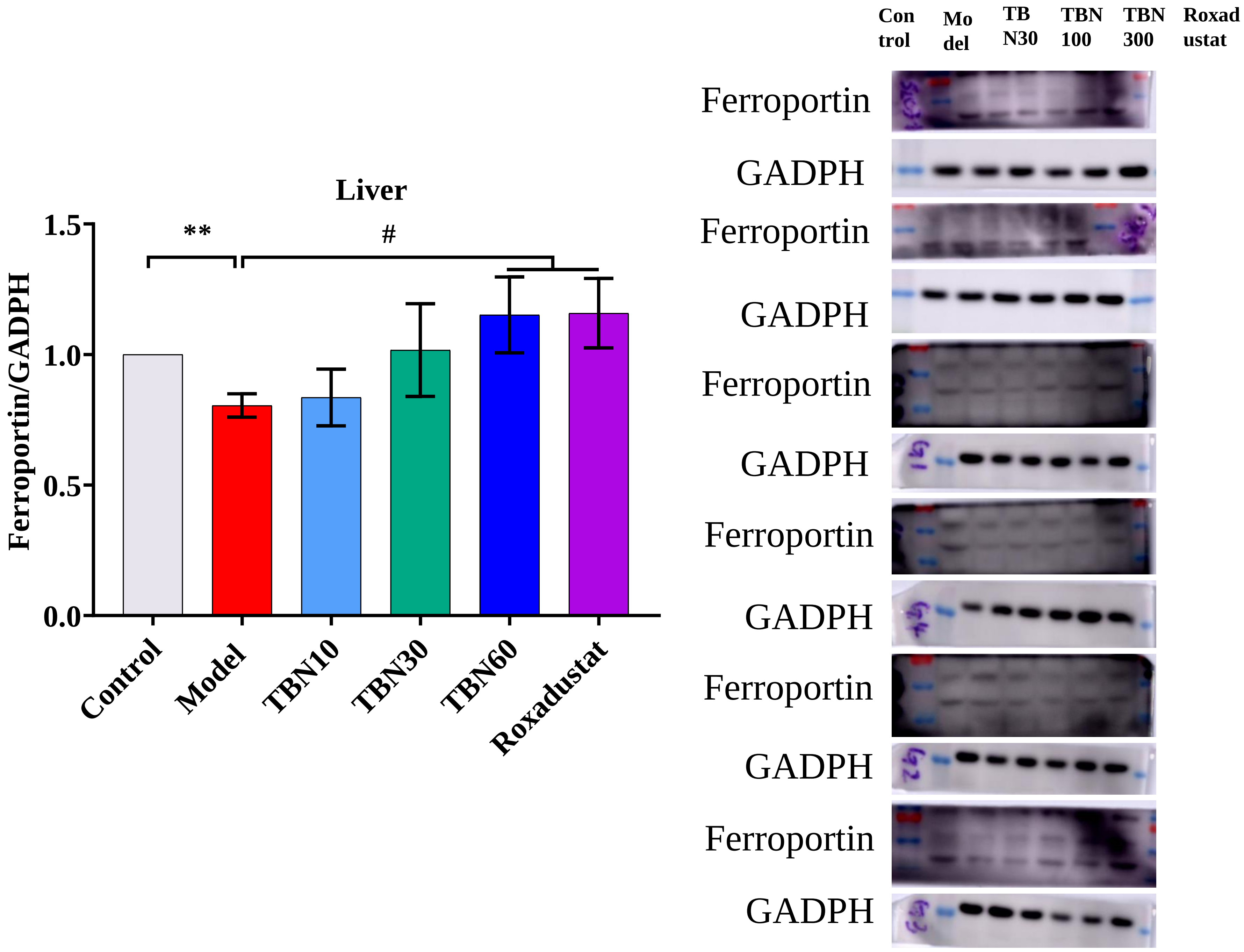

Liver
Ferroportin/GADPH
1.5
1.0
0.5
0.0
**
#
Control
Model
TBN10
TBN30
TBN60
Roxadustat
Con trol
Mo del
TB N30
TBN 100
TBN 300
Roxad ustat
Ferroportin
GADPH
Ferroportin
GADPH
Ferroportin
GADPH
Ferroportin
GADPH
Ferroportin
GADPH
Ferroportin
GADPH

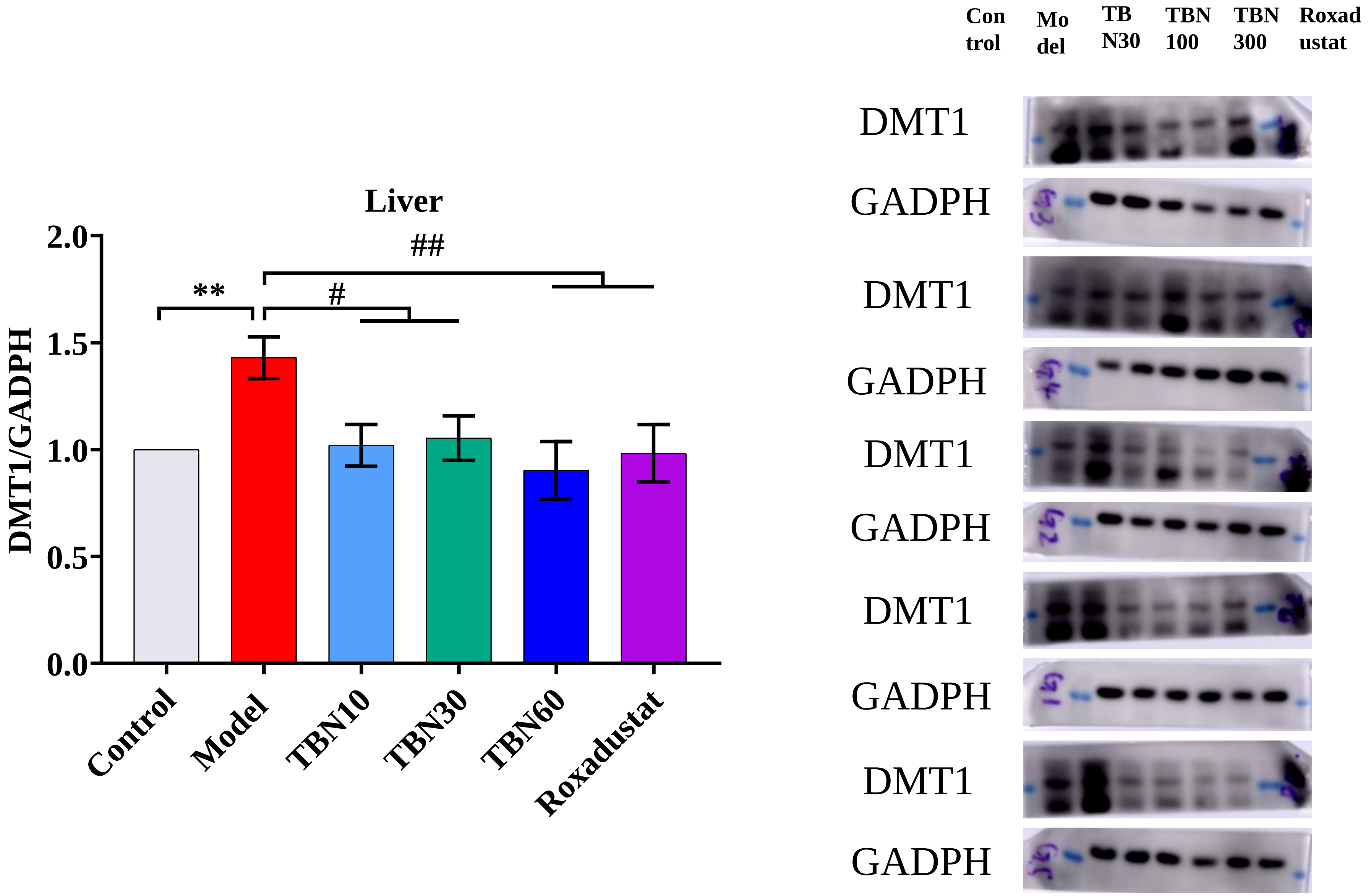
Liver
DMT1/GADPH
2.0
1.5
1.0
0.5
0.0
**
#
##
Control
Model
TBN10
TBN30
TBN60
Roxadustat
Con trol
Mo del
TB N30
TBN 100
TBN 300
Roxad ustat
DMT1
GADPH
DMT1
GADPH
DMT1
GADPH
DMT1
GADPH
DMT1
GADPH

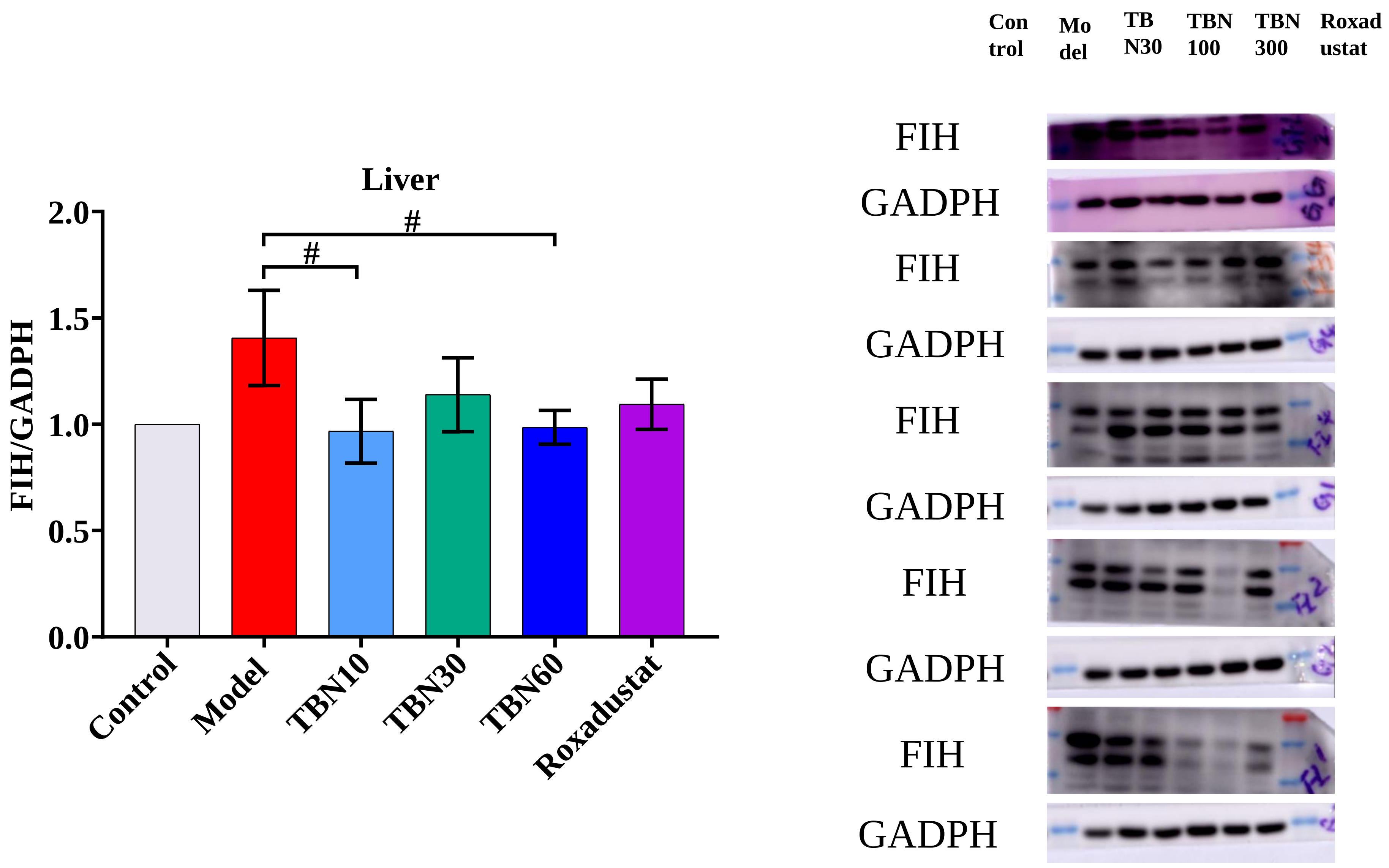
Liver
FIH/GADPH
2.0
1.5
1.0
0.5
0.0
#
#
Control
Model
TBN10
TBN30
TBN60
Roxadustat
Con trol
Mo del
TB N30
TBN 100
TBN 300
Roxad ustat
FIH
GADPH
FIH
GADPH
FIH
GADPH
FIH
GADPH
FIH
GADPH

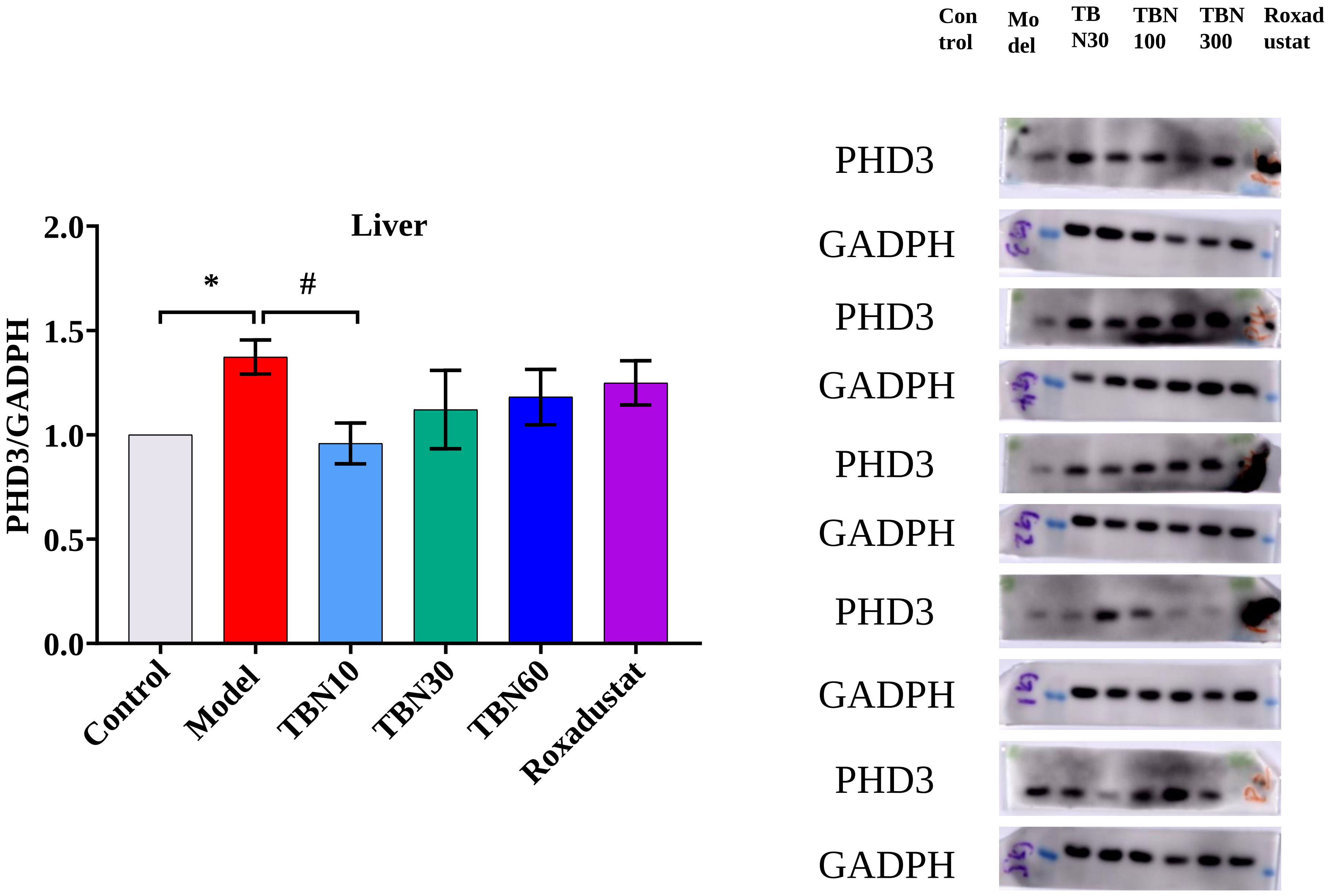
Liver
PHD3/GADPH
2.0
1.5
1.0
0.5
0.0
*
#
Control
Model
TBN10
TBN30
TBN60
Roxadustat
Con trol
Mo del
TB N30
TBN 100
TBN 300
Roxad ustat
PHD3
GADPH
PHD3
GADPH
PHD3
GADPH
PHD3
GADPH
PHD3
GADPH

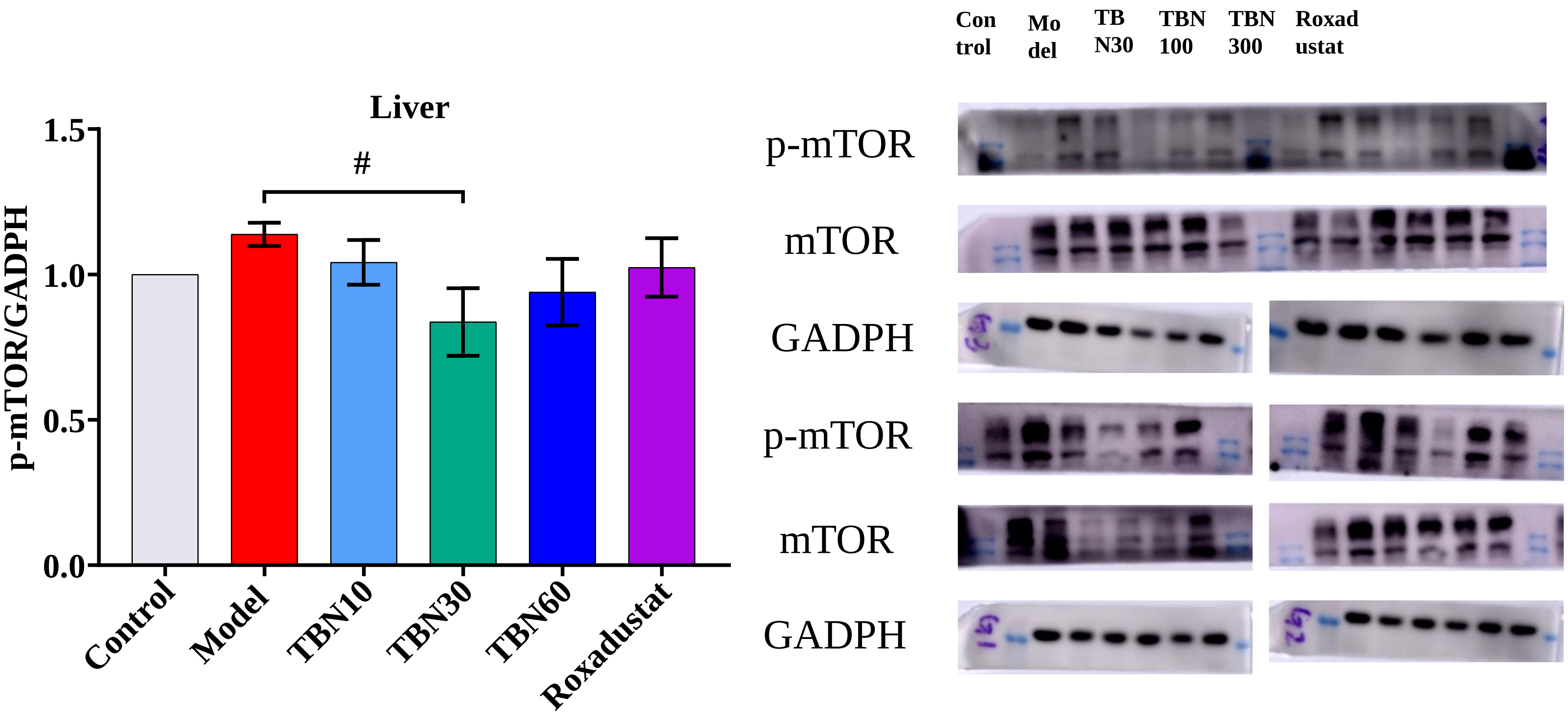

Liver
1.5
1.0
0.5
0.0
p-mTOR/GADPH
#
Control
Model
TBN10
TBN30
TBN60
Roxadustat
Con trol
Mo del
TB N30
TBN 100
TBN 300
Roxad ustat
p-mTOR
mTOR
GADPH
p-mTOR
mTOR
GADPH

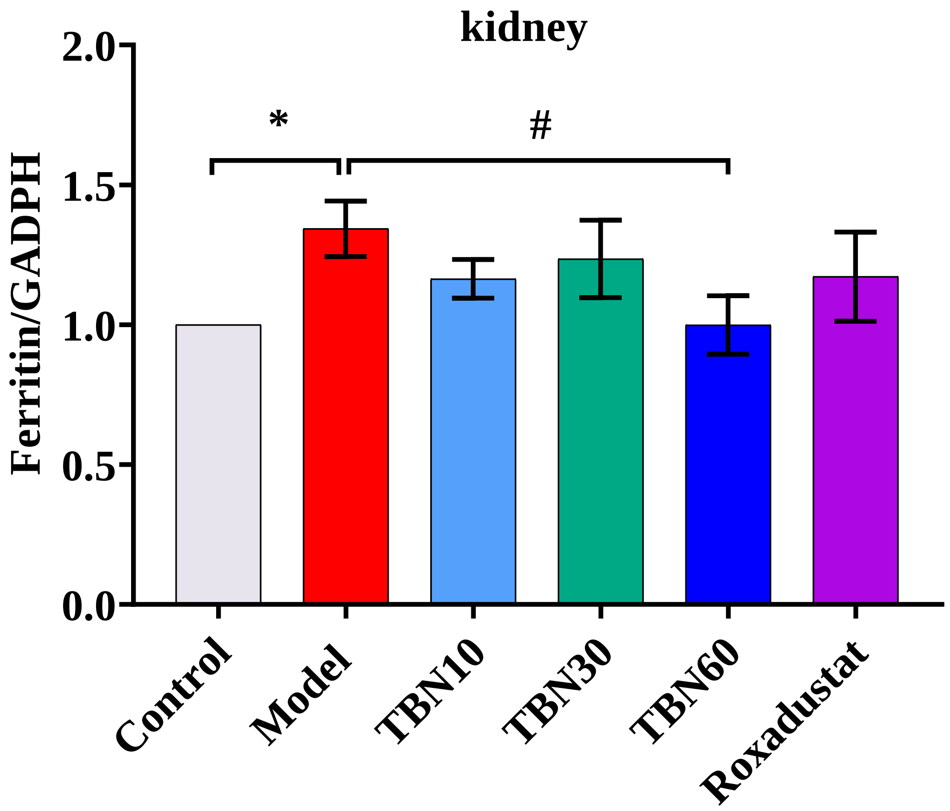

kidney
Ferritin/GADPH
2.0
1.5
1.0
0.5
0.0
*
#
Control
Model
TBN10
TBN30
TBN60
Roxadustat

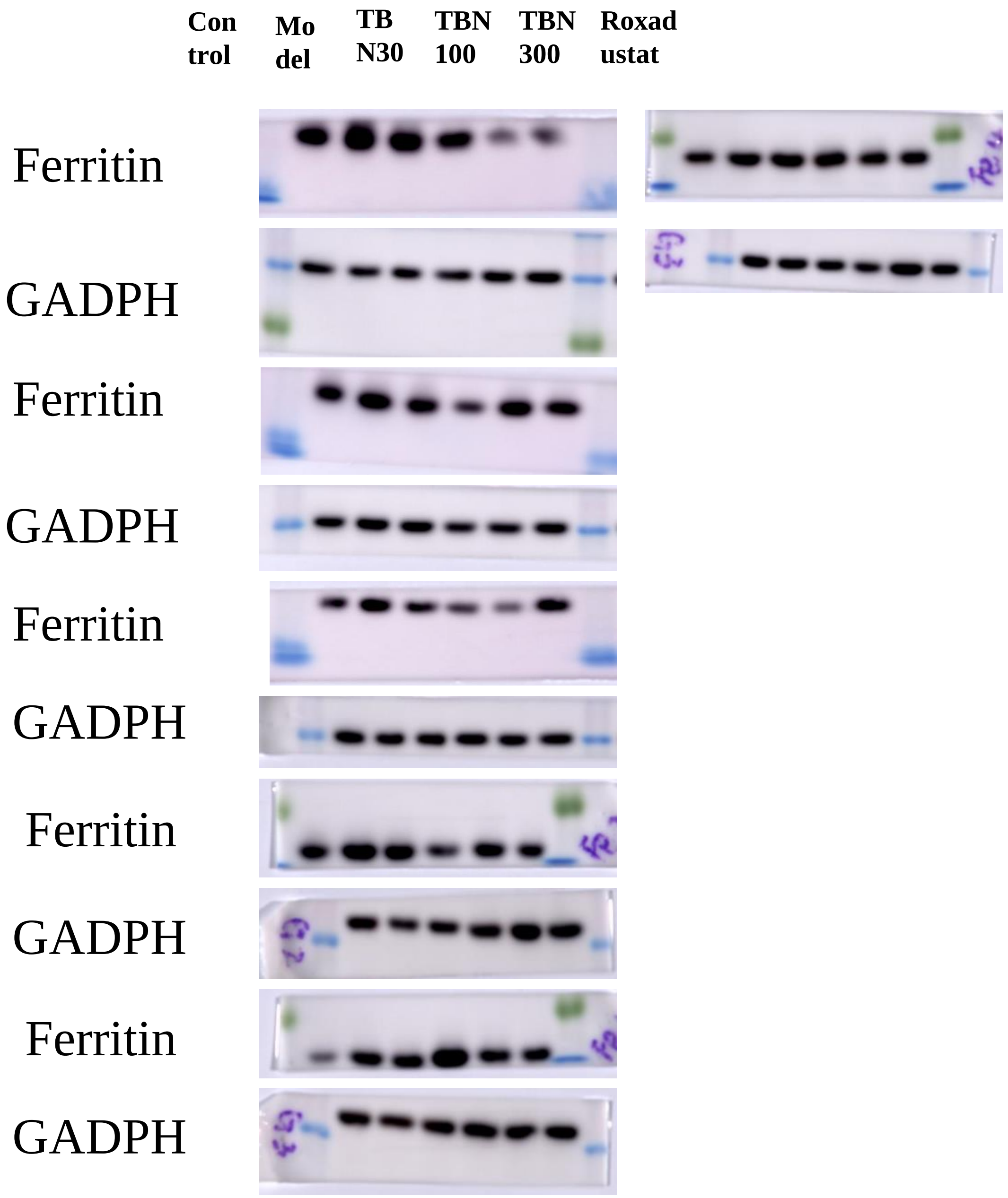

Con trol
Mo del
TB N30
TBN 100
TBN 300
Roxad ustat
Ferritin
GADPH
Ferritin
GADPH
Ferritin
GADPH
Ferritin
GADPH
Ferritin
GADPH

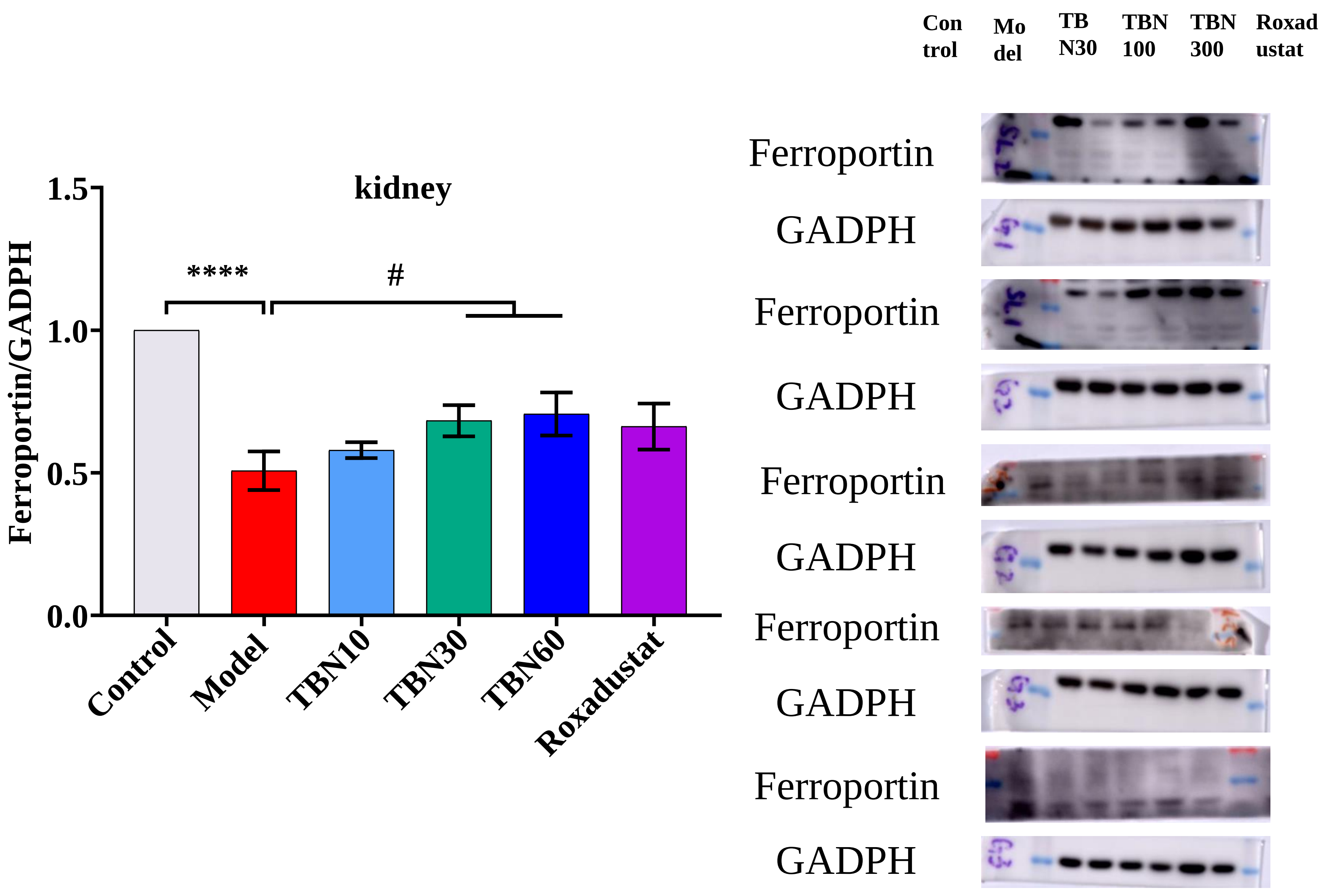
kidney
Ferroportin/GADPH
1.5
1.0
0.5
0.0
****
#
Control
Model
TBN10
TBN30
TBN60
Roxadustat
Con trol
Mo del
TB N30
TBN 100
TBN 300
Roxad ustat
Ferroportin
GADPH
Ferroportin
GADPH
Ferroportin
GADPH
Ferroportin
GADPH
Ferroportin
GADPH

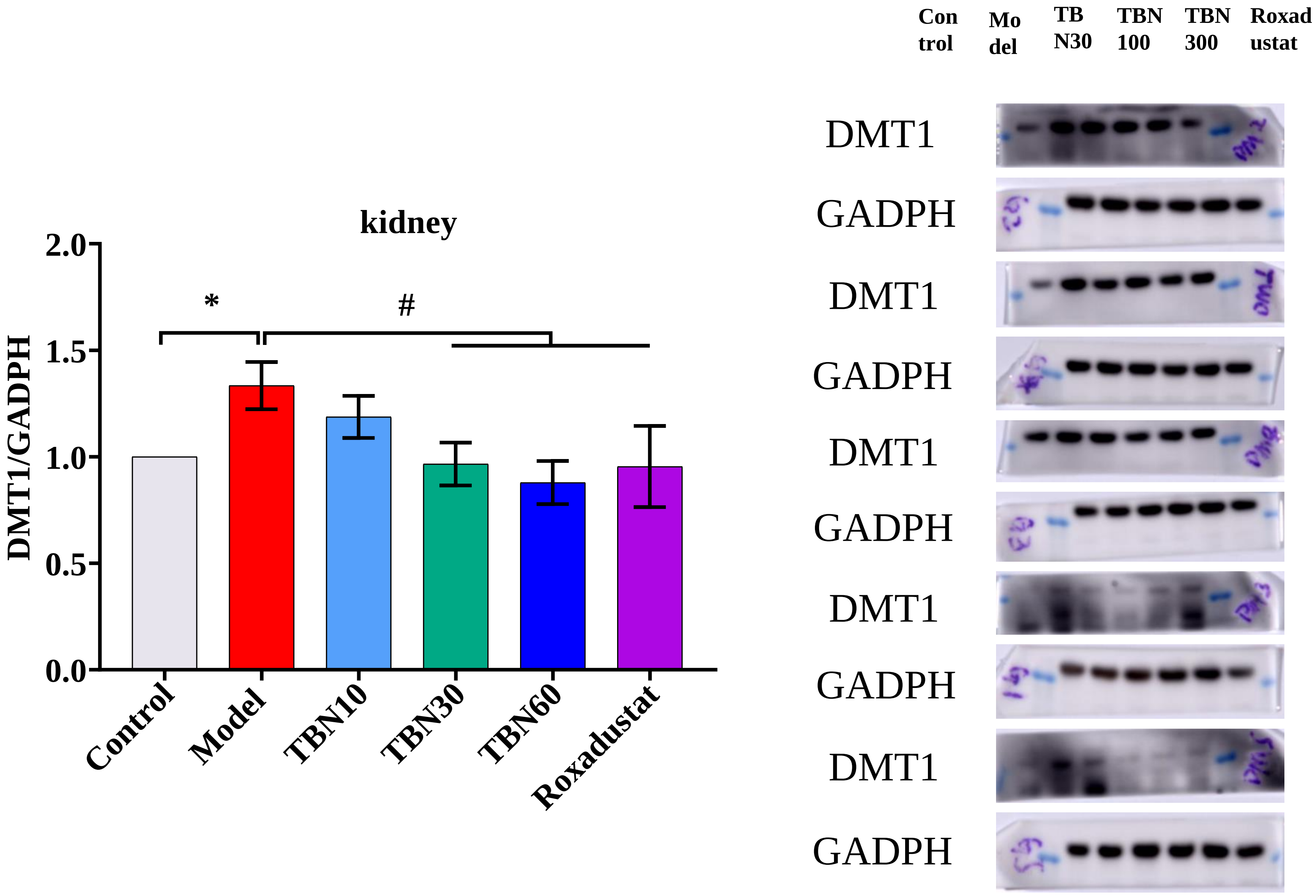
kidney
DMT1/GADPH
2.0
1.5
1.0
0.5
0.0
*
#
Control
Model
TBN10
TBN30
TBN60
Roxadustat
Con trol
Mo del
TB N30
TBN 100
TBN 300
Roxad ustat
DMT1
GADPH
DMT1
GADPH
DMT1
GADPH
DMT1
GADPH
DMT1
GADPH

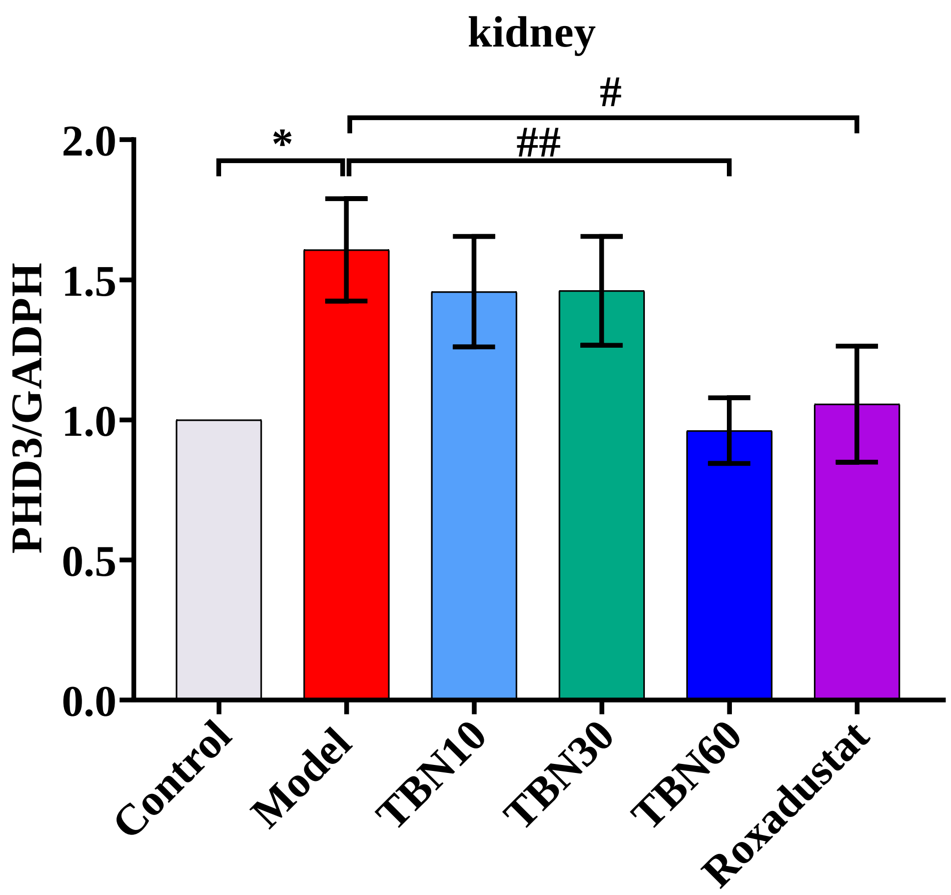
kidney
#
*
##
2.0
1.5
1.0
0.5
0.0
PHD3/GADPH
Control
Model
TBN10
TBN30
TBN60
Roxadustat

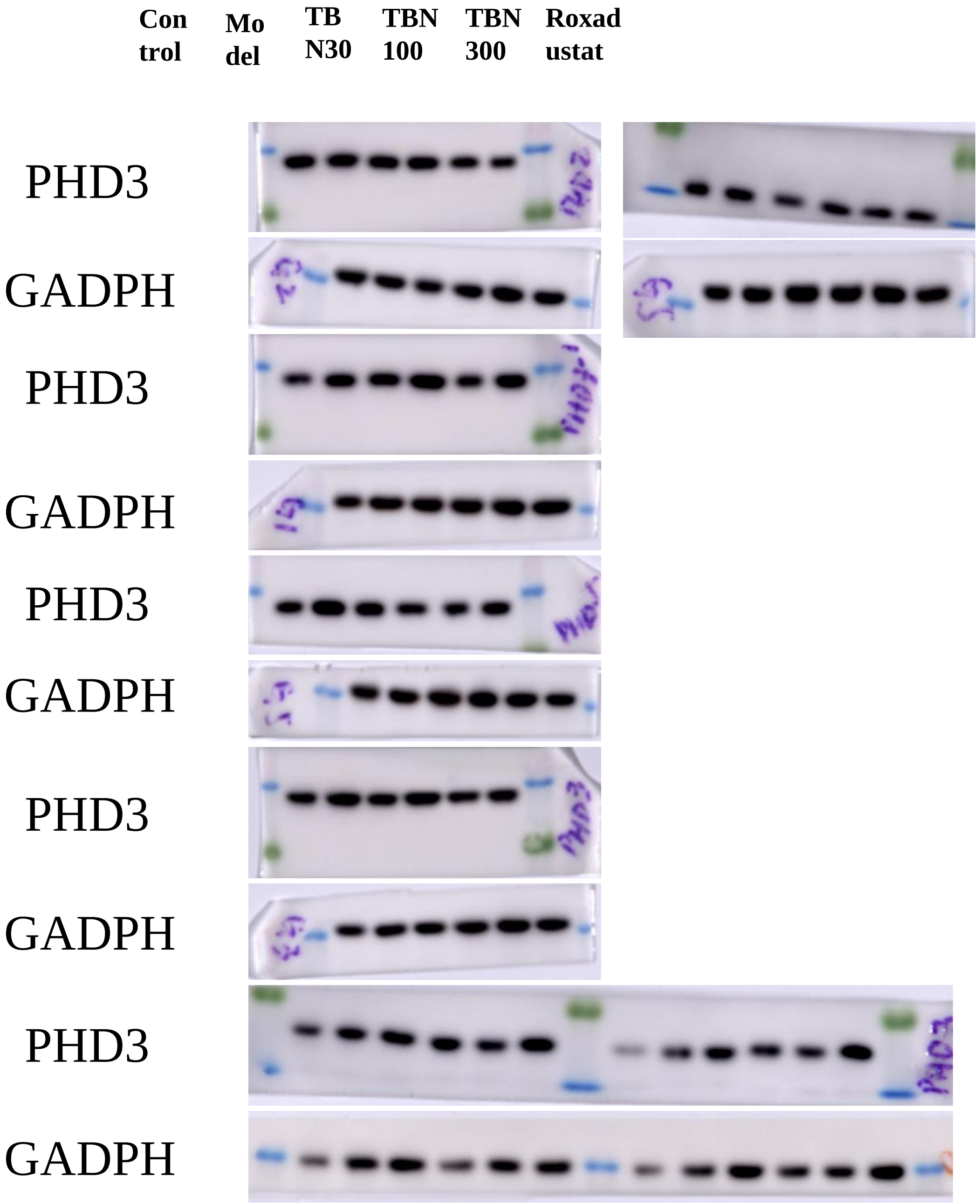
Con trol
Mo del
TB N30
TBN 100
TBN 300
Roxad ustat
PHD3
GADPH
PHD3
GADPH
PHD3
GADPH
PHD3
GADPH
PHD3
GADPH

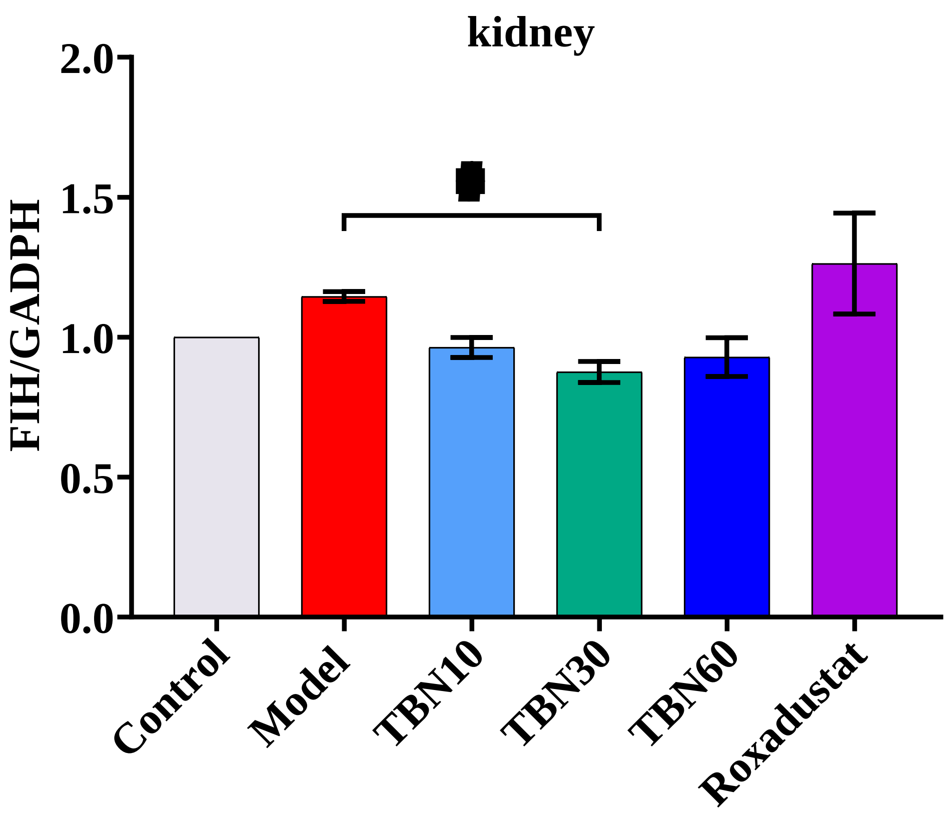
kidney
FIH/GADPH
2.0
1.5
1.0
0.5
0.0
Control
Model
TBN10
TBN30
TBN60
Roxadustat

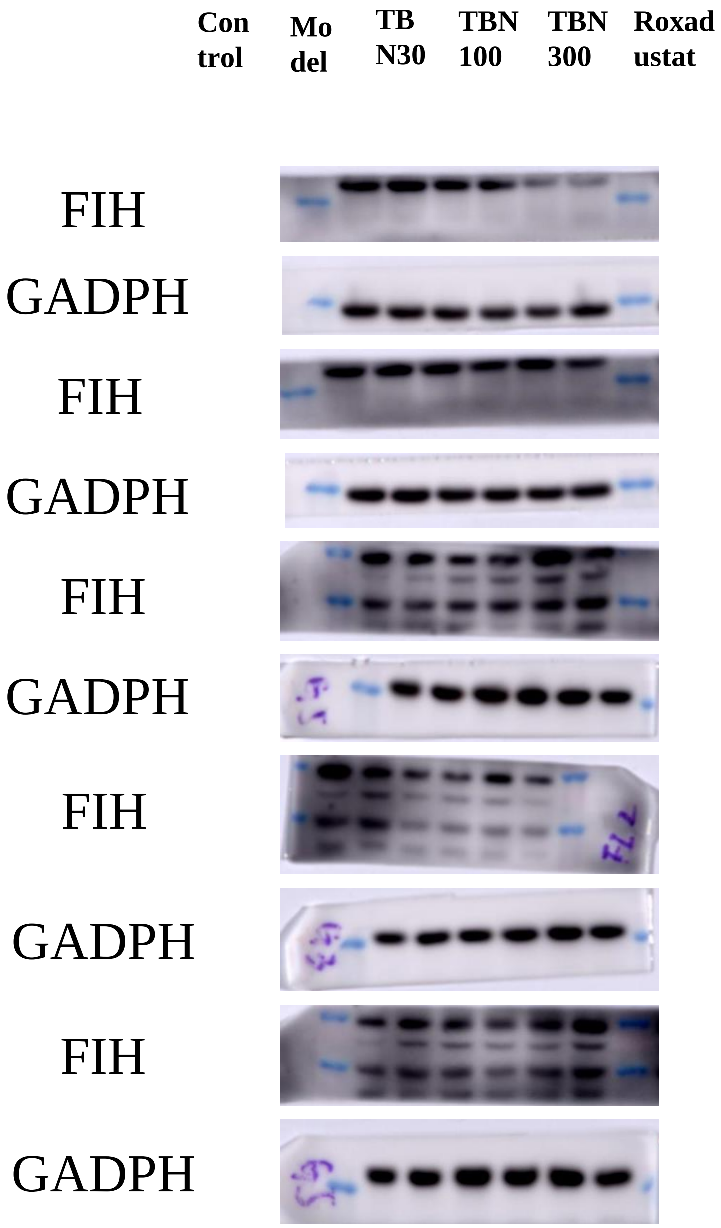
Con trol
Mo del
TB N30
TBN 100
TBN 300
Roxad ustat
FIH
GADPH
FIH
GADPH
FIH
GADPH
FIH
GADPH
FIH
GADPH

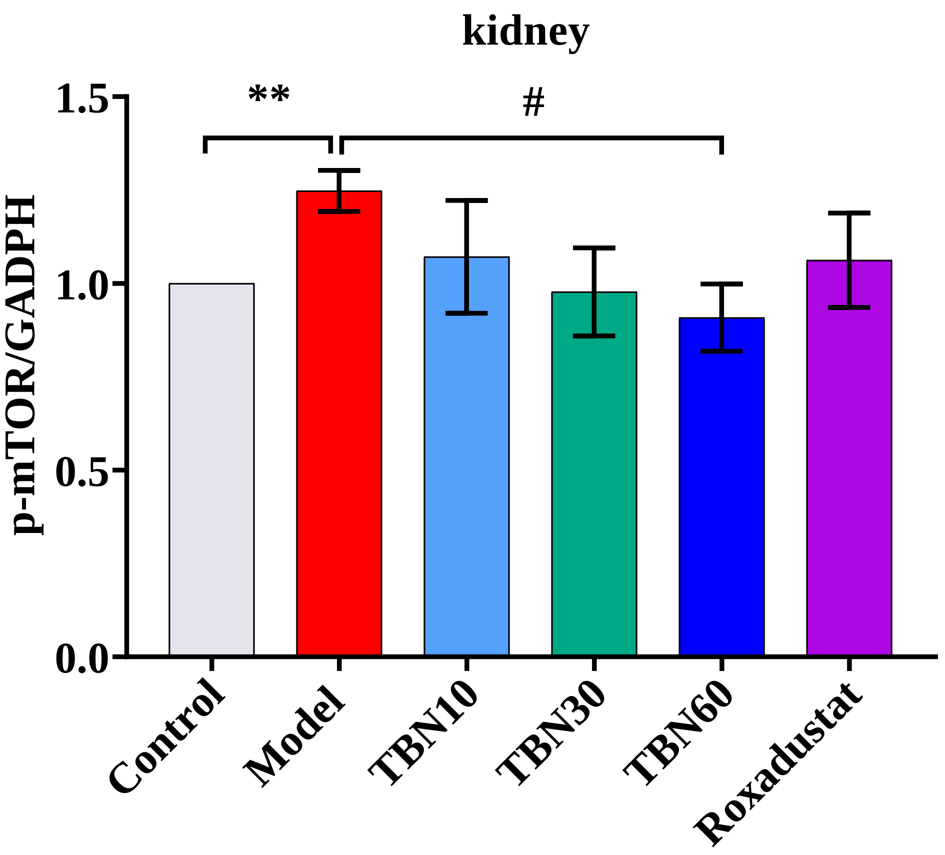
kidney
1.5
1.0
0.5
0.0
**
#
p-mTOR/GADPH
Control
Model
TBN10
TBN30
TBN60
Roxadustat

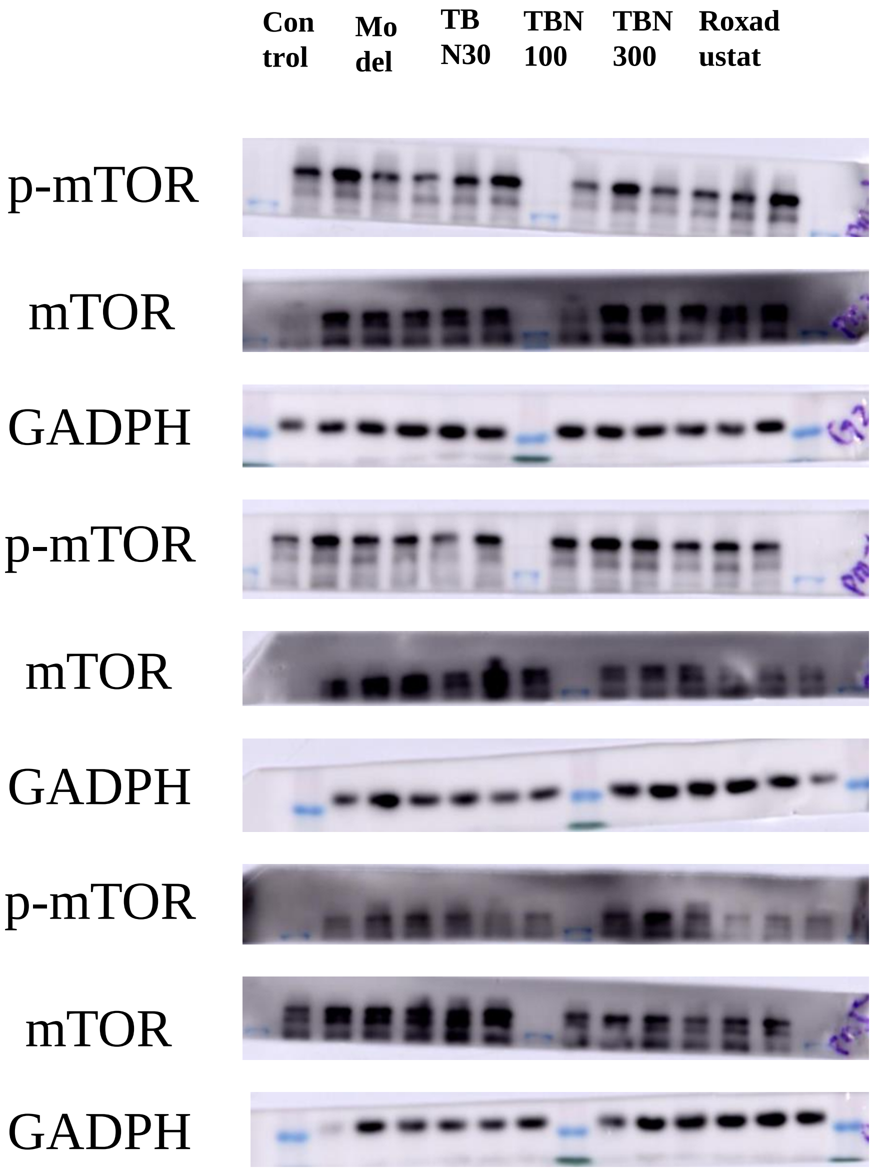
Con
trol
Mo
del
TB
N30
TBN
100
TBN
300
Roxad
ustat
p-mTOR
mTOR
GADPH
p-mTOR
mTOR
GADPH
p-mTOR
mTOR
GADPH
