# Supplementary material for: Tetramethylpyrazine nitrone activates hypoxia-inducible factor and regulates iron homeostasis to improve renal anemia
Source: Front Pharmacol. 2022 Oct 17;13:964234. doi: 10.3389/fphar.2022.964234 (PMC9618660; doi:10.3389/fphar.2022.964234)
Supplement: Supplementary file 2 [file DataSheet3.PDF]

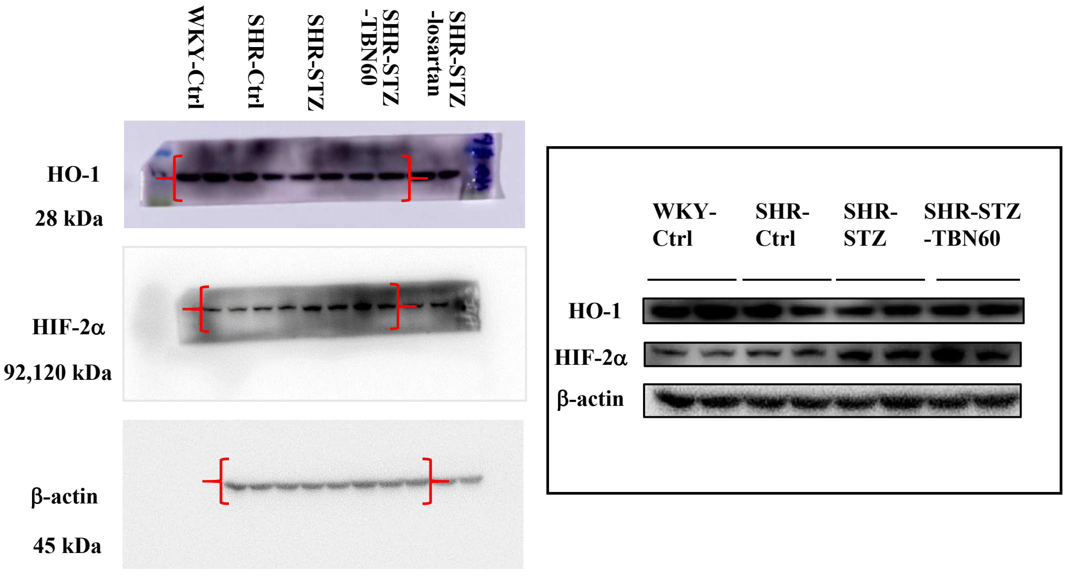

Supplementary Figure 1. Full scans of Western Blots from Figure 3C. The cropped Western Blots as they appear in Figure 3C are shown along the full ancropped originals.

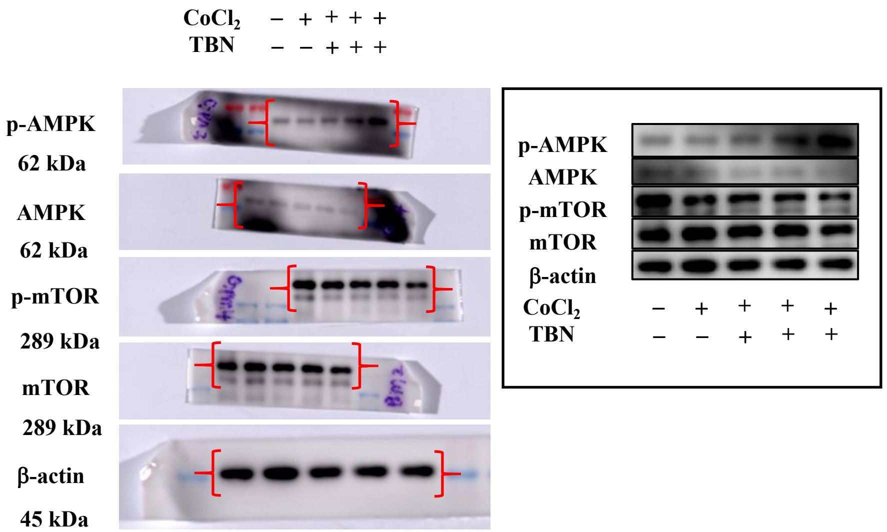

Supplementary Figure 2. Full scans of Western Blots from Figure 4A. The cropped Western Blots as they appear in Figure 4A are shown along the full ancropped originals.

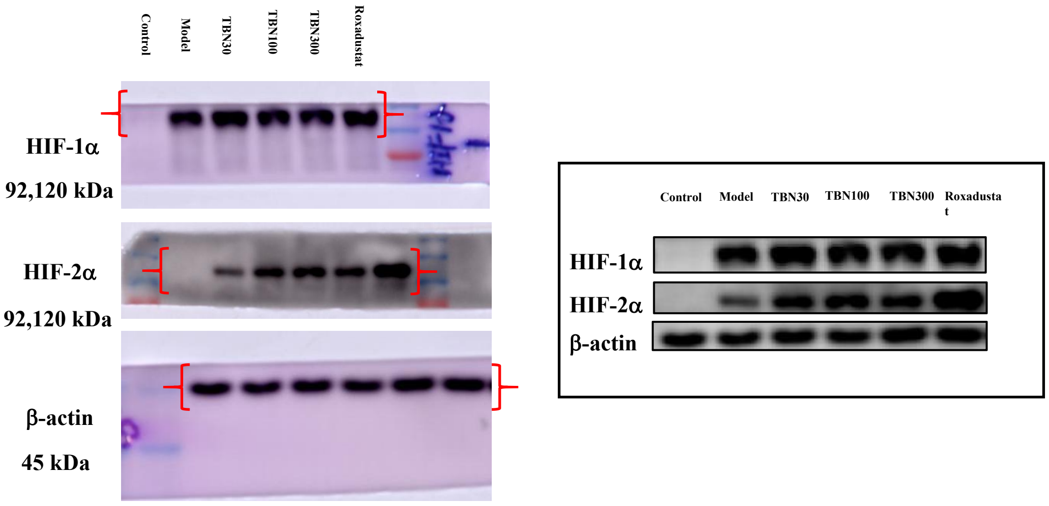

Supplementary Figure 3. Full scans of Western Blots from Figure 4D. The cropped Western Blots as they appear in Figure 4D are shown along the full ancropped originals.

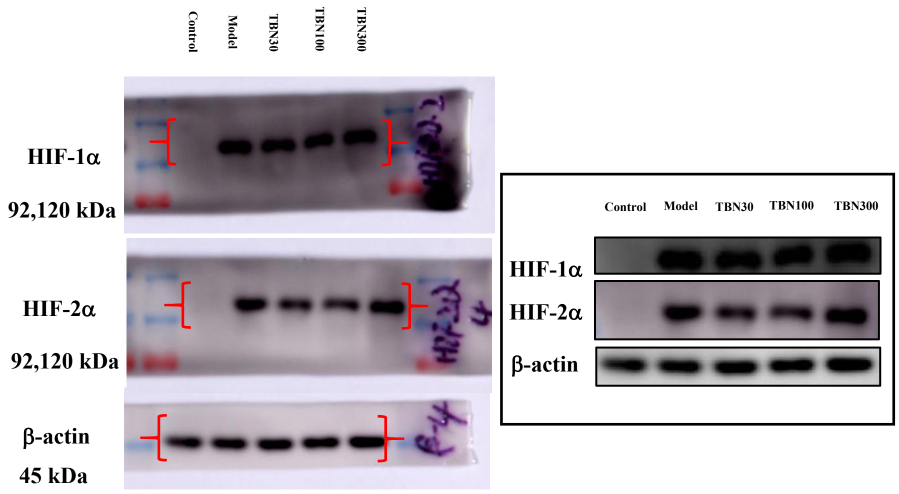

Supplementary Figure 4. Full scans of Western Blots from Figure 4G. The cropped Western Blots as they appear in Figure 4G are shown along the full ancropped originals.

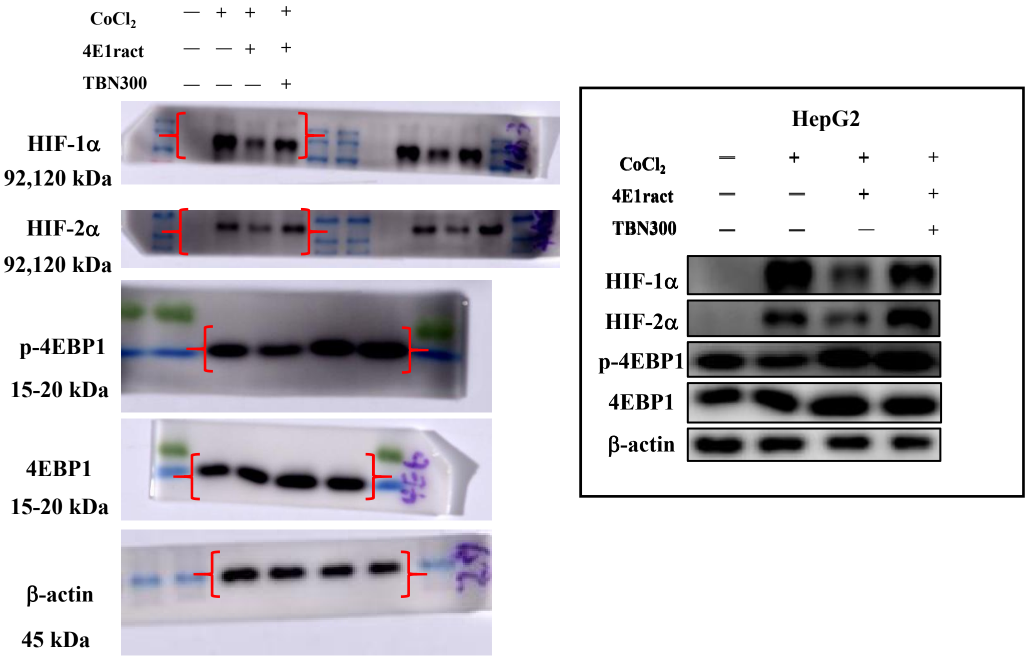

Supplementary Figure 5. Full scans of Western Blots from Figure 4L. The cropped Western Blots as they appear in Figure 4L are shown along the full ancropped originals.

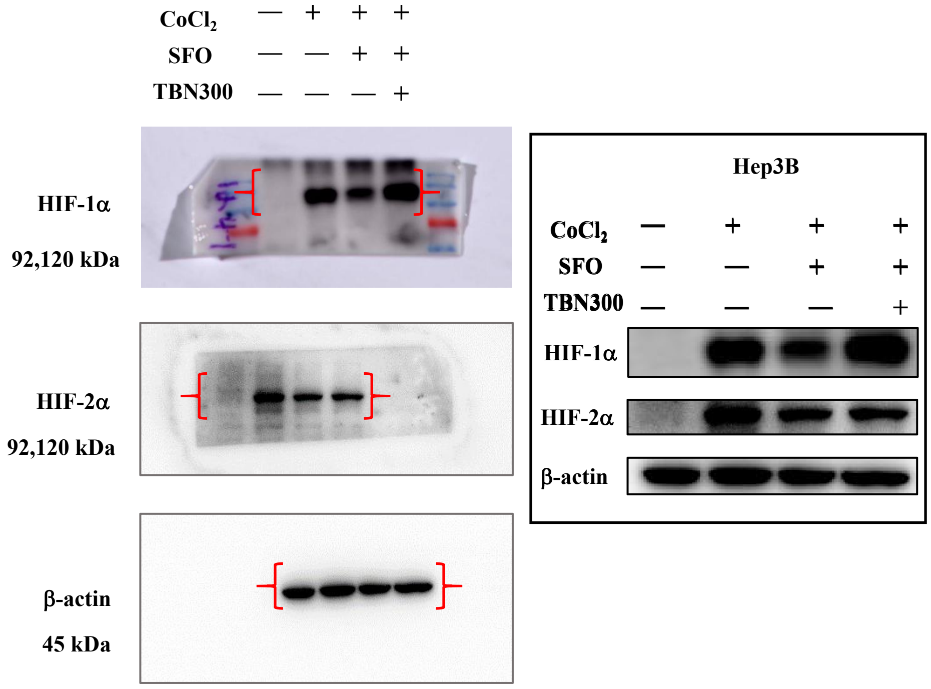

Supplementary Figure 6. Full scans of Western Blots from Figure 5A. The cropped Western Blots as they appear in Figure 5A are shown along the full ancropped originals.

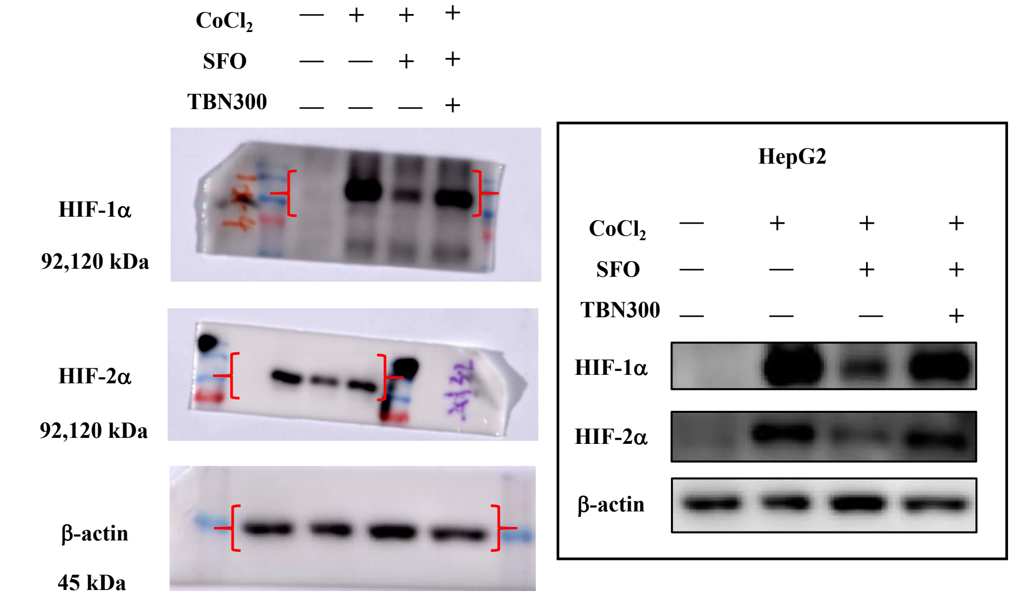

Supplementary Figure 7. Full scans of Western Blots from Figure 5D. The cropped Western Blots as they appear in Figure 5D are shown along the full ancropped originals.

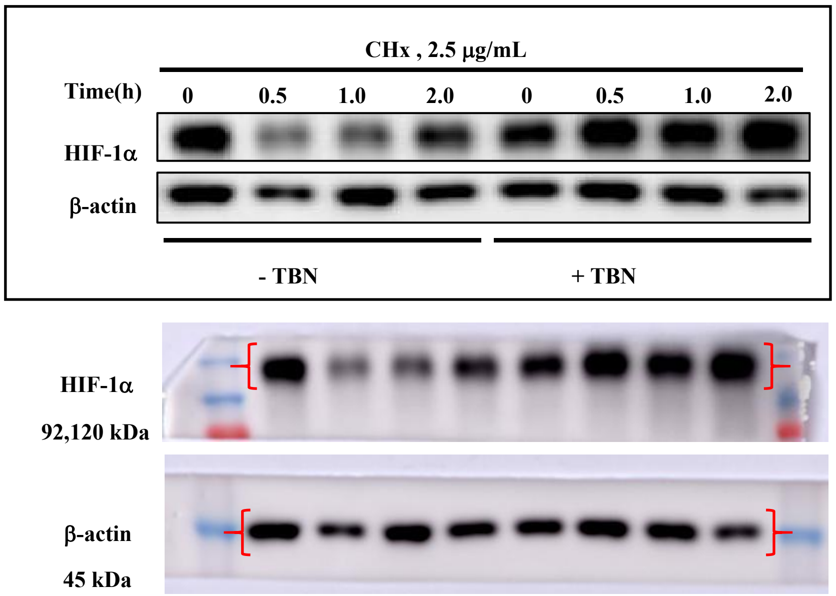

Supplementary Figure 8. Full scans of Western Blots from Figure 5G. The cropped Western Blots as they appear in Figure 5G are shown along the full ancropped originals.

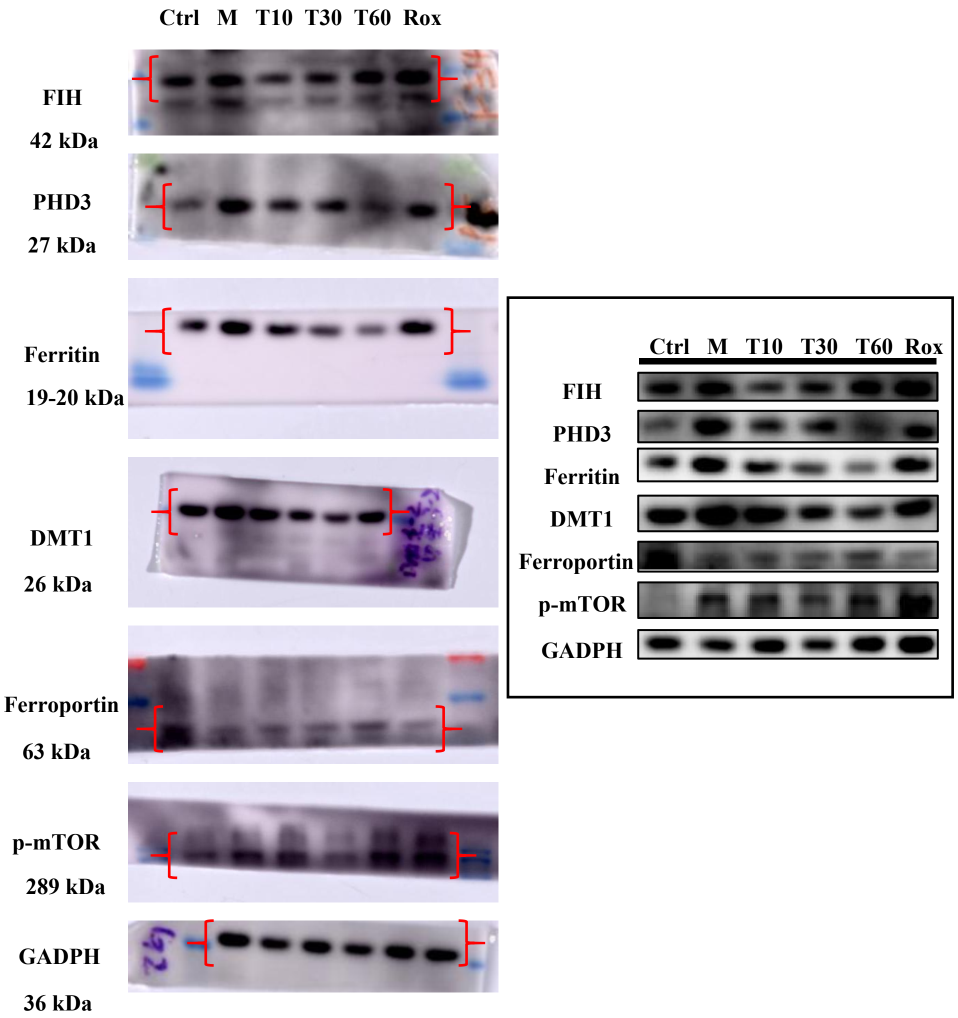

Supplementary Figure 9. Full scans of Western Blots from Figure 6A. The cropped Western Blots as they appear in Figure 6A are shown along the full ancropped originals.

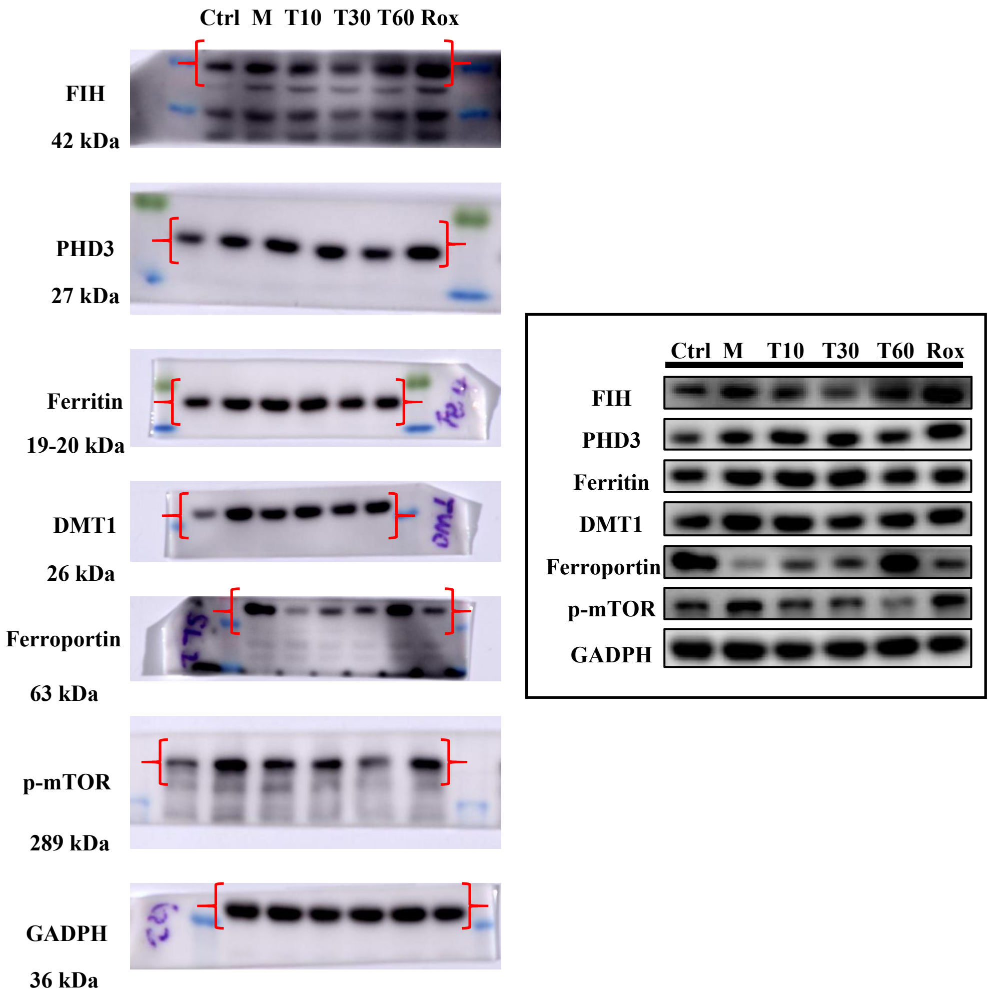

Supplementary Figure 10. Full scans of Western Blots from Figure 6H. The cropped Western Blots as they appear in Figure 6H are shown along the full ancropped originals.
