# Supplementary material for: Tetramethylpyrazine nitrone activates hypoxia-inducible factor and regulates iron homeostasis to improve renal anemia
Source: Front Pharmacol. 2022 Oct 17;13:964234. doi: 10.3389/fphar.2022.964234 (PMC9618660; doi:10.3389/fphar.2022.964234)
Supplement: Supplementary file 3 [file DataSheet1.PDF]

Supplementary Material

1 Supplementary Figures

1.1 Supplementary Figure 1

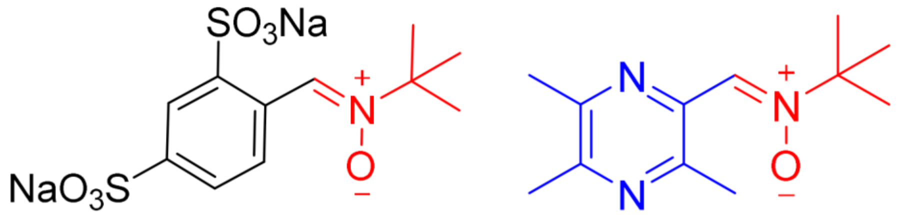

Supplementary Figure 1. The chemical structures of TMP, NXY-059, and TBN.

1.2 Supplementary Figure 2

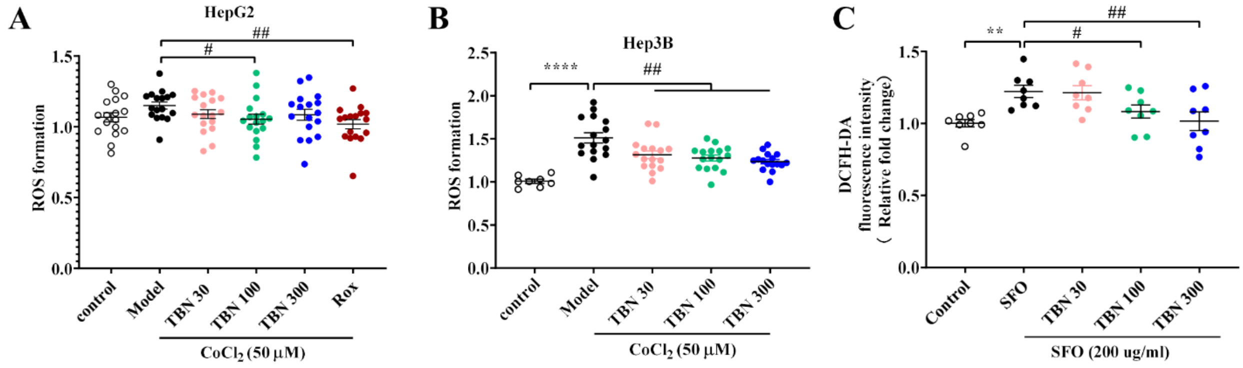

Supplementary Figure 2. Effect of TBN on the level of ROS in $CoCl_2$ or SFO-induced cells. (A) TBN decreases $CoCl_2$-induced accumulation of ROS in HepG2 cells. (B) TBN decreases $CoCl_2$-induced accumulation of ROS in Hep3B cells. (C) TBN decreases SFO-induced accumulation of Rosin Hep3B cell. $^{\#}P < 0.05$, $^{\#\#}P < 0.01$ vs model group, $^{**}P < 0.01$, $^{****}P < 0.0001$ vs control group.

1.3 Supplementary Figure 3

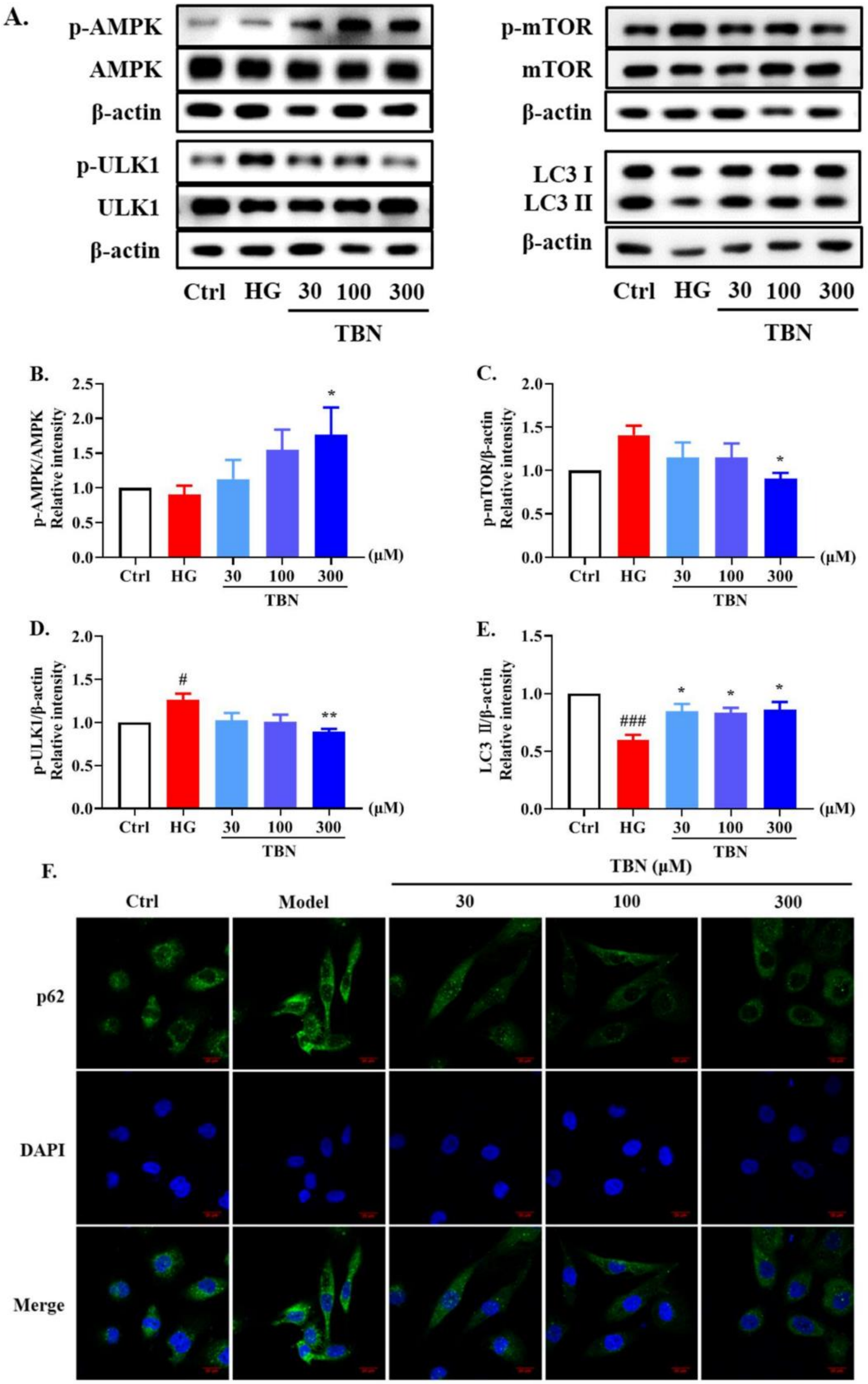

Supplementary Figure 3. Effect of TBN on the expression of p-AMPK, p-mTOR, p-ULK1, p62 and LC3 II in high glucose-induced HK-2cells. (A) Representative Immunoblots of p-AMPK, p-mTOR, p-ULK1 and LC3 II in HK-2 cells in various groups. n=4. (B-E) Bar graphs showing the fold change in protein expression after normalization to the internal control β-actin. n=4. (F)

Representative Immunocytochemical images of p62 staining in HK-2 cells. Scale bar, 20 µm. $^{\#}P <$ 0.05, $^{\#\#\#}P < 0.001$ vs Ctrl group, ***P** < 0.05, ****P** < 0.01 vs HG group.

1.4 Supplementary Figure 4

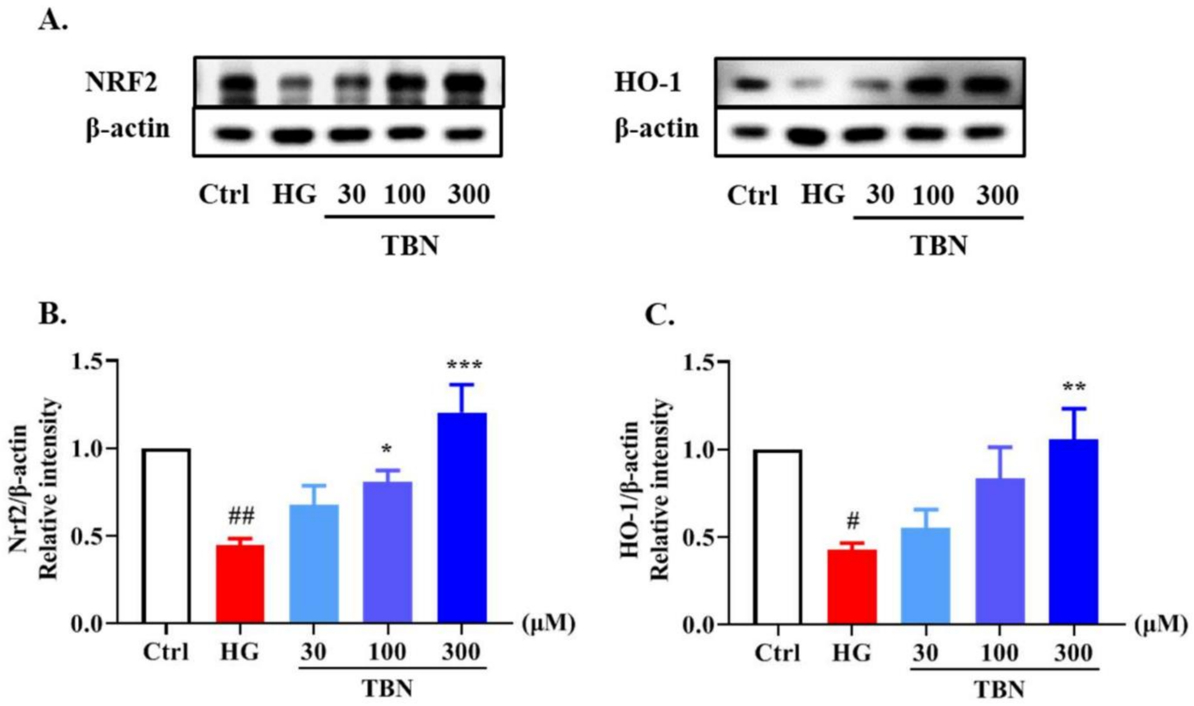

Supplementary Figure 4. Effect of TBN on the expression of NRF2 and HO-1 in high glucose-induced HK-2 cells. (A) Representative Immunoblots of NRF2, HO-1 in HK-2 cells in various groups. n=4. (B-C) are bar graphs showing the fold change in protein expression after normalization to the internal control β-actin. n=4. $^{\#}P < 0.05$, $^{\#\#}P < 0.01$ vs Ctrl group, ***P** < 0.05, ****P** < 0.01, *****P** < 0.001 vs HG group.
